# Supplementary material for: Pregnancy of unknown location: external validation of the hCG-based M6NP and M4 prediction models in an emergency gynaecology unit
Source: BMJ Open. 2022 Nov 29;12(11):e058454. doi: 10.1136/bmjopen-2021-058454 (PMC9716941; doi:10.1136/bmjopen-2021-058454)
Supplement: Supplementary data [file bmjopen-2021-058454supp004.pdf]

Supplemental

Supplemental Table 1. Covariates used in the original M4 [1] and M6NP [2] cohorts.

Model	Covariate	Description
M4	log(average hCG)	Log transformation of (first hCG + second hCG)/2
M4	centered hCG ratio	Centering transformation of (second hCG /first hCG-1.17)
M4	(centered hCG ratio)^2	The quadratic effect was added because the linearity for the logit assumption was violated for hCG ratio.
M6NP	log(initial hCG)	Log transformation of the first hCG
M6NP	log(hCG ratio)	Log transformation of (second hCG/first hCG)
M6NP	[log(hCG ratio)]^2	Square of the log transformed hCG ratio

Log refers to the natural logarithm. hCG = human chorionic gonadotropin.

[a]Average hCG ratio in the original M4 cohort. Centering reduced the correlation between hCG ratio and its square from 0.96 to 0.59.

Linear predictor functions for M4 where IUP was reference category [1]:

$$z_1 = log\left(\frac{P(FPUL)}{P(IUP)}\right)$$

$$= +5.8803$$

$$-5.5594 \times (hCG\ ratio - 1.17)$$

$$+2.0504 \times (hCG\ ratio - 1.17)^2$$

$$-1.175 \times \log(average\ hCG)$$

$$z_2 = log\left(\frac{P(EP)}{P(IUP)}\right)$$

$$= 0.3857$$

$$-0.2627 \times (hCG\ ratio - 1.17)$$

$$-3.9319 \times (hCG\ ratio - 1.17)^2$$

$$-0.0572 \times \log(average\ hCG)$$

Linear predictor functions for M6NP where EP was reference category [2]:

$$z_1 = log\left(\frac{P(FPUL)}{P(EP)}\right)$$

$$= 2.5506$$

$$-0.4242 \times log(initial\ hCG)$$

$$-2.9502 \times log(hCG\ ratio)$$

$$+2.1765 \times [log(hCG\ ratio)]^2$$

$$z_2 = log\left(\frac{P(IUP)}{P(EP)}\right)$$

$$= -3.2842$$

$$+0.4072 \times log(initial\ hCG)$$

$$+1.9238 \times log(hCG\ ratio)$$

$$+2.8952 \times [log(hCG\ ratio)]^2$$

By using the exponentiated linear predictor functions of M4 and M6NP respectively the probability of each outcome are computed as:

$$P(FPUL) = \frac{exp(z_1)}{1 + exp(z_1) + exp(z_2)}$$

$$P(IUPor\ EP\ ) = \frac{exp(z_2)}{1 + exp(z_1) + exp(z_2)}$$

$$P(EPor\ IUP) = \frac{1}{1 + exp(z_1) + exp(z_2)}$$

The probability of IUP is computed with the third formula when M4 is used. If M6NP is used the third formula computes the probability of EP. Log in the linear predictor functions refers to the natural logarithm. PUL, pregnancy of unknown location; FPUL, failed PUL; IUP, intrauterin pregnancy, EP, ectopic pregnancy.

Equation of Net Benefit [3]:

$\text{Net benefit} = (\text{True positive})/n \ - \ (\text{False positive})/n \cdot ((P_t/(1 - P_t))$, where true positive is the number of EP classified as high risk; false positive is the number of non-EP classified as high risk and n is the total number of patients. In the equation false positives are weighed by the odds of the selected risk threshold (P_t) for high risk classification. If a 5% risk threshold is chosen, a false positive non-EP is valued at 1/19th of a true positive EP.

References:

1. Condous G, Van Calster B, Kirk E, Haider Z, Timmerman D, Van Huffel S, et al. Prediction of ectopic pregnancy in women with a pregnancy of unknown location. Ultrasound Obstet Gynecol 2007; 29: 680-7.
2. Van Calster B, Bobdiwala S, Guha S, Van Hoorde K, Al-Memar M, Harvey R, et al. Managing pregnancy of unknown location based on initial serum progesterone and serial serum hCG levels: development and validation of a two-step triage protocol. Ultrasound Obstet Gynecol 2016; 48: 642-49.
3. Steyerberg EW, Vickers AJ, Cook NR, Gerds T, Gonen M, Obuchowski N, et al. Assessing the performance of prediction models: a framework for traditional and novel measures. Epidemiology 2010; 21: 128-38.

Supplemental Table 2. Descriptive statistics of 239 non-eligible patients.

Age (years)	
Median (Q1; Q3)	32 (26; 36)
Range	15–46
First hCG value (IU/L)	
Median (Q1; Q3)	1400 (310; 5300)
Range	3–78000
Type of PUL, n (%)	
True PUL	88 (37)
Probable IUP	85 (36)
Probable EP	66 (28)
Vaginal bleeding, n (%)	155 (65)
Prior ectopic pregnancy, n (%)	20 (8)
IUD, n (%)	0 (0)

hCG, human chorionic gonadotropin; Q1, first interquartile; Q3, third interquartile; PUL, pregnancy of unknown location; IUP, intrauterine pregnancy, EP, ectopic pregnancy; IUD, intrauterine device. Because of rounding the summarised percentage are not exactly 100%.

Supplemental Table 3. Descriptive statistics of failed PUL, intrauterine pregnancies and ectopic pregnancies in the validation cohort and the original M4 and M6NP cohorts.

Outcome of PUL/variable	Validation cohort	Original M4 cohort	Original M6NP cohort
Failed PUL[a]	461 (43)	109 (55)	785 (54)
Age (Years)[b]	31 (27; 35)	30 (7)	32 (27; 36)
First hCG value (IU/L) [b]	334 (120; 1078)	N/A[c]	308 (104; 975)
Second hCG value (IU/L) [b]	158 (53; 460)	N/A[c]	143 (48; 397)
hCG ratio[b]	0.42 (0.27; 0.64)	0.35 (0.27)	0.40 (0.28; 0.58)
hCG average (IU/L)[b]	261 (93; 790)	89 (216)	N/A[d]
Intrauterine pregnancy[a]	362 (34)	76 (39)	501 (35)
Age (Years)[b]	29 (24; 33)	30 (6)	30 (24; 33)
First hCG value (IU/L) [b]	1521 (464; 5125)	N/A[c]	488 (253; 887)
Second hCG value (IU/L)[b]	2765 (983; 7900)	N/A[c]	1061 (515; 1863)
hCG ratio[b]	1.81 (1.39; 2.21)	2.12 (0.57)	2.15 (1.83; 2.50)
hCG average (IU/L)[b]	2226 (746; 6750)	727 (986)	N/A[d]
Ectopic pregnancy[a]	238 (22)	12 (6)	163 (11)
Age (Years)[b]	32 (27; 36)	32 (6.5)	32 (28; 35)
First hCG value (IU/L)[b]	810 (252; 2315)	N/A[c]	521 (200; 1220)
Second hCG value (IU/L)[b]	920 (265; 2735)	N/A[c]	604 (224; 1266)
hCG ratio[b]	1.05 (0.86; 1.31)	1.41 (0.47)	1.16 (0.96; 1.48)
hCG average (IU/L)[b]	865 (270; 2600)	621 (354)	N/A[d]

PUL, pregnancy of unknown location; hCG, human chorionic gonadotropin; Q1, first quartile; Q3, third quartile; IQR, interquartile range; N/A, not available.

[a]Reported as number (%)

[b]Reported as median (Q1; Q3) in the validation cohort and the original M6 cohort, and as median (IQR) in the original M4 cohort.

[c]The first and second hCG value were only presented as an average in the original M4 cohort.

[d]The average hCG value was not presented in the original M6 cohort.

Supplemental Table 4. Descriptive statistics of 12 ectopic pregnancies misclassified as low risk by M6NP.

Type of PUL	First hCG (IU/L)	Second hCG (IU/L)	M6NP prediction	Site of EP	Rupture	Treatment
True PUL	470	290	FPUL	Tubal	No	Salpingotomy
Probable EP	88	42	FPUL	Tubal	Yes	Salpingectomy
True PUL	619	1948	IUP	Tubal	No	Salpingectomy
True PUL	3700	1500	FPUL	Tubal	No	Salpingectomy
True PUL	700	220	FPUL	Tubal	No	Salpingectomy
Probable EP	420	210	FPUL	Tubal	No	Salpingectomy
True PUL	10000	20000	IUP	Tubal	No	Salpingectomy
True PUL	640	240	FPUL	Tubal	No	Salpingectomy
True PUL	43	170	IUP	Tubal	No	Salpingectomy
Probable EP	56	23	FPUL	Tubal	No	Salpingectomy
True PUL	130	330	IUP	Tubal	No	salpingectomy
True PUL	230	590	IUP	Tubal	No	Salpingectomy

hCG, human chorionic gonadotropin, PUL, pregnancy of unknown location; EP, ectopic pregnancy; FPUL, failed PUL; IUP, intrauterine pregnancy.

Supplemental Table 5. Descriptive statistics of patients (n=1186) in the sensitivity analysis.

Age (years)	
Median (Q1;Q3)	31 (26;35)
Range	15–49
First hCG value (IU/L)	
Median (Q1;Q3)	697 (210;2300)
Range	7–180000
Second hCG value (IU/L)	
Median (Q1;Q3)	615 (142;2655)
Range	1–190000
Hours between two hCG samples, n (%)	
<24	70 (6)
24–39	141 (12)
40–56	751 (63)
57–72	169 (14)
>72	55 (5)
Type of PUL, n (%)	
True PUL	880 (74)
Probable IUP	254 (21)
Probable EP	52 (4)
Vaginal bleeding, n (%)	803 (68)
Prior ectopic pregnancy, n (%)	86 (7)
IUD, n (%)	37 (3)

hCG, human chorionic gonadotropin; Q1, first interquartile; Q3, third interquartile; PUL, pregnancy of unknown location; IUP, intrauterine pregnancy, EP, ectopic pregnancy; IUD, intrauterine device. Because of rounding the summarised percentage sometimes are not exactly 100%.

Supplemental Table 6. Performance of M6NP and M4 at the 5% threshold in the sensitivity analysis.

Performance measure	M6NP	M4
AUC	0.84 (0.81–0.86)	0.80 (0.77–0.83)
Sensitivity, %	93 (90–96)	83 (78–87)
False positive rate, %	49 (48–53)	36 (33–39)
Negative predictive value, %	96 (94–98)	92 (90–94)

AUC, area under the receiver operating characteristic curve. 95% confidence interval (in parentheses).
