# Supplementary figures and images for: Pregnancy of unknown location: external validation of the hCG-based M6NP and M4 prediction models in an emergency gynaecology unit
Source: BMJ Open. 2022 Nov 29;12(11):e058454. doi: 10.1136/bmjopen-2021-058454 (PMC9716941; doi:10.1136/bmjopen-2021-058454)

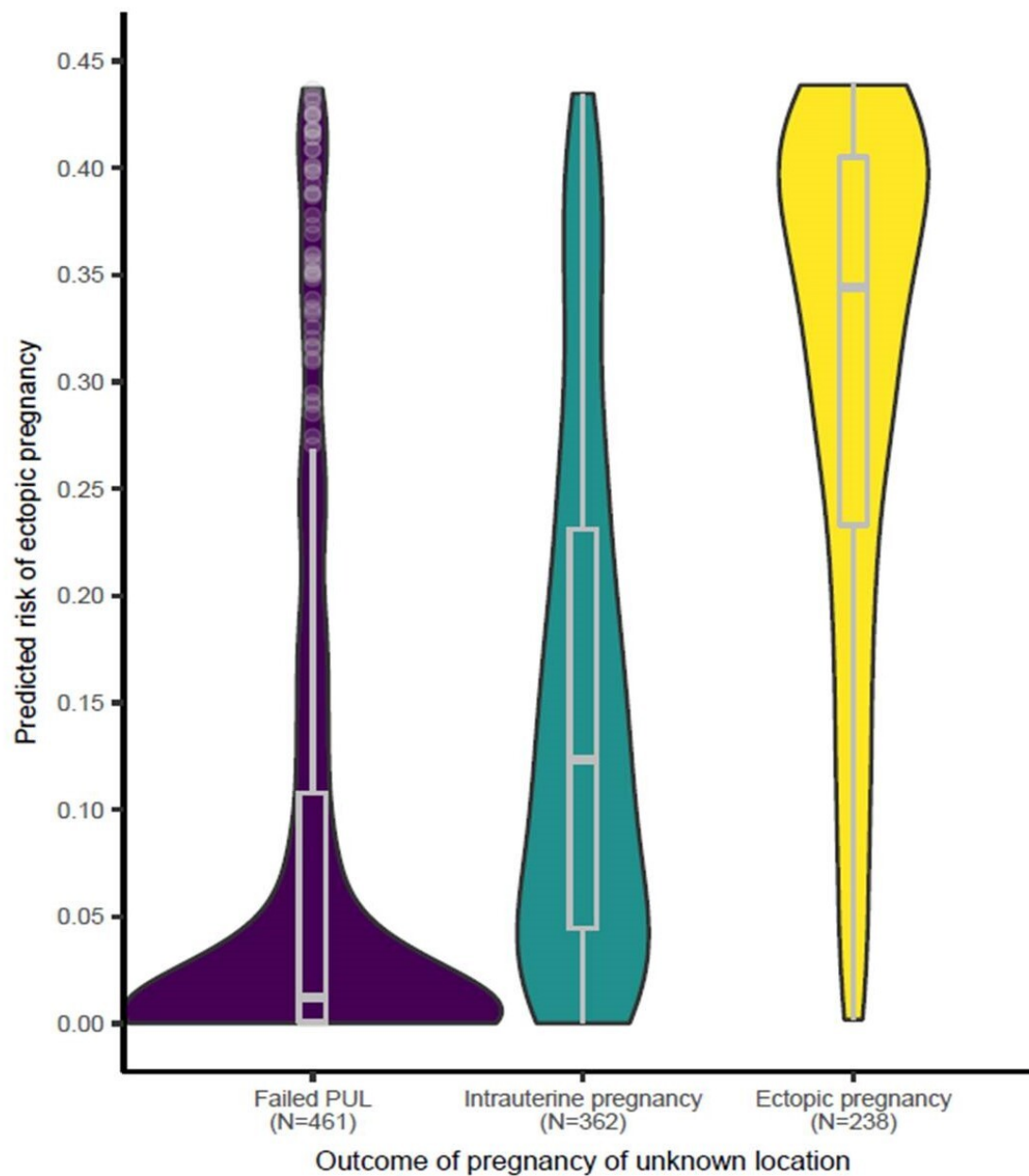

Supplement: Supplementary data [file bmjopen-2021-058454supp001.pdf]

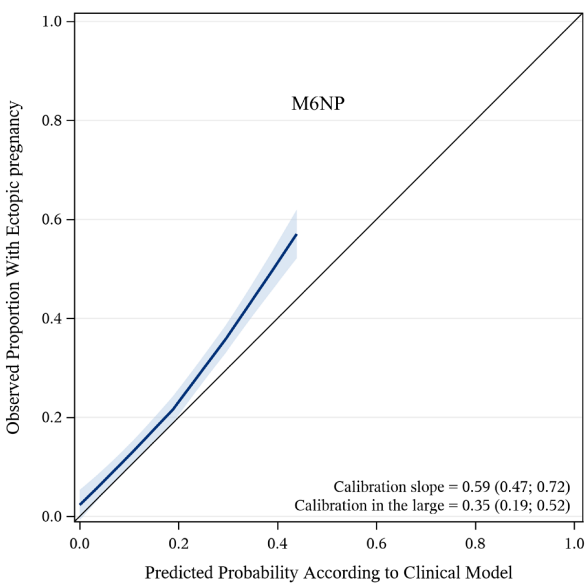

Supplement: Supplementary data [file bmjopen-2021-058454supp002.pdf]

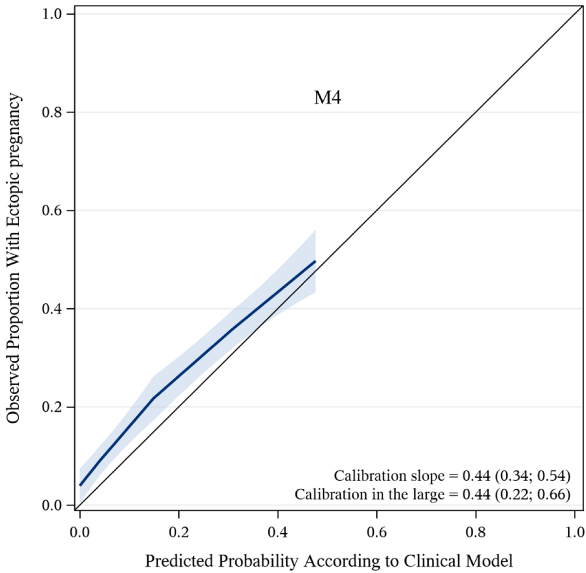

Supplement: Supplementary data [file bmjopen-2021-058454supp003.pdf]
