# Supplementary material for: Pregnancy of unknown location: external validation of the hCG-based M6NP and M4 prediction models in an emergency gynaecology unit
Source: BMJ Open. 2022 Nov 29;12(11):e058454. doi: 10.1136/bmjopen-2021-058454 (PMC9716941; doi:10.1136/bmjopen-2021-058454)
Supplement: Author's manuscript [file bmjopen-2021-058454.draft_revisions.pdf]

BMJ Open is committed to open peer review. As part of this commitment we make the peer review history of every article we publish publicly available.

When an article is published we post the peer reviewers' comments and the authors' responses online. We also post the versions of the paper that were used during peer review. These are the versions that the peer review comments apply to.

The versions of the paper that follow are the versions that were submitted during the peer review process. They are not the versions of record or the final published versions. They should not be cited or distributed as the published version of this manuscript.

BMJ Open is an open access journal and the full, final, typeset and author-corrected version of record of the manuscript is available on our site with no access controls, subscription charges or pay-per-view fees (http://bmjopen.bmj.com).

If you have any questions on BMJ Open's open peer review process please email info.bmjopen@bmj.com

BMJ Open

Pregnancy of unknown location: external validation of the hCG based M6 and M4 prediction models in an emergency gynaecology unit.

Journal:	*BMJ Open*
Manuscript ID	bmjopen-2021-058454
Article Type:	Original research
Date Submitted by the Author:	20-Oct-2021

Complete List of Authors:	Fistouris, Johan; University of Gothenburg Institute of Clinical Sciences, Obstetrics and Gynaecology<br>Bergh, Christina; University of Gothenburg Institute of Clinical Sciences, Obstetrics and Gynaecology<br>Strandell, Annika; University of Gothenburg Institute of Clinical Sciences

Keywords:	ULTRASONOGRAPHY, GYNAECOLOGY, Ultrasound < RADIOLOGY & IMAGING

SCHOLARONE™
Manuscripts

I, the Submitting Author has the right to grant and does grant on behalf of all authors of the Work (as defined in the below author licence), an exclusive licence and/or a non-exclusive licence for contributions from authors who are: i) UK Crown employees; ii) where BMJ has agreed a CC-BY licence shall apply, and/or iii) in accordance with the terms applicable for US Federal Government officers or employees acting as part of their official duties; on a worldwide, perpetual, irrevocable, royalty-free basis to BMJ Publishing Group Ltd ("BMJ") its licensees and where the relevant Journal is co-owned by BMJ to the co-owners of the Journal, to publish the Work in this journal and any other BMJ products and to exploit all rights, as set out in our licence.

The Submitting Author accepts and understands that any supply made under these terms is made by BMJ to the Submitting Author unless you are acting as an employee on behalf of your employer or a postgraduate student of an affiliated institution which is paying any applicable article publishing charge ("APC") for Open Access articles. Where the Submitting Author wishes to make the Work available on an Open Access basis (and intends to pay the relevant APC), the terms of reuse of such Open Access shall be governed by a Creative Commons licence – details of these licences and which Creative Commons licence will apply to this Work are set out in our licence referred to above.

Other than as permitted in any relevant BMJ Author's Self Archiving Policies, I confirm this Work has not been accepted for publication elsewhere, is not being considered for publication elsewhere and does not duplicate material already published. I confirm all authors consent to publication of this Work and authorise the granting of this licence.

For peer review only

Title: Pregnancy of unknown location: external validation of the hCG based M4 and M6 prediction models in an emergency gynaecology unit.

Author names and affiliations: Johan Fistouris[a], Christina Bergh[a] and Annika Strandell[a]

[a]University of Gothenburg Institute of Clinical Sciences, Department of Obstetrics and Gynaecology, Sahlgrenska University Hospital, Gothenburg, Sweden

Correspondence to: Dr Johan Fistouris, Department of Gynaecology and Reproductive Medicine, Sahlgrenska University Hospital, SE-413 45 Gothenburg, Sweden. (E-mail: johan.fistouris@vgregion.se). Telephone: 0046-31-707552678

Keywords: Ectopic pregnancy, Chorionic Gonadotropin, Statistical Models

Word count: 3973

For peer review only

ABSTRACT

Objective: To investigate if the M6 model (M6) predicting ectopic pregnancy among women with pregnancy of unknown location is valid in an emergency gynaecology setting and comparing it with its predecessor M4.

Design: External validation study.

Setting: University hospital

Participants: Women with a pregnancy of unknown location.

Main Outcome Measures: The c-statistic determined the model's ability to differentiate between ectopic and non-ectopic pregnancy. Calibration analysed agreement between predicted risks and observed rate of ectopic pregnancy. The proportion of ectopic and non-ectopic pregnancies classified as high risk assessed the model's sensitivity and false positive rate (FPR). The proportion of non-ectopic pregnancies among women classified as low risk of having an ectopic pregnancy was the models negative predictive value (NPV). Clinical utility was evaluated with the concept of net benefit and decision curve analysis.

Results: 1061 women with at least two serum human chorionic gonadotropin measurements were included, of which 232 (22%) had a final diagnosis of ectopic pregnancy. The c-statistic for M6 was 0.85 (95% CI: 0.82–0.88). M6 as opposed to M4 made accurate risk predictions in the range 0% to 20%. The sensitivity was 95% (95% CI: 92–98), FPR 50% (95% CI: 47–54) and NPV 97% (95% CI: 96–99) when the high risk classification threshold was set at 5% (default). M6 had better c-statistic, sensitivity and NPV than M4 ($p<0.001$). When classification threshold was set at 10% sensitivity was 91% (95% CI: 87–94) and FPR 39% (95% CI: 36–43). M6 had a higher net benefit across all risk thresholds compared with M4.

For peer review only

Conclusions: M6 is valid in women with pregnancy of unknown location attending an emergency gynaecology unit. Sensitivity remains high and FPR is lower when a 10% threshold is used. Our results can encourage implementation of M6 in related yet untested clinical settings to effectively support clinical decision making.

Strengths and limitations of this study

- This is the first study to test the transportability (generalisability) of the M6 prediction model to an emergency gynaecology setting, originally developed for early pregnancy units in the UK.
- The rate of ectopic pregnancy was twice as high in comparison to the original M6 cohort contributing to the different case mix seen in our validation cohort.
- A limitation was the complete case analysis with a risk for introducing selection bias; however a sensitivity analysis showed consistent predictive performance and classification accuracy.
- Another limitation was the retrospective inclusion of women, although common for validation studies.

For peer review only

INTRODUCTION

To reduce maternal complications from ectopic pregnancy (EP), an early diagnosis using transvaginal ultrasound (TVUS) is fundamental [1]. Approximately 30 to 40 % of EP remain undiagnosed after the first examination of women presenting with pain or bleeding in early pregnancy and will be managed as a pregnancy of unknown location (PUL) until a final diagnosis is established [2]. The rupture rate of an EP among these women range from 0.03% to 10% and sometimes necessitates blood transfusion [3-6]. A key concept during follow up is timely diagnosis and treatment of an EP while avoiding unnecessary visits for the remaining majority of women that eventually will be diagnosed with a intrauterin pregnancy (IUP) or a spontaneously resolving PUL (failed PUL) [7]. The change in serum human chorionic gonadotropin (hCG) levels between two samples measured 48 hours apart indicates if a woman is at high or low risk of having an EP and decisive of planning follow- up visits [8]. In a recent meta-analysis it was concluded that the widely reported logistic regression model "M4" that is based on two hCG values, was the best available method to predict the presence of an EP [9-12].

M4 was developed in an dedicated early pregnancy assessment unit (EPAU) in London and has mostly been evaluated in similar clinical settings within that geographical region [10]. When tested in a US population its ability to distinguish between EP and non-EP was clearly useful as the area under the receiver operating characteristic (ROC) curve was 0.8 but classification accuracy was poor with a sensitivity of only 55% [13]. The M4 model has been updated to the M6 model (M6) in a larger cohort of women with a PUL [14]. According to recent research M6 to date has the best predictive performance when applied on women in EPAU [15]. M6 has not been validated in an emergency gynaecology unit with a somewhat

For peer review only

different clinical context and case mix compared with the original M6 cohort which would be a stronger test of its generalizability [16].

The primary aim of the present study was to externally validate M6 in an emergency gynaecology unit and compare the performance to its predecessor M4. We also wanted to assess the clinical utility by performing decision curve analysis (DCA) and evaluate different risk prediction thresholds [17].

MATERIALS AND METHODS

Design and Setting

External validation was performed on women with PUL in the emergency gynaecology unit at the Sahlgrenska University Hospital from January 1st, 2011 to December 31st, 2013.

The emergency gynaecology unit is open 24 hours a day, 7 days a week and serves patients with various gynaecological problems either self-referred or referred from other clinics in the greater Gothenburg area. The study hospital was the only referral site for the treatment of EP in the area as opposed to other early pregnancy complications. Initial examination of women with early pregnancy complaints was performed by a physician in early stage of specialist training and a consultant gynaecologist assisted upon request. Serial hCG measurments was applied whenever the physician was uncertain about the location of the pregnancy based on TVUS and clinical findings.

The second blood sample for hCG measurement were routinely scheduled two to three days after the first hCG. Interpretation of hCG development was not based on any specific percentage change but approximations of the doubling time in hCG for a viable IUP and the expected hCG decline in miscarriage/failed PUL [18]. Follow-up visits were arranged

For peer review only

according to hCG development. Outpatients with deteriorating symptoms were instructed to re- visit the emergency gynaecology unit and depending on the severity of symptoms and clinical findings, inpatient evaluation and further investigation by a senior physician were considered.

Laparoscopic intervention was the first line of treatment for EP during the study period. A diagnostic laparoscopy was also performed in patients with a prolonged period of follow-up where hCG levels did not decline and there was no evidence of an IUP. In cases where laparoscopy failed to identify an EP uterine curettage followed to establish a final diagnosis. Methotrexate was used if there was persistent trophoblast after initial surgical treatment or as treatment of interstitial, cervical or caesarean scar pregnancies.

Participants

Women presenting to our emergency gynaecology unit with complaints in early pregnancy, deemed to have a PUL were screened for inclusion. All women with at least two serial hCG values where the second hCG was measured 1 to 3 days after the first hCG were eligible for inclusion. Also, women that were initially managed as outpatients but the second hCG was measured during or after a hospital stay were included unlike prior studies. We excluded women that had a second hCG sample taken outside the interval of 1 to 3 days as in the original M6 study [14] and if the final outcome of PUL was unknown (lost to follow-up).

Data Collection

The following variables were obtained from the patients electronical medical record: Age at the first visit; symptoms related to the first visit (i.e. pain or bleeding); prior EP or use of a

For peer review only

contraceptive intrauterine device; value of the first and second serum hCG (IU/L); date and time of hCG measurments; the type of PUL and the final outcome of the pregnancy.

We defined three types of a PUL based on TVUS at the first visit or during follow-up [19]: no signs of a pregnancy neither inside nor outside of the uterus defined a true PUL; when an intrauterine sac-like structure or suspicion of retained products of conception was seen it was defined as a probable IUP; probable EP was used when an inhomogeneous adnexal mass or extrauterine (i.e. tubal, cervical, caesarean scar, interstitial, corneal, ovarian, abdominal and heterotopic) sac-like structure was seen. If there was a gestational sac containing a yolk sac with or without a foetus a definite intra-or extrauterine pregnancy was defined and therefore not a PUL.

Reference Standard

Three categories of the final outcome of a PUL were used as reference standard. A failed PUL was defined by spontaneously declining hCG levels to < 0.6 IU/L without establishing a location. An IUP was diagnosed by either TVUS; histological findings of chorionic villi after uterine evacuation; completion of a suspected miscarriage after medical or surgical treatment verified by TVUS or an eventful decline of hCG to <0.6 IU/L. An EP was diagnosed at laparoscopy (tubal, interstitial or ovarian) or hysteroscopy (cervical or caesarean scar pregnancy) with or without confirmed histology or by TVUS.

A persistent PUL (PPUL) was defined by a non-confirmed location of the pregnancy neither by laparoscopy, nor without evidence of chorionic villi after uterine evacuation and hCG levels failed to decline. Cases where hCG levels changed less than 15% during a one-week follow-up or a non-visualized pregnancy was treated with Methotrexate were also categorized as PPUL. In the analysis PPUL were incorporated among EP.

For peer review only

We used the same definitions as in the original M4 and M6 studies and a consensus statement [10, 14, 19].

Prediction models

Both models use polynomial logistic regression to compute risks (%) of each of the three possible outcomes of PUL: failed PUL, IUP and EP. The covariates of the models are based on two hCG (IU/L) measured in serum approximately two days apart (Supplemental Table 1).

By default both models classify a patient as high risk if the computed risk of EP is ≥ 5%. If a patient is classified as low risk either a failed PUL or an IUP is predicted depending on which that has the highest computed risk. We used the original equations (Supplemental Material) of M6 and M4 to compute risks as recommended instead of refitting the model [16, 20].

The TRIPOD (Transparent Reporting of a multivariable for individual Prognosis or Diagnosis) guidelines were followed conducting this study [21].

Study Size

No sample size calculation was performed. A validation cohort should contain at least 100 events and ideally 200 events to adequately evaluate prediction rules [22-24]. Our validation cohort contained 238 EP satisfying this requirement.

Patient and public involvement statement

Patients and the public were not involved in the development of the research question.

For peer review only

Statistical Analysis

The predictive performance of the models was assessed by discrimination and calibration [20]. We focused on the dichotomous outcome of EP and non-EP as being the most important to distinguish in clinical practice. Discrimination was quantified with the c-statistic that is equal to the area under the ROC curve. The c-statistic compared the predicted risk of all possible pairs of patients and if the model always yielded a higher risk to a patient with an EP than to a patient without, the c-statistic would be 1.0. If the c-statistic is 0.5 the model performs no better than chance.

The reliability of the predicted risk of EP in the whole population and at different risk thresholds was assessed with a calibration curve [25, 26]. The calibration curve shows the agreement between the predicted risk (%) of EP on the x-axis and the observed rate of EP on the y-axis. If the intercept of the curve is above 1 the model systematically underestimates the observed rate of EP and overestimates if the intercept is below 1. If the slope of the curve is below 1 risk predictions in the higher end are overestimated. Risk predictions in the lower end are underestimated and vice versa if the slope of the curve is above 1 [24].

In a clinical context the performance of a prediction model depends on how well it support clinical decision making which can be assessed by its classification accuracy. Classification accuracy was defined by the models sensitivity (proportion of EP classified as high risk), false positive rate (FPR), (proportion of non-EP classified as high risk) and the negative predictive value (proportion of non-EP among women classified as low risk) as it was presented in the original M6 study [14]. The pre-specified threshold of 5% to classify a woman as high risk of having an EP is partly arbitrary [15, 27]. Since an optimal threshold may vary between

For peer review only

different populations we explored other thresholds to find a cut-off with the highest clinical value balancing sensitivity and FPR [28].

As a final step we evaluated the clinical utility of the model by calculating net benefit (NB) (Supplemental Material) [29]. This is a single overall measure of classification that incorporates the clinical notion that a correctly classified EP is valued higher than a correctly classified non-EP. The value of NB ranges from negative infinity to the observed rate of EP in the cohort.. At any given threshold used for high risk classification, the model with the higher NB is the better model. The NB was plotted across a range of thresholds in a decision curve [17]. In the decision curve a line representing a default strategy classifying all women at high risk of EP regardless of risk prediction is set as reference. If a curve is completely above this line the model has a NB across the entire range of thresholds. If under this line the model has no NB.

We used a complete case analysis for the validation cohort why a sensitivity analysis was performed to test if the exclusion of women missing a second hCG sampling within the range of 1 to 3 days presented a risk for introducing bias [30]. In the sensitivity analysis all women regardless of hCG sampling interval were included.

Performance measures are presented with 95% confidence intervals (CI). Confidence intervals were obtained using 2000 bootstrap samples. The McNemar´s test was used for testing differences of paired samples and a p-value of < 0.05 was considered statistically significant. The val.prob.ci.2 function was used to generate flexible calibration curves based on LOWESS (Locally Weighted Scatterplot Smoothing) and related statistics [24].

For peer review only

214 Statistical analyses were performed using SPSS 21.0 (SPSS, Chicago, IL, USA) and R 4.0.4 (R foundation for statistical computing, Vienna, Austria). We used the package rmda 1.6 (risk model decision analysis) to calculate NB and generate decision curves.

RESULTS

A total of 1202 women with PUL were reviewed for inclusion. The flowchart (Fig. 1) shows the inclusion procedure of 1061 women in the validation cohort. Descriptive statistics including hCG data for all women and the validation cohort are presented in Table 1. In the validation cohort 71% of women had an hCG sampling interval of 2 days and 89 (8%) were managed as inpatients before the second hCG value was apprehended. Final outcomes of PUL were 461 failed PUL, 362 IUP and 238 EP (22%) of which 46 were PPUL (Supplemental Table 2).

Supplemental Table 2 includes data from the original M4 and the M6 studies where the rate of EP was 6% and 11% respectively. The median of the first hCG value for failed PUL was 334 IU/L in the validation cohort compared with 308 IU/L in the original M6 cohort. The average value of the first and second hCG among failed PUL was 261 IU/L in the validation cohort and 89 IU/L in the original M4 cohort. Intrauterine pregnancies in the validation cohort had three times as high median of the first hCG value as IUP in the original M6 cohort. The hCG ratio (second hCG value/first hCG value) was 1.81 for IUP in the validation cohort as compared with 2.12 and 2.15 in the original M4 and M6 cohorts. In the validation cohort the median of the first hCG value for EP was of 810 IU/L and the ratio was 1.05 as compared with 521 IU/L and 1.16 in the original M6 cohort.

For peer review only

Discrimination and Calibration

The c-statistic for M4 was 0.81 (95% CI: 0.78–0.84) and 0.85 (95% CI: 0.82–0.88) for M6 (p<0.001). As seen in the calibration curve the intercept for M4 was 0.77 and M6 had an intercept of 0.59 (Fig. 2). This means that the risk prediction of EP on average where underestimated by both models. The slope of the curves was in opposite directions for M4 (<1) and M6 (>1) indicating miscalibration in different risk ranges. M4 was poor calibrated through all risk estimates with the calibration curve well above the reference line. M6 made accurate risk predictions for women in the range 0% to 20% but underestimated risks in women at higher risk (Fig. 2).

Classification and Clinical Utility

At the 5% risk threshold M4 classified 85% (95% CI: 81–90) of EP as high risk while the sensitivity for M6 was 95% (95% CI: 92–98) (Table 2). The FPR was 37% (95% CI: 34–40) and 50% (95% CI: 47–54) for M4 and M6 respectively. The negative predictive value (NPV) for M4 was 94% (95% CI: 92–96) and 97% (95% CI: 96–99) for M6. M4 and M6 classified 52% (95% CI: 50–55) and 40% (95% CI: 37–42) of women as low risk respectively. Less than 50% of failed PUL rendered 5% risk predictions of an EP by M6 and 75% of IUP (Supplemental Figure 1c). This equals the proportion of failed PUL and IUP being misclassified as EP by M6.

At the 10% threshold the sensitivity for M6 was 91% (95% CI: 87–94), the FPR was 39% (95% CI: 36–43) and NPV 96% (95% CI: 94–98), (Table 2). The sensitivity was lower at 80% (95% CI: 75–87) for M4 while the FPR was improved but not as marked as for M6 (Table 2).

Decision curve analysis showed that M6 dominated the entire curve in comparison to M4 (Fig. 3). This means that M6 had the best clinical utility (highest NB) across the entire range

For peer review only

of risk thresholds. M4 had no NB at risk thresholds below 7% and the curve was under the reference line (classify all women as high risk) at the 5% risk threshold.

Sensitivity Analysis

Descriptive statistics for all 1186 women were similar to those in the validation cohort except the hCG sampling interval (Table 2). The c-statistic of both models was marginally lower (Supplemental Table 3). The overestimation of non-EP was slightly increased for both models as seen in the calibration plot (Supplemental Fig. 2). M6 made accurate risk predictions up to the 20% risk prediction range. The sensitivity and the FPR at the 5% risk threshold were almost the same as in the validation cohort for both models, although slightly lower (Supplemental Table 3).

DISCUSSION

This external validation study found that M6 perform similar in a Swedish emergency gynaecology unit as in EPAU in the UK where it was developed. Risk predictions made by M6 were accurate at thresholds relevant for clinical decision making (classification). Underestimation of risk predictions among patients with higher risk estimates is thus irrelevant in this respect [24]. We also found that M6 was superior to M4 in every aspect of performance.

Our validation cohort had a twice as high rate of EP and lower rate of failed PUL in comparison to the original M4 and M6 cohorts which contributed to a different case mix [14, 15]. As in previous studies M6 mainly misclassified IUP and to a lesser extent failed PUL. Failed PUL in our cohort generated higher risk estimates of an EP, compared to failed PUL in a validation study by Christodoulou et al. (2021). A greater number of failed PUL were thus

For peer review only

misclassified as high risk at the 5% threshold adding to a higher FPR in our cohort [14, 15]. The reasons for discrepancies between risk estimates could be because of differences in sampling time between two hCG measurements, inclusion of inpatients and that the median hCG values in our study were generally higher compared to prior studies. Combining these differences ultimately led to a stronger test of the generalizability of M6.

One limitation of the study was the exclusion of 141 women and the complete case analysis approach. However a sensitivity analysis that included all women revealed that predictive performance and classification accuracy were sparsely affected. We have no reason to believe that validation cohort is not representative of the target population and the sample size after exclusion was still large enough for inference about the models' predictive performance [22]. A second limitation is the retrospective design of the study although this is a common design when validating or develop prediction models [16] as in the original M6 study.

In clinical practice women with a predicted failed PUL after just two hCG measurments are followed up with a home urine pregnancy test after two weeks and if an IUP is predicted examination is repeated after one week. Women classified at high risk of having an EP are re-examined within 24 hours [3, 31]. In such clinical situation high classification accuracy is crucial for a prediction model to be useful. In our study M6 only misclassified 5% (n=12) of EP as low risk of which eight were predicted to have a failed PUL. On the other hand, 50% of non-EP were misclassified as high risk which could result in high numbers of unnecessary visits, admissions to the ward and potentially interventions if implemented. There is insufficient data to rely on when it comes to costs and benefits of misclassifying women with a PUL which makes it difficult to define an optimal threshold, a general challenge when

For peer review only

implementing a prediction model in clinical practice [28]. No formal decision analysis [32] has ever been performed instead an optimal classification threshold for M6 and M4 has been based on the theory of NB and DCA [29, 33-35] . It has been considered reasonable to allow 19 misclassified non-EP for every correctly classified EP which equals the 5% risk threshold [15]. Our population had a high rate of EP and the 5% threshold therefore seems justifiable to optimize sensitivity. At the 10% threshold M6 would still only misclassify 9% of EP which is below the rate of 16.8% that was achieved when the expected rate of increase and decline in hCG levels for miscarriage and IUP were used for prediction and considered satisfactory in a US study [5, 36, 37]. Since M6 had NB at both the 5 and 10% thresholds in our study any of these could be appropriate.

Regardless which threshold that is finally selected one must remember that M6 should always be used as a compliment to clinical judgement and never as a sole decision instrument. M6 is available at www.earlypregnancycare.com/m6 where the 5% threshold is used by default.

CONCLUSION

The performance of M6 as opposed to M4 was encouraging when validated on women with PUL in an emergency gynaecology unit. M6 can be introduced in related yet untested clinical settings outside the UK and EPAU to effectively support clinical decision making. Recalibration of M6 in a national context to account for geographical differences could be an option to improve its performance.

Contributors J.F. and A.S. contributed substantially to conception and design of the study. J.F collected the data. J.F performed the statistical analysis and J.F and A.S interpreted the data.

For peer review only

J.F. and A.S. wrote the article. J.F drafted the article. C.B. commented on the drafts. All authors made revisions to the article and approved the final version.

Funding This work was supported by grants from the Swedish state under the agreement between the Swedish government and the county councils, the ALF-agreement [ALFGBG-720291, ALFGBG-72150].

Competing interests None declared.

Ethics approval statement approval for this study was obtained from the Regional Ethical Review Board, Gothenburg, Sweden (approval # 50115).

Data availability statement Data are available on reasonable request. Requests should be made to the lead author johan.fistouris@vgregion.se.

For peer review only

REFERENCES

1. RCOG/AEPU. Diagnosis and Management of Ectopic Pregnancy: Green-top Guideline No. 21. BJOG 2016; 123: e15-e55.
2. Crochet J BL. Does ThisWoman Have an Ectopic Pregnancy? The Rational Clinical Examination Systematic Review. JAMA 2013; Vol 309: 1722-29.
3. Bobdiwala S, Christodoulou E, Farren J, Mitchell-Jones N, Kyriacou C, Al-Memar M, et al. Triaging women with pregnancy of unknown location using two-step protocol including M6 model: clinical implementation study. Ultrasound Obstet Gynecol 2020; 55: 105-14.
4. Nadim B, Leonardi M, Infante F, Lattouf I, Reid S ,Condous G. Rationalizing the management of pregnancies of unknown location: Diagnostic accuracy of human chorionic gonadotropin ratio-based decision tree compared with the risk prediction model M4. Acta Obstet Gynecol Scand 2020; 99: 381-90.
5. Morse CB, Sammel MD, Shaunik A, Allen-Taylor L, Oberfoell NL, Takacs P, et al. Performance of human chorionic gonadotropin curves in women at risk for ectopic pregnancy: exceptions to the rules. Fertil Steril 2012; 97: 101-6 e2.
6. Fistouris J, Bergh C ,Strandell A. Classification of pregnancies of unknown location according to four different hCG-based protocols. Hum Reprod 2016; 31: 2203-11.
7. Kirk E, Bottomley C ,Bourne T. Diagnosing ectopic pregnancy and current concepts in the management of pregnancy of unknown location. Hum Reprod Update 2014; 20: 250-61.
8. van Mello NM, Mol F, Opmeer BC, Ankum WM, Barnhart K, Coomarasamy A, et al. Diagnostic value of serum hCG on the outcome of pregnancy of unknown location: a systematic review and meta-analysis. Hum Reprod Update 2012; 18: 603-17.
9. Bobdiwala S, Saso S, Verbakel JY, Al-Memar M, Van Calster B, Timmerman D, et al. Diagnostic protocols for the management of pregnancy of unknown location: a systematic review and meta-analysis. BJOG 2019; 126: 190-98.
10. Condous G, Van Calster B, Kirk E, Haider Z, Timmerman D, Van Huffel S, et al. Prediction of ectopic pregnancy in women with a pregnancy of unknown location. Ultrasound Obstet Gynecol 2007; 29: 680-7.
11. Guha S, Ayim F, Ludlow J, Sayasneh A, Condous G, Kirk E, et al. Triaging pregnancies of unknown location: the performance of protocols based on single serum progesterone or repeated serum hCG levels. Hum Reprod 2014; 29: 938-45.
12. Bobdiwala S, Guha S, Van Calster B, Ayim F, Mitchell-Jones N, Al-Memar M, et al. The clinical performance of the M4 decision support model to triage women with a pregnancy of unknown location as at low or high risk of complications. Hum Reprod 2016; 31: 1425-35.
13. Barnhart KT, Sammel MD, Appleby D, Rausch M, Molinaro T, Van Calster B, et al. Does a prediction model for pregnancy of unknown location developed in the UK validate on a US population? Hum Reprod 2010; 25: 2434-40.
14. Van Calster B, Bobdiwala S, Guha S, Van Hoorde K, Al-Memar M, Harvey R, et al. Managing pregnancy of unknown location based on initial serum progesterone and serial serum hCG levels: development and validation of a two-step triage protocol. Ultrasound Obstet Gynecol 2016; 48: 642-49.
15. Christodoulou E, Bobdiwala S, Kyriacou C, Farren J, Mitchell-Jones N, Ayim F, et al. External validation of models to predict the outcome of pregnancies of unknown location: a multicentre cohort study. BJOG 2021; 128: 552-62.
16. Moons KG, Kengne AP, Grobbee DE, Royston P, Vergouwe Y, Altman DG, et al. Risk prediction models: II. External validation, model updating, and impact assessment. Heart 2012; 98: 691-8.
17. Vickers AJ ,Elkin EB. Decision curve analysis: a novel method for evaluating prediction models. Med Decis Making 2006; 26: 565-74.

For peer review only

18. Butts SF, Guo W, Cary MS, Chung K, Takacs P, Sammel MD, et al. Predicting the decline in human chorionic gonadotropin in a resolving pregnancy of unknown location. Obstet Gynecol 2013; 122: 337-43.
19. Barnhart K, van Mello NM, Bourne T, Kirk E, Van Calster B, Bottomley C, et al. Pregnancy of unknown location: a consensus statement of nomenclature, definitions, and outcome. Fertil Steril 2011; 95: 857-66.
20. Altman DG, Vergouwe Y, Royston P ,Moons KG. Prognosis and prognostic research: validating a prognostic model. . BMJ 2009; 338: b605.
21. Collins GS, Reitsma JB, Altman DG ,Moons KG. Transparent reporting of a multivariable prediction model for individual prognosis or diagnosis (TRIPOD): the TRIPOD statement. BMJ 2015; 350: g7594.
22. Collins GS, Ogundimu EO ,Altman DG. Sample size considerations for the external validation of a multivariable prognostic model: a resampling study. Stat Med 2016; 35: 214-26.
23. Vergouwe Y, Steyerberg EW, Eijkemans MJ ,Habbema JD. Substantial effective sample sizes were required for external validation studies of predictive logistic regression models. J Clin Epidemiol 2005; 58: 475-83.
24. Van Calster B, Nieboer D, Vergouwe Y, De Cock B, Pencina MJ ,Steyerberg EW. A calibration hierarchy for risk models was defined: from utopia to empirical data. J Clin Epidemiol 2016; 74: 167-76.
25. Alba AC, Agoritsas T, Walsh M, Hanna S, Iorio A, Devereaux PJ, et al. Discrimination and Calibration of Clinical Prediction Models: Users' Guides to the Medical Literature. JAMA 2017; 318: 1377-84.
26. Austin PC ,Steyerberg EW. Graphical assessment of internal and external calibration of logistic regression models by using loess smoothers. Stat Med 2014; 33: 517-35.
27. Van Calster B, Abdallah Y, Guha S, Kirk E, Van Hoorde K, Condous G, et al. Rationalizing the management of pregnancies of unknown location: temporal and external validation of a risk prediction model on 1962 pregnancies. Hum Reprod 2013; 28: 609-16.
28. Steyerberg EW, Vickers AJ, Cook NR, Gerds T, Gonen M, Obuchowski N, et al. Assessing the performance of prediction models: a framework for traditional and novel measures. Epidemiology 2010; 21: 128-38.
29. Fitzgerald M, Saville B ,Lewis R. Decision Curve Analysis. JAMA 2015; 313.
30. Joshi M, Royuela A ,Zamora J. Proper analysis in clinical trials: how to report and adjust for missing outcome data. BJOG 2013; 120: 915-19.
31. NICE guideline [NG126]. https://www.nice.org.uk/guidance/ng126 (Accessed September 14, 2021) 2019.
32. Hunink M, Glasziou P ,Siegel J. Decision Making in Health and Medicine: Integrating Evidence and Values. New York:Cambridge University Press 2014.
33. Vickers AJ, Van Calster B ,Steyerberg EW. Net benefit approaches to the evaluation of prediction models, molecular markers, and diagnostic tests. BMJ 2016; 352: i6.
34. Kerr KF, Brown MD, Zhu K ,Janes H. Assessing the Clinical Impact of Risk Prediction Models With Decision Curves: Guidance for Correct Interpretation and Appropriate Use. J Clin Oncol 2016; 34: 2534-40.
35. Collins GS ,Altman DG. Predicting the 10 year risk of cardiovascular disease in the United Kingdom: independent and external validation of an updated version of QRISK2. BMJ 2012; 344: e4181.
36. Barnhart KT, Sammel MD, Rinaudo PF, Zhou L, Hummel AC ,Guo W. Symptomatic patients with an early viable intrauterine pregnancy: HCG curves redefined. Obstet Gynecol 2004; 104: 50-5.
37. Barnhart K, Sammel MD, Chung K, Zhou L, Hummel AC ,Guo W. Decline of serum human chorionic gonadotropin and spontaneous complete abortion: defining the normal curve. Obstet Gynecol 2004; 104: 975-81.

For peer review only

Figure Legends

Figure 1. Flow chart showing the inclusion of patients in the validation cohort and the sensitivity analysis. PUL, pregnancy of unknown location; hCG, human chorionic gonadotropin.

Figure 2. Calibration plot showing the agreement between the predicted risk of ectopic pregnancy (x axis) by the M4 model (red line) and the M6 model (blue line) and the observed proportion of ectopic pregnancy (y axis). Predictions above the reference line (45°) equals underestimation of the predicted probability of ectopic pregnancy and overestimation if below the reference line.

Figure 3. Decision curve analysis of the M4 (red line) and the M6 (blue line) model. The high risk threshold is above which a woman is classified as high risk of ectopic pregnancy. The green line (set as reference) shows the net benefit of considering all women at high risk of ectopic pregnancy and the black line shows the net benefit of classifying no women as high risk.

For peer review only

**Table 1.** Descriptive statistics of all patients and patients in the validation cohort.		
**Characteristics**	All patients[a] n=1186	Validation cohort n=1061
Age (years)[b]	31 (26; 35) 15; 49	31 (26; 35) 15; 49
First hCG value (IU/L)[b]	697 (210; 2300) 7; 180000	703 (210; 2312) 7; 180000
Second hCG value (IU/L)[b]	615 (142; 2655) 1; 190000	635 (150; 2700) 1; 180000
hCG ratio[b]	0.93 (0.46; 1.57) 0.01; 21.19	0.94 (0.45; 1.59) 0.06; 5.65
Interval between two hCG samples[c]		
<1 day	70 (6)	0 (0)
1 day	135 (11)	141 (13)
2 days	759 (64)	751 (71)
3 days	167 (14)	169 (16)
>3 days	55 (5)	0 (0)
Type of PUL[b]		
True PUL	880 (74)	793 (75)
Probable IUP	254 (21)	239 (22)
Probable EP	52 (4)	29 (3)
Vaginal bleeding[b]	803 (68)	723 (68)
Prior ectopic pregnancy[b]	86 (7)	76 (7)
IUD[b]	37 (3)	30 (3)

PUL = pregnancy of unknown location; IUP = intrauterine pregnancy; EP = ectopic pregnancy; IUD = intrauterine device; Q1 = first quartile; Q3 = third quartile;

[a]Patients that were part of the sensitivity analysis. Patients lost to follow-up (n=16) are not included.

[b]Reported as median (Q1; Q3) and min; max

[c]Reported as n (%)

Because of rounding the summarized percentage sometimes are not exactly 100%

For peer review only

506

507

508

509

510

511

512

Table 2. Classification accuracy of the M4 and the M6 model in the validation cohort at the 5 and 10% thresholds.

Threshold	Classification accuracy[a]	M4	M6	*P*
5%	Sensitivity	85 (81–90)	95 (92–98)	<0.001
	False positive rate	37 (34–40)	50 (47–54)	<0.001
	Negative predictive value	94 (92–96)	97 (96–99)	<0.001
	Percentage classified as low risk	52 (50–55)	40 (37–42)	<0.001
10%	Classification accuracy[a]			
	Sensitivity	80 (75–85)	91 (87–94)	<0.001
	False positive rate	30 (37–34)	39 (36–43)	<0.001
	Negative predictive value	92 (90–94)	96 (94–98)	<0.001
	Percentage classified as low risk	58 (56–61)	49 (46–52)	<0.001

[a]Reported as percentage (95% confidence interval).

513

514

515

516

517

For peer review only

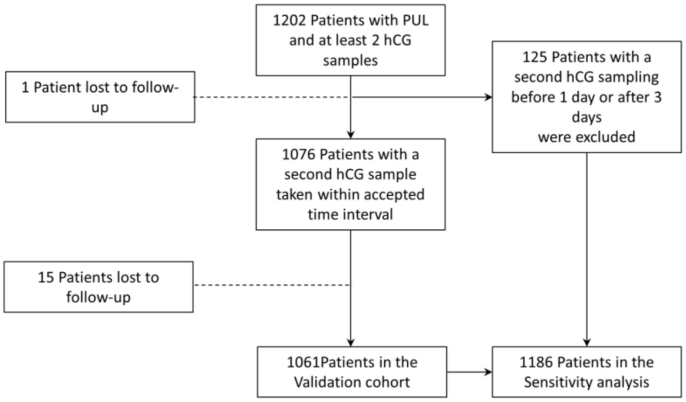

Flow chart showing the inclusion of patients in the validation cohort and the sensitivity analysis. PUL, pregnancy of unknown location; hCG, human chorionic gonadotropin.

77x58mm (600 x 600 DPI)

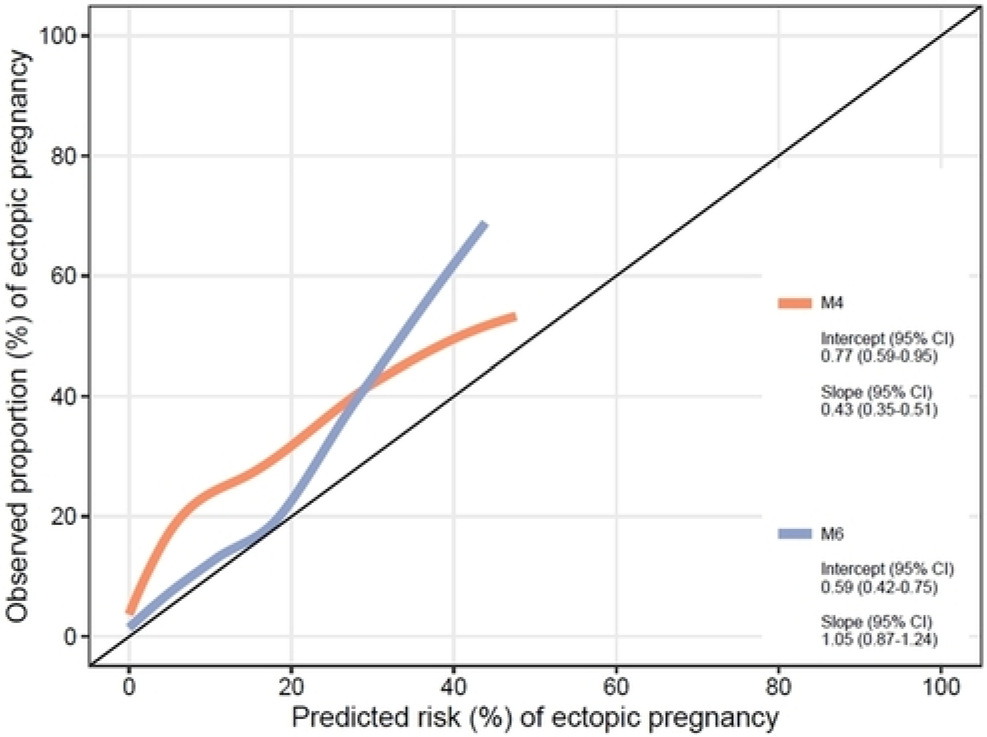

Calibration plot showing the agreement between the predicted risk of ectopic pregnancy (x axis) by the M4 model (red line) and the M6 model (blue line) and the observed proportion of ectopic pregnancy (y axis). Predictions above the reference line (45°) equals underestimation of the predicted probability of ectopic pregnancy and overestimation if below the reference line.

41x31mm (300 x 300 DPI)

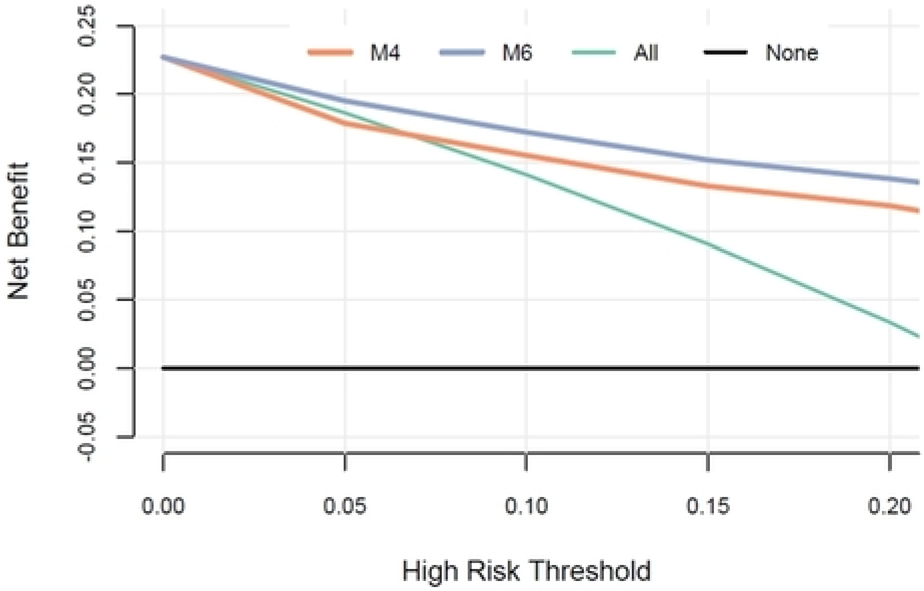

Decision curve analysis of the M4 (red line) and the M6 (blue line) model. The high risk threshold is above which a woman is classified as high risk of ectopic pregnancy. The green line (set as reference) shows the net benefit of considering all women at high risk of ectopic pregnancy and the black line shows the net benefit of classifying no women as high risk.

41x31mm (300 x 300 DPI)

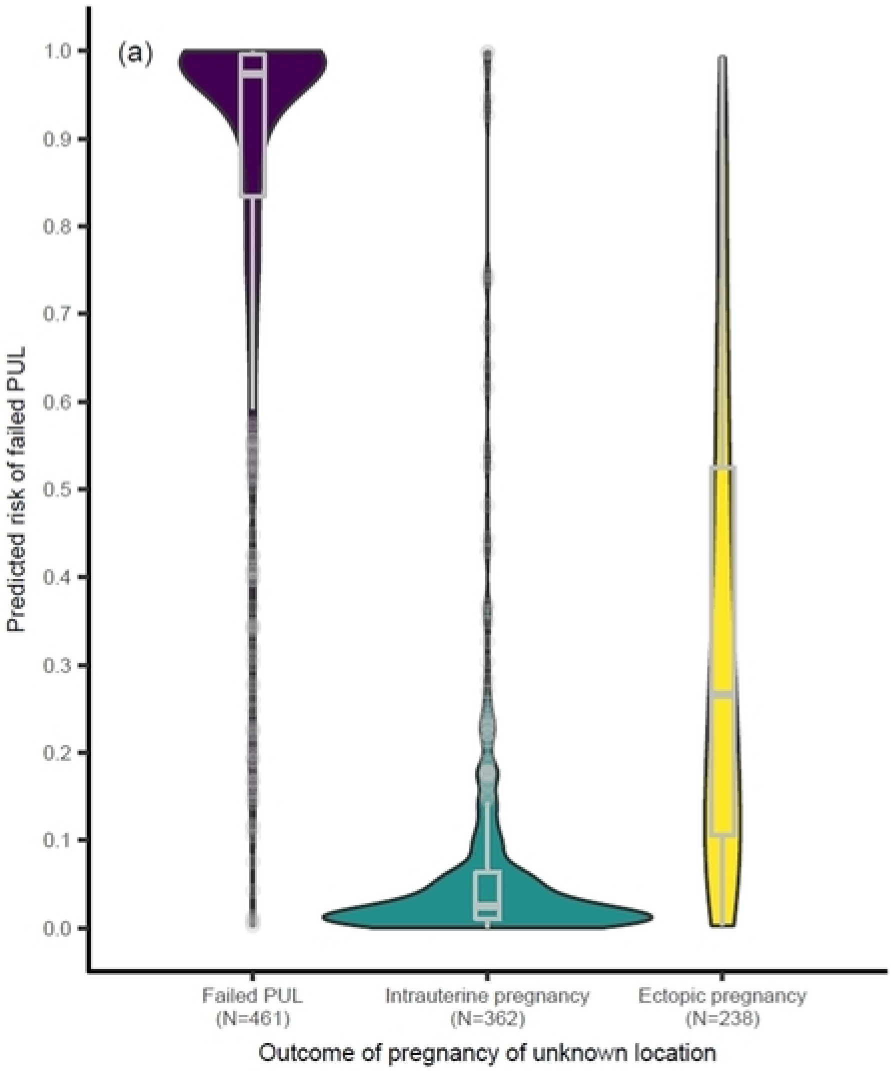

The violin plot shows the full distribution of the predicted risks (%) by the M6 model of (a) failed PUL, (b) intrauterine pregnancy, (c) ectopic pregnancy within each outcome of a pregnancy of unknown location in the validation cohort. The boxes inside the violin plot express the median (Q1;Q3). Plot c indicates that less than 50% of failed PUL rendered 5% risk predictions of an ectopic pregnancy and almost 75% of intrauterine pregnancies. This equals the proportion being misclassified as ectopic pregnancies. PUL, pregnancy of unknown location.

38x46mm (300 x 300 DPI)

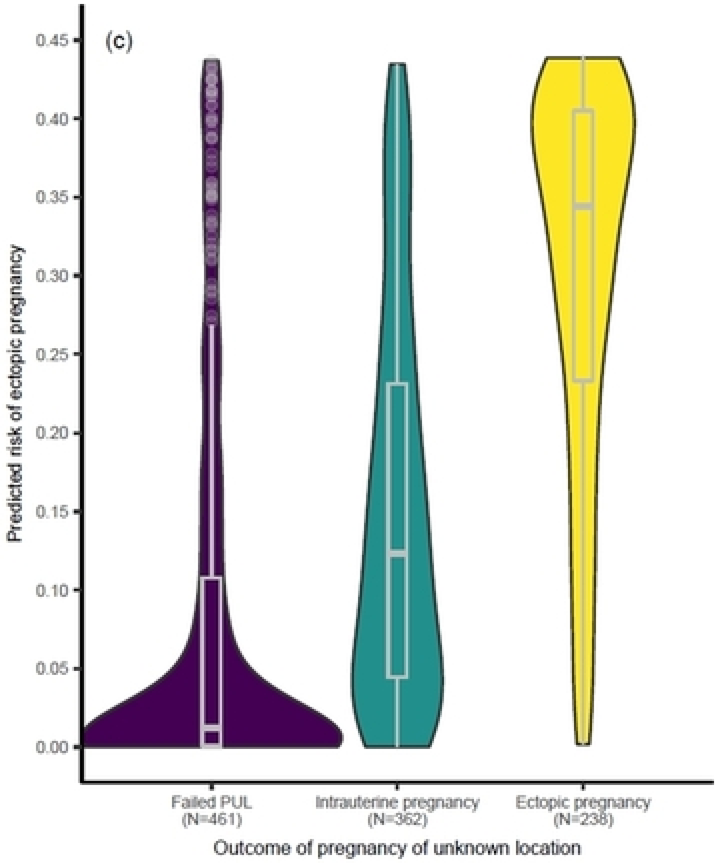

The violin plot shows the full distribution of the predicted risks (%) by the M6 model of (a) failed PUL, (b) intrauterine pregnancy, (c) ectopic pregnancy within each outcome of a pregnancy of unknown location in the validation cohort. The boxes inside the violin plot express the median (Q1;Q3). Plot c indicates that less than 50% of failed PUL rendered 5% risk predictions of an ectopic pregnancy and almost 75% of intrauterine pregnancies. This equals the proportion being misclassified as ectopic pregnancies. PUL, pregnancy of unknown location.

30x37mm (300 x 300 DPI)

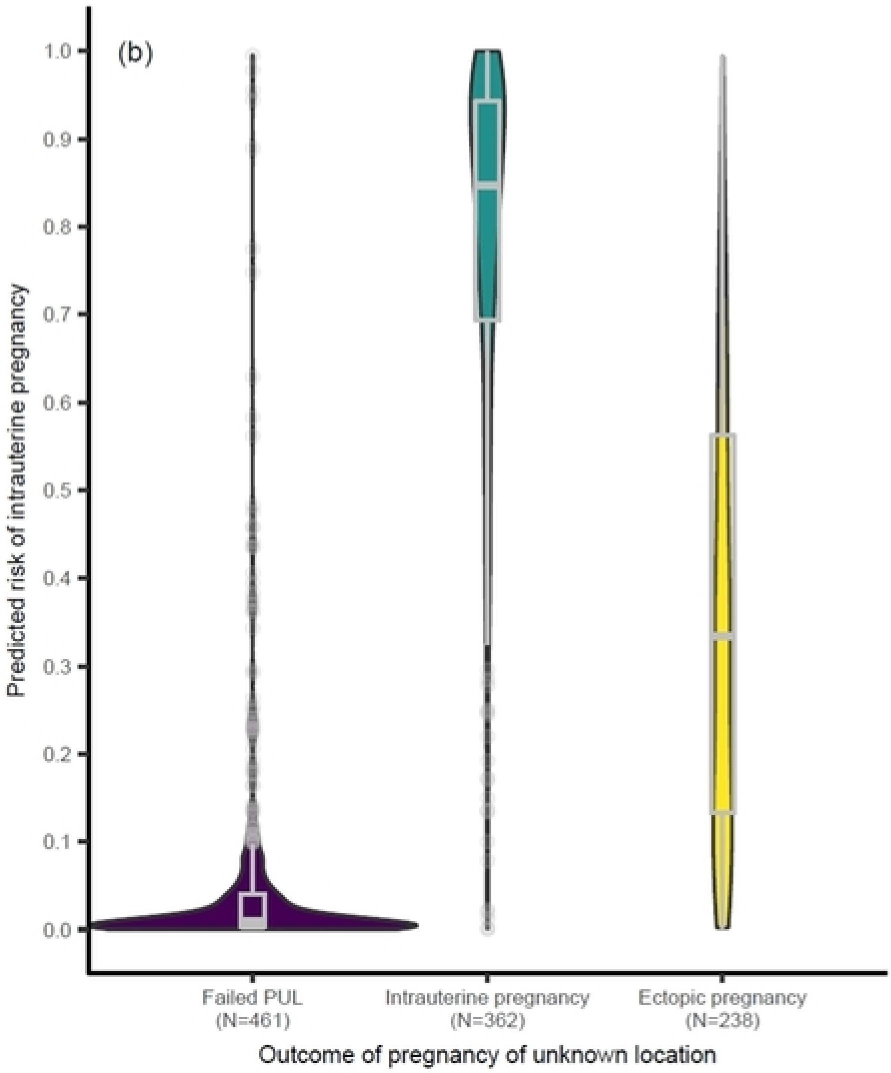

The violin plot shows the full distribution of the predicted risks (%) by the M6 model of (a) failed PUL, (b) intrauterine pregnancy, (c) ectopic pregnancy within each outcome of a pregnancy of unknown location in the validation cohort. The boxes inside the violin plot express the median (Q1;Q3). Plot c indicates that less than 50% of failed PUL rendered 5% risk predictions of an ectopic pregnancy and almost 75% of intrauterine pregnancies. This equals the proportion being misclassified as ectopic pregnancies. PUL, pregnancy of unknown location.

38x46mm (300 x 300 DPI)

For peer review only - http://bmjopen.bmj.com/site/about/guidelines.xhtml

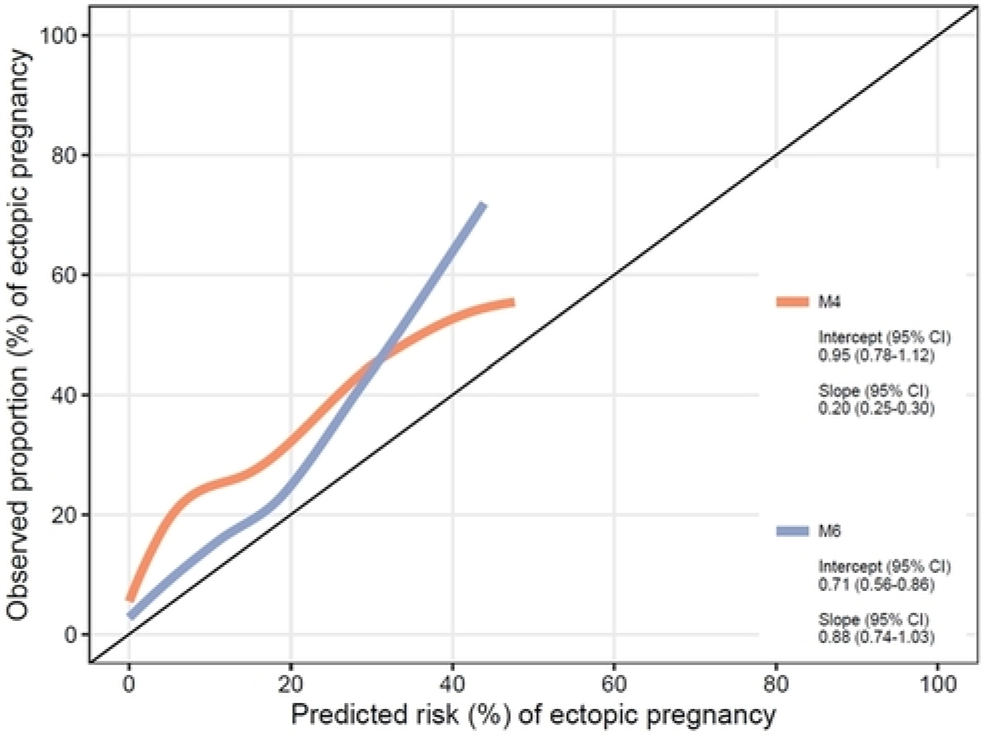

Calibration plot showing the agreement between the predicted risk of ectopic pregnancy (x axis) by the M4 model (red line) and the M6 model (blue line) and the observed proportion of ectopic pregnancy (y axis) in the sensitivity analysis.

41x31mm (300 x 300 DPI)

Supplemental Material

Supplemental Table 1. Covariates used in the original M4[b] and M6[c] cohorts.

Model	Covariate	Description
M4	log(average hCG)	Log transformation of (first hCG + second hCG)/2
M4	centered hCG ratio	Centering transformation of (second hCG /first hCG-1.17[a])
M4	(centered hCG ratio)^2	The quadratic effect was added because the linearity for the logit assumption was violated for hCG ratio.
M6	log(initial hCG)	Log transformation of the first hCG
M6	log(hCG ratio)	Log transformation of (second hCG/first hCG)
M6	[log(hCG ratio)]^2	Square of the log transformed hCG ratio

hCG = human chorionic gonadotropin.

[a]Average hCG ratio in the original M4 cohort. Centering reduced the correlation between hCG ratio and its square from 0.96 to 0.59.

[b]Condous G, Van Calster B, Kirk E, Haider Z, Timmerman D, Van Huffel S, et al. Prediction of ectopic pregnancy in women with a pregnancy of unknown location. Ultrasound Obstet Gynecol 2007; 29: 680-7.

[c]Van Calster B, Bobdiwala S, Guha S, Van Hoorde K, Al-Memar M, Harvey R, et al. Managing pregnancy of unknown location based on initial serum progesterone and serial serum hCG levels: development and validation of a two-step triage protocol. Ultrasound Obstet Gynecol 2016; 48: 642-49.

For peer review only

Supplemental Table 2. Descriptive statistics of failed PUL, intrauterine pregnancies and ectopic pregnancies in the validation cohort and the original M4 [e] and M6 [f] cohorts.

Outcome of PUL/variable	Validation cohort	Original M4 cohort	Original M6 cohort
Failed PUL[a]	457 (43)	109 (55)	785 (54)
Age (Years)[b]	31 (27; 35)	30 (7)	32 (27; 36)
First hCG value (IU/L) [b]	334 (120; 1078)	N/A[c]	308 (104; 975)
Second hCG value (IU/L) [b]	158 (53; 460)	N/A[c]	143 (48; 397)
hCG ratio[b]	0.42 (0.27; 0.64)	0.35 (0.27)	0.40 (0.28; 0.58)
hCG average (IU/L)[b]	261 (93; 790)	89 (216)	N/A[d]
Intrauterine pregnancy[a]	356 (34)	76 (39)	501 (35)
Age (Years)[b]	29 (24; 33)	30 (6)	30 (24; 33)
First hCG value (IU/L) [b]	1521 (464; 5125)	N/A[c]	488 (253; 887)
Second hCG value (IU/L)[b]	2765 (983; 7900)	N/A[c]	1061 (515; 1863)
hCG ratio[b]	1.81 (1.39; 2.21)	2.12 (0.57)	2.15 (1.83; 2.50)
hCG average (IU/L)[b]	2226 (746; 6750)	727 (986)	N/A[d]
Ectopic pregnancy[a]	241 (23)	12 (6)	163 (11)
Age (Years)[b]	32 (27; 36)	32 (6.5)	32 (28; 35)
First hCG value (IU/L)[b]	810 (252; 2315)	N/A[c]	521 (200; 1220)
Second hCG value (IU/L)[b]	920 (265; 2735)	N/A[c]	604 (224; 1266)
hCG ratio[b]	1.05 (0.86; 1.31)	1.41 (0.47)	1.16 (0.96; 1.48)
hCG average (IU/L)[b]	865 (270; 2600)	621 (354)	N/A[d]

PUL = pregnancy of unknown location; Q1 = first quartile; Q3 = third quartile; IQR = interquartile range; N/A = not available.

[a]Reported as number (%)

[b]Reported as median (Q1; Q3) in the validation and the original M6 cohort, and as median (IQR) in the original M4 cohort.

[c]The first and second hCG value were only presented as an average in the original M4 cohort.

[d]The average hCG value was not presented in the original M6 cohort.

[e]Condous G, Van Calster B, Kirk E, Haider Z, Timmerman D, Van Huffel S, et al. Prediction of ectopic pregnancy in women with a pregnancy of unknown location. Ultrasound Obstet Gynecol 2007; 29: 680-7.

[f]Van Calster B, Bobdiwala S, Guha S, Van Hoorde K, Al-Memar M, Harvey R, et al. Managing pregnancy of unknown location based on initial serum progesterone and serial serum hCG levels: development and validation of a two-step triage protocol. Ultrasound Obstet Gynecol 2016; 48: 642-49.

For peer review only

**Supplemental Table 3.** Performance of the M4 and M6 model in the sensitivity analysis.		
Performance measure	M4	M6
C-statistic	0.80 (0.77–0.83)	0.84 (0.81–0.86)
Calibration		
Intercept	0.95 (0.78–1.12)	0.71 (0.56–0.86)
Slope	0.25 (0.20–0.30)	0.88 (0.74–1.03)
Classification accuracy[a]		
Sensitivity	83 (78–87)	93 (90–96)
False positive rate	36 (33–39)	49 (48–53)
Negative predictive value	92 (90–94)	96 (94–98)

[a]Reported as percentage (95% confidence interval).

For peer review only

Linear predictor functions for the M4 model where IUP was reference category [1]:

$$z_1 = log\left(\frac{P(FPUL)}{P(IUP)}\right)$$

$$= +5.8803$$

$$-5.5594 \times (hCG\ ratio - 1.17)$$

$$+2.0504 \times (hCG\ ratio - 1.17)^2$$

$$-1.175 \times \log(average\ hCG)$$

$$z_2 = log\left(\frac{P(EP)}{P(IUP)}\right)$$

$$= 0.3857$$

$$-0.2627 \times (hCG\ ratio - 1.17)$$

$$-3.9319 \times (hCG\ ratio - 1.17)^2$$

$$-0.0572 \times \log(average\ hCG)$$

Linear predictor functions for the M6 model were EP was reference category [2]:

$$z_1 = log\left(\frac{P(FPUL)}{P(EP)}\right)$$

$$= 2.5506$$

$$-0.4242 \times log(initial\ hCG)$$

$$-2.9502 \times log(hCG\ ratio)$$

$$+2.1765 \times [log(hCG\ ratio)]^2$$

$$z_2 = log\left(\frac{P(IUP)}{P(EP)}\right)$$

$$= -3.2842$$

$$+0.4072 \times log(initial\ hCG)$$

$$+1.9238 \times log(hCG\ ratio)$$

$$+2.8952 \times [log(hCG\ ratio)]^2$$

By using the exponentiated linear predictor functions of the M4 and M6 model respectively the probability of each outcome are computed as:

For peer review only

$$P(FPUL) = \frac{exp(z_1)}{1 + exp(z_1) + exp(z_2)}$$

$$P(IUPor\ EP\ ) = \frac{exp(z_2)}{1 + exp(z_1) + exp(z_2)}$$

$$P(EPor\ IUP) = \frac{1}{1 + exp(z_1) + exp(z_2)}$$

The probability of IUP is computed with the third formula when model M4 is used. If the M6 model is used the third formula computes the probability of EP. PUL, pregnancy of unknown location; FPUL, failed PUL; IUP, intrauterin pregnancy, EP, ectopic pregnancy.

Equation of net benefit [3]:

$\text{Net benefit} = (\text{True positive})/n\ -\ (\text{False positive})/n\ \cdot ((P_t/(1 - P_t))$, where true positive is the number of EP classified as high risk; false positive is the number of non-EP classified as high risk and n is the total number of patients. In the equation false positives are weighted by the odds of the selected risk threshold (P_t) for high risk classification. If a 5% risk threshold is chosen, a false positive non-EP is valued at 1/19th of a true positive EP.

References:

1. Condous G, Van Calster B, Kirk E, Haider Z, Timmerman D, Van Huffel S, et al. Prediction of ectopic pregnancy in women with a pregnancy of unknown location. Ultrasound Obstet Gynecol 2007; 29: 680-7.
2. Van Calster B, Bobdiwala S, Guha S, Van Hoorde K, Al-Memar M, Harvey R, et al. Managing pregnancy of unknown location based on initial serum progesterone and serial serum hCG levels: development and validation of a two-step triage protocol. Ultrasound Obstet Gynecol 2016; 48: 642-49.
3. Steyerberg EW, Vickers AJ, Cook NR, Gerds T, Gonen M, Obuchowski N, et al. Assessing the performance of prediction models: a framework for traditional and novel measures. Epidemiology 2010; 21: 128-38.

Figure Legends

Supplemental Figure 1 a-c. The violin plot shows the full distribution of the predicted risks (%) by the M6 model of (a) failed PUL, (b) intrauterine pregnancy, (c) ectopic pregnancy within each outcome of a pregnancy of unknown location in the validation cohort. The boxes inside the violin plot express the median (Q1; Q3). Plot c indicates that less than 50% of failed PUL rendered 5% risk predictions of an ectopic pregnancy and almost 75% of intrauterine pregnancies. This equals the proportion being misclassified as ectopic pregnancies. PUL, pregnancy of unknown location

For peer review only

Supplemental Figure 2. Calibration plot showing the agreement between the predicted risk of ectopic pregnancy (x axis) by the M4 model (red line) and the M6 model (blue line) and the observed proportion of ectopic pregnancy (y axis) in the sensitivity analysis.

For peer review only

Reporting checklist for prediction model development/validation.

Based on the TRIPOD guidelines.

Instructions to authors

Complete this checklist by entering the page numbers from your manuscript where readers will find each of the items listed below.

Your article may not currently address all the items on the checklist. Please modify your text to include the missing information. If you are certain that an item does not apply, please write "n/a" and provide a short explanation.

Upload your completed checklist as an extra file when you submit to a journal.

In your methods section, say that you used the TRIPODreporting guidelines, and cite them as:

Collins GS, Reitsma JB, Altman DG, Moons KG. Transparent reporting of a multivariable prediction model for individual prognosis or diagnosis (TRIPOD): The TRIPOD statement.

		Reporting Item	Page Number
Title			
	#1	Identify the study as developing and / or validating a multivariable prediction model, the target population, and the outcome to be predicted.	1
Abstract			
	#2	Provide a summary of objectives, study design, setting, participants, sample size, predictors, outcome, statistical analysis, results, and conclusions.	1
Introduction			
	#3a	Explain the medical context (including whether diagnostic or prognostic) and rationale for developing or validating the multivariable prediction model, including references to existing models.	3
	#3b	Specify the objectives, including whether the study describes the development or validation of the model or both.	4

For peer review only

Methods			
Source of data	#4a	Describe the study design or source of data (e.g., randomized trial, cohort, or registry data), separately for the development and validation data sets, if applicable.	4
Source of data	#4b	Specify the key study dates, including start of accrual; end of accrual; and, if applicable, end of follow-up.	4
Participants	#5a	Specify key elements of the study setting (e.g., primary care, secondary care, general population) including number and location of centres.	4
Participants	#5b	Describe eligibility criteria for participants.	5
Participants	#5c	Give details of treatments received, if relevant	n/a, no treatment was given
Outcome	#6a	Clearly define the outcome that is predicted by the prediction model, including how and when assessed.	6
Outcome	#6b	Report any actions to blind assessment of the outcome to be predicted.	n/a, predictions and outcome assessment were done separately
Predictors	#7a	Clearly define all predictors used in developing or validating the multivariable prediction model, including how and when they were measured	6 and 7
Predictors	#7b	Report any actions to blind assessment of predictors for the outcome and other predictors.	n/a, predictions and outcome assessment were done separately
Sample size	#8	Explain how the study size was arrived at.	7
Missing data	#9	Describe how missing data were handled (e.g., complete-case analysis, single imputation, multiple imputation) with details of any imputation method.	9
Statistical analysis methods	#10a	If you are developing a prediction model describe how predictors were handled in the analyses.	n/a, validation study
Statistical analysis methods	#10b	If you are developing a prediction model, specify type of model, all model-building procedures (including any predictor selection), and method for internal validation.	n/a

For peer review only

Statistical analysis methods	#10c	If you are validating a prediction model, describe how the predictions were calculated.	7
Statistical analysis methods	#10d	Specify all measures used to assess model performance and, if relevant, to compare multiple models.	9
Statistical analysis methods	#10e	If you are validating a prediction model, describe any model updating (e.g., recalibration) arising from the validation, if done	n/a
Risk groups	#11	Provide details on how risk groups were created, if done.	n/a
Development vs. validation	#12	For validation, identify any differences from the development data in setting, eligibility criteria, outcome, and predictors.	5
Results			
Participants	#13a	Describe the flow of participants through the study, including the number of participants with and without the outcome and, if applicable, a summary of the follow-up time. A diagram may be helpful.	10
Participants	#13b	Describe the characteristics of the participants (basic demographics, clinical features, available predictors), including the number of participants with missing data for predictors and outcome.	10
Participants	#13c	For validation, show a comparison with the development data of the distribution of important variables (demographics, predictors and outcome).	10
Model development	#14a	If developing a model, specify the number of participants and outcome events in each analysis.	n/a
Model development	#14b	If developing a model, report the unadjusted association, if calculated between each candidate predictor and outcome.	n/a
Model specification	#15a	If developing a model, present the full prediction model to allow predictions for individuals (i.e., all regression coefficients, and model intercept or baseline survival at a given time point).	n/a
Model specification	#15b	If developing a prediction model, explain how to the use it.	n/a

For peer review only

Model performance	#16	Report performance measures (with CIs) for the prediction model.	11 and 12
Model-updating	#17	If validating a model, report the results from any model updating, if done (i.e., model specification, model performance).	n/a, updating not performed
Discussion			
Limitations	#18	Discuss any limitations of the study (such as nonrepresentative sample, few events per predictor, missing data).	13
Interpretation	#19a	For validation, discuss the results with reference to performance in the development data, and any other validation data	12
Interpretation	#19b	Give an overall interpretation of the results, considering objectives, limitations, results from similar studies, and other relevant evidence.	13
Implications	#20	Discuss the potential clinical use of the model and implications for future research	13 and 14
Other information			
Supplementary information	#21	Provide information about the availability of supplementary resources, such as study protocol, Web calculator, and data sets.	14 and 15
Funding	#22	Give the source of funding and the role of the funders for the present study.	15

None The TRIPOD checklist is distributed under the terms of the Creative Commons Attribution License CC-BY. This checklist can be completed online using https://www.goodreports.org/, a tool made by the EQUATOR Network in collaboration with Penelope.ai

For peer review only

BMJ Open

Pregnancy of unknown location: external validation of the hCG based M6NP and M4 prediction models in an emergency gynaecology unit.

Journal:	*BMJ Open*
Manuscript ID	bmjopen-2021-058454.R1
Article Type:	Original research
Date Submitted by the Author:	05-Apr-2022
Complete List of Authors:	Fistouris, Johan; University of Gothenburg Institute of Clinical Sciences, Obstetrics and Gynaecology<br>Bergh, Christina; University of Gothenburg Institute of Clinical Sciences, Obstetrics and Gynaecology<br>Strandell, Annika; University of Gothenburg Institute of Clinical Sciences
**Primary Subject Heading**:	Obstetrics and gynaecology
Secondary Subject Heading:	Obstetrics and gynaecology
Keywords:	ULTRASONOGRAPHY, GYNAECOLOGY, Ultrasound < RADIOLOGY & IMAGING

SCHOLARONE™
Manuscripts

I, the Submitting Author has the right to grant and does grant on behalf of all authors of the Work (as defined in the below author licence), an exclusive licence and/or a non-exclusive licence for contributions from authors who are: i) UK Crown employees; ii) where BMJ has agreed a CC-BY licence shall apply, and/or iii) in accordance with the terms applicable for US Federal Government officers or employees acting as part of their official duties; on a worldwide, perpetual, irrevocable, royalty-free basis to BMJ Publishing Group Ltd ("BMJ") its licensees and where the relevant Journal is co-owned by BMJ to the co-owners of the Journal, to publish the Work in this journal and any other BMJ products and to exploit all rights, as set out in our licence.

The Submitting Author accepts and understands that any supply made under these terms is made by BMJ to the Submitting Author unless you are acting as an employee on behalf of your employer or a postgraduate student of an affiliated institution which is paying any applicable article publishing charge ("APC") for Open Access articles. Where the Submitting Author wishes to make the Work available on an Open Access basis (and intends to pay the relevant APC), the terms of reuse of such Open Access shall be governed by a Creative Commons licence – details of these licences and which Creative Commons licence will apply to this Work are set out in our licence referred to above.

Other than as permitted in any relevant BMJ Author's Self Archiving Policies, I confirm this Work has not been accepted for publication elsewhere, is not being considered for publication elsewhere and does not duplicate material already published. I confirm all authors consent to publication of this Work and authorise the granting of this licence.

For peer review only

Title: Pregnancy of unknown location: external validation of the hCG based M4 and M6NP prediction models in an emergency gynaecology unit.

Author names and affiliations: Johan Fistouris[a], Christina Bergh[a] and Annika Strandell[a]

[a]University of Gothenburg Institute of Clinical Sciences, Department of Obstetrics and Gynaecology, Sahlgrenska University Hospital, Gothenburg, Sweden

Correspondence to: Dr Johan Fistouris, Department of Gynaecology and Reproductive Medicine, Sahlgrenska University Hospital, SE-413 45 Gothenburg, Sweden. (E-mail: johan.fistouris@vgregion.se). Telephone: 0046-31-707552678

Keywords: Chorionic Gonadotropin, Ectopic pregnancy, Pregnancy of Unknown Location, Statistical Models

Word count: 3901

For peer review only

ABSTRACT

Objective: To investigate if M6NP predicting ectopic pregnancy (EP) among women with pregnancy of unknown location (PUL) is valid in an emergency gynaecology setting and comparing it with its predecessor M4.

Design: Retrospective cohort study.

Setting: University hospital

Participants: Women with PUL.

Methods: All consecutive women with a PUL during a study period of 3 years were reviewed for inclusion. Risk prediction of an EP was based on two serum human chorionic (hCG) levels taken at least 24 hours and no longer than 72 hours apart.

Main Outcome Measures: The area under the ROC curve (AUC) and calibration assessed the predictive performance. The proportion of ectopic and non-ectopic pregnancies classified as high risk assessed the model's sensitivity and false positive rate (FPR). The proportion of non-ectopic pregnancies among women classified as low risk was the models negative predictive value (NPV). The clinical utility of a model was evaluated with decision curve analysis.

Results: 1061 women were included in the study, of which 238 (22%) had a final diagnosis of EP. The AUC for EP was 0.85 for M6NP and 0.81 for M4. M6NP made accurate risk predictions of EP up to predictions of 20% but thereafter risk were underestimated. M4 was poorly calibrated up to risk predictions of 40%. With a 5% threshold for high risk classification the sensitivity for EP was 95% for M6NP, the FPR 50% and NPV 97%. M6NP had

For peer review only

higher sensitivity and NPV than M4 but also a higher FPR. M6NP had utility at all thresholds as opposed to M4 that had no utility at thresholds ≤5%.

Conclusions: M6NP had better predictive performance than M4 and is valid in women with PUL attending an emergency gynaecology unit. Our results can encourage implementation of M6NP in related yet untested clinical settings to effectively support clinical decision making.

Strengths and limitations of this study

- This is the first study to test the transportability (generalisability) of the M6NP prediction model to an emergency gynaecology setting, originally developed for early pregnancy assessment units in the UK.
- The rate of ectopic pregnancy was twice as high in comparison to the original M6NP cohort and we also included inpatients contributing to a different case mix.
- A limitation was the complete case analysis; however a sensitivity analysis showed consistent performance for M6NP.
- Another limitation was the retrospective study design, although common for validation studies.

For peer review only

INTRODUCTION

For peer review only

To reduce maternal complications from ectopic pregnancy (EP), an early diagnosis using transvaginal ultrasound (TVUS) is essential [1]. Between 30 and 40 % of EP remain undiagnosed after the first examination of women presenting with pain or bleeding in early pregnancy and will be managed as a pregnancy of unknown location (PUL) until a final diagnosis is established [2]. The reported rupture rate of an EP among women with a PUL range from 0.03 to 2.4% and sometimes necessitates blood transfusion [3-6]. A key concept during follow up is timely diagnosis and treatment of an EP while avoiding unnecessary visits for the remaining majority of women that eventually will be diagnosed with a intrauterin pregnancy (IUP) or a spontaneously resolving PUL (failed PUL) [7]. The change in serum human chorionic gonadotropin (hCG) levels between two samples measured 48 hours apart indicates if a woman is at high or low risk of having an EP and decisive of planning follow- up visits [8]. In a recent meta-analysis it was concluded that the widely reported logistic regression model “M4” that is based on two hCG, was the best available method to predict the presence of an EP [9-12].

M4 was developed in an dedicated early pregnancy assessment unit (EPAU) in London and has mostly been evaluated in similar clinical settings within that geographical region [10]. The M4 model was updated in a larger cohort of PUL in 2016 with an option to include progesterone as a predictor (M6P) or not (M6NP) [13]. According to recent research M6 to date has the best predictive performance when applied on women in EPAU [14]. The performance of a prediction model is influenced by the characteristics of the underlying population and may not be generalizable to a different clinical context in another country with the risk of being harmful if implemented [15]. For instance when M4 was tested on patients in a US emergency department its ability to distinguish between EP and non- measured with the area under the receiver operating characteristic (ROC) curve was 0.83 but

For peer review only

the sensitivity for identifying an EP was only 55% [16]. Neither of M6NP or M6P has been validated in an emergency gynaecology.

The primary aim of the present study was to externally validate M6NP in an emergency gynaecology unit and compare the performance to its predecessor M4.

MATERIALS AND METHODS

Design and Setting

External validation was performed on women with PUL attending the emergency gynaecology unit at the Sahlgrenska University Hospital from January 1st, 2011 to December 31st, 2013.

The emergency gynaecology unit is open 24 hours a day, 7 days a week and serves patients with various gynaecological problems either self-referred or referred from other clinics in the greater Gothenburg area. The study hospital was the only referral site for the treatment of EP in the area as opposed to other early pregnancy complications. Initial examination of women with early pregnancy complaints was performed by a physician in early stage of specialist training and a consultant gynaecologist assisted upon request. Serial hCG measurments was applied whenever the physician was uncertain about the location of the pregnancy based on TVUS and clinical findings.

The second blood sample for hCG measurement were routinely scheduled between two and three days after the first hCG. Since some women make unplanned visits during follow-up, the interval can be shorter than two days. Interpretation of hCG development was not based on any specific percentage change but approximations of the doubling time in hCG for a viable IUP and the expected hCG decline in miscarriage/failed PUL [17]. Our routine for

For peer review only

outpatients' surveillance has been described in a prior study from our emergency gynaecology unit [6]. Outpatients with deteriorating symptoms were instructed to re- visit the unit. Depending on the severity of symptoms and clinical findings, inpatient evaluation and further investigation by a senior physician was considered.

Laparoscopic intervention was the first line of treatment for EP during the study period. A diagnostic laparoscopy was also performed in patients with a prolonged period of follow-up where hCG levels did not decline and there was no evidence of an IUP. In cases where laparoscopy failed to identify an EP uterine evacuation followed to establish a final diagnosis. Methotrexate was used if there was persistent trophoblast after initial surgical treatment or as treatment of interstitial, cervical or caesarean scar pregnancies.

Participants

Women presenting to our emergency gynaecology unit with complaints in early pregnancy, deemed to have a PUL were screened for inclusion. All women with at least two hCG levels were eligible for inclusion. Also, women that were initially managed as outpatients but the second hCG was taken during or after a hospital stay were included unlike prior studies.

In the original M6NP study the second hCG level was imputed if it was taken more than 3 days after the first hCG [13]. However in clinical practice the second hCG has only been used for prediction if taken 48±8 hours after the first hCG [3]. We decided to include women with a sampling interval between 24 and 72 hours so that the validation cohort represented the actual PUL management in our clinical setting. We excluded women where only one hCG was taken or the final outcome of PUL was unknown (lost to follow-up).

Data Collection

For peer review only

The following variables were obtained from the patients electronical medical record: Age at the first visit; symptoms related to the first visit (i.e. pain or bleeding); prior EP or use of a contraceptive intrauterine device; level of the first and second serum hCG (IU/L); date and time of hCG measurments; the type of PUL and the final outcome of the pregnancy. All serum hCG levels were analysed in the same laboratory.

We defined three types of a PUL based on TVUS at the first visit or during follow-up [18]: no signs of a pregnancy neither inside nor outside of the uterus defined a true PUL; when an intrauterine sac-like structure or suspicion of retained products of conception was seen it was defined as a probable IUP; probable EP was used when an inhomogeneous adnexal mass or ectopic sac-like structure was seen (classification of different ectopic sites was according to an ESHRE recommendation) [19]. If a gestational sac containing a yolk sac with or without a foetus was seen with TVUS it was defined as a definite IUP or EP and therefore not a PUL [18].

Prediction models

M4 and M6NP use polynomial logistic regression to compute probabilities for each of the three possible outcomes of a PUL: failed PUL, IUP and EP. The covariates of both models are based on two hCG levels (IU/L) analysed in serum approximately two days apart (Supplemental Table 1).

We used the original equations (Supplemental Material) of M6NP and M4 to compute probabilities as recommended instead of refitting the model [15, 20]. M6P was not validated since progesterone was not used in our unit when managing PUL.

Reference Standard

For peer review only

For peer review only - http://bmjopen.bmj.com/site/about/guidelines.xhtml

The reference standard was based on the combination of hCG development, TVUS findings, and surgical procedures such as laparoscopy and vacuum aspiration. There are four categories of PUL outcome and these were set as reference standard: (1) A failed PUL was defined by spontaneously declining hCG levels below 0.6 IU/L or a negative pregnancy test during follow-up where nor an IUP or EP could be confirmed. (2) An IUP regardless of viability was diagnosed by either TVUS; histological findings of chorionic villi after vacuum aspiration; completion of a suspected miscarriage after medical or surgical treatment verified by TVUS or a decline of hCG to <0.6 IU/L. (3) An EP was diagnosed with TVUS, at laparoscopy or hysteroscopy with or without confirmed histology. (4) A persisting PUL (PPUL) was defined when no pregnancy was visible on TVUS with plateauing hCG levels (at least three consecutive hCG with a change <15% between measurments during a one week follow-up). If no chorionic villi were present in uterine aspiration specimen or if none was analysed and hCG levels remained elevated also defined a persisting PUL regardless of prior hCG development as were non-visualised pregnancies treated with methotrexate

In the analysis PPUL were incorporated among EP as in previous studies [4, 11, 13]. We used the same definitions as in the original M4 and M6NP studies and a consensus statement [10, 13, 18].

The TRIPOD (Transparent Reporting of a multivariable for individual Prognosis or Diagnosis) guidelines were followed conducting this study [21].

Study Size

No sample size calculation was performed. A validation cohort should contain at least 100 events and ideally 200 events to adequately evaluate prediction rules [22]. Our validation cohort contained 238 EP satisfying this requirement.

For peer review only

Patient and public involvement statement

Patients and the public were not involved in the development of the research question.

Statistical Analysis

The predictive performance of a model was evaluated with discrimination and calibration [23]. We focused on the dichotomous outcome of EP and non-EP (IUP and failed PUL) as being the most important to distinguish in clinical practice. Discrimination refers to how well the predicted probabilities of a model differentiates between EP and non-EP and was expressed as the area under the ROC curve (AUC).

Calibration describes the agreement between the predicted risk of an EP and the observed risk (proportion) of EP [23]. We assessed calibration with calibration-in-the large, a calibration plot and the calibration slope. Calibration-in-the-large is the average predicted risk of EP in the entire cohort compared with actual rate of EP. The calibration plot shows prediction accuracy for each patient individually with a computed lowess smoothing curve [23, 24]. If the intercept (which is an assessment of calibration in the large) of the curve is >0 the average predicted risk of EP is lower than the actual proportion of EP in the cohort and if the intercept is <0 the average predicted risk is higher. A slope < 1 indicates that (on average) high risk predictions are overestimated and low risk predictions are underestimated. A slope > 1 indicates that (on average) high risk predictions are underestimated and low risk predictions are overestimated [25].

Classification accuracy was measured with sensitivity (proportion of EP classified as high risk), false positive rate (FPR), (proportion of non-EP classified as high risk) and the negative predictive value (proportion of non-EP among women classified as low risk) as it was

For peer review only

presented in the original M6NP study [13]. In implementation studies from EPAU in the UK a PUL has been classified as high risk of EP if the predicted probability of EP reached a threshold of 5% [14, 26]. An optimal threshold can vary between clinical settings; we therefore explored other plausible thresholds (2.5, 5, and 10%) for classification as recommended when validating prediction models [27].

To determine which model that would lead to the best clinical outcomes (clinical utility) if used for clinical decision making the Net Benefit (NB) was measured at the 5% threshold and over a range of thresholds in a decision curve [28]. The NB is calculated as the difference between the number of true positives (correctly identified EP) and false positives (misclassified non-EP) where the latter is weighed by the probability threshold (Supplemental). The choice of a certain threshold for classification reflects how the clinician values a true positive in relation to a false positive. A lower threshold implies that a true positive is valued higher than if a higher threshold is used. For instance, the 5% (odds 1:19) threshold translates to an acceptance of 19 false positives per correctly identified EP. The models´ NB was compared with default strategies of assuming all as positive (high risk of EP) and none as positive in a decision curve. If a model has a NB below a default strategy at a specific threshold it has no clinical utility at that threshold.

Since we used a complete case analysis for the validation cohort we performed a sensitivity analysis to test if the exclusion of women with a sampling interval between two hCG that were shorter than 24 hours or longer than 72 hours presented a risk for introducing bias [29]. The sensitivity analysis thus included all women regardless of hCG sampling interval.

For peer review only

Performance measures are presented with 95% confidence intervals (CI). McNemar´s test was used for testing differences of paired proportions. DeLong's test was used for testing the difference in AUC. A p-value of < 0.05 was considered statistically significant.

Statistical analyses were performed using SPSS 21.0 (SPSS, Chicago, IL, USA) and SAS version 9.4 (Cary, NC, USA).

RESULTS

A total of 1447 consecutive women with PUL were reviewed for inclusion into the validation cohort as shown in the flowchart (Fig. 1). There were 239 women that only had one hCG taken and six women with a final outcome of a gestational trophoblastic disease and were therefore not included in the study. Another 125 women were excluded due to having an hCG sampling interval outside 24 to 72 hours. Among all 1447 women, 37 were lost to follow-up.

Descriptive statistics including hCG data for 1061 women in the validation cohort and those that were eligible but lost to follow-up (n=15) are presented in Table 1. In the validation cohort 71% of women (751/1061) had an hCG sampling interval of 48±8 hours and 8% (89/1061) were managed as inpatients before the second hCG was taken. Final outcomes of PUL were 461 failed PUL, 362 IUP of which 98 were non-viable and 238 EP (22%) (including 46 PPUL) (Supplemental Table 2).

Supplemental Table 2 includes data from the original M4 and the M6NP cohorts where the rate of EP was 6% and 11% respectively. The median of the first hCG level was numerically higher and the median hCG ratio was lower for EP in our validation cohort as compared with EP in the original M6NP cohort (Supplemental Table 2). Also for IUP the median of the first

For peer review only

hCG level was numerically higher but the median hCG ratio was lower in our cohort. Differences in hCG characteristics appears to be lower for failed PUL when comparing our cohort with the original M6NP cohort.

Discrimination and Calibration

The AUC for EP was higher for M6NP, 0.85 (95% CI 0.82–0.88) than for M4, 0.81 (95% CI 0.78–0.84) ($p<0.01$). As seen in the calibration plot M6NP was well calibrated up to predictions of 0.20 with the curve close to the diagonal line, but underestimated risk thereafter (Fig. 2A). M4 made poorly calibrated risk predictions of EP up to 0.40 (Fig.2B). Calibration in the large showed that the average predicted risk of EP by both models was underestimated, especially M4 as the intercept was further from 0 as compared with M6NP (Fig. 2). The average predicted risk by M4 among higher risk groups was overestimated and underestimated among lower risk groups as indicated by the slope being < 1, the slope was below but closer to 1 for M6NP (Fig. 2).

Classification and Clinical Utility

At the 5% risk threshold M6NP had higher sensitivity for identifying an EP, 95% (95% CI 92–98) than M4, 85% (95% CI 81–90) as presented in Table 2. The FPR was 37% for M4 and 50% for M6NP ($p<0.01$). The negative predictive value (NPV) for M4 was 94% and 97% for M6NP ($p<0.01$). M4 and M6NP classified 52% (95% CI: 50–55) and 40% (95% CI: 37–42) of PUL as low risk respectively ($p<0.01$). Less than 50% of failed PUL rendered 5% risk predictions of an EP by M6NP and 75% of IUP (Supplemental Figure 1). This equals the proportion of failed PUL and IUP being misclassified as EP by M6NP. M6NP misclassified twelve EP as low risk of which seven were predicted to have a failed PUL. Among these EP, one had emergency

For peer review only

surgery due to rupture during follow-up, but no blood transfusion was needed.

Supplemental Table 3 presents details for all EP misclassified by M6NP.

At the 10% threshold M6NP had a sensitivity of 91%, FPR of 39% and NPV of 96% (Table 2). The sensitivity was lower at 80% for M4 and the FPR was improved but not as marked as for M6NP. At the 2.5% threshold as compared with the 5% threshold the sensitivity and NPV were only marginally higher for M6NP while the FPR increased from 50 to 61% and the proportion of PUL classified as low risk decreased from 40 to 31% (Table 2).

Decision curve analysis showed that the curve for M6NP was above that of M4 and the default strategy of assuming all as positive over the entire range of thresholds (Fig. 3). This means that M6NP had higher NB than M4 and utility at any threshold. M4 had no utility at risk thresholds ≤5% as the curve was under the reference curve of assuming all women as positive.

Sensitivity Analysis

In the sensitivity analysis all women with at least two hCG and any sampling interval were included. Descriptive statistics for all 1186 women were similar to those in the validation cohort except the hCG sampling interval (Supplemental Table 4). Both models had marginally lower AUC (Supplemental Table 3). The calibration for both models was similar (Supplemental Fig. 2). The sensitivity and the FPR at the 5% risk threshold were almost the same as in the validation cohort for both models, although slightly lower (Supplemental Table 5).

DISCUSSION

For peer review only

This external validation study found that M6NP perform similar in a Swedish emergency gynaecology unit as in EPAU in the UK where it was developed. M6NP made more accurate predictions of an EP and could better differentiate an EP from a non-EP than M4. The main difference between the models in a clinical perspective was that M6NP classified fewer PUL as low risk than M4 leading to its higher sensitivity and false positive rate at the 5% threshold. This could result in more women being part of some sort of close monitoring if clinical decision making is based on M6NP as compared with M4, but importantly an opportunity for early diagnosis of more EP starting as PUL. At the 10% threshold M6NP maintained a high sensitivity while the FPR was significantly lower. M6NP had clinical utility at any threshold unlike M4 that had no clinical utility at thresholds ≤5%.

For peer review only

This is the first study to test the generalisability of the M6NP prediction model in an emergency gynaecology unit which is an important step before a prediction model can be recommended for, or discouraged from wider implementation [30]. We present real world data on how M6NP would perform in a setting that in several aspects are different from the original cohort with encouraging results.

One limitation of the study complete case analysis approach. However a sensitivity analysis that included all women regardless of sampling interval between two hCG revealed that predictive performance and classification accuracy were sparsely affected. We have no reason to believe that validation cohort is not representative of the target population and the sample size after exclusion was still large enough for inference about the models' predictive performance [21]. A second limitation was the retrospective design of the study although common when validating or developing prediction models as in the original M6NP study [15]

322 The average predicted probability of EP in the entire cohort (calibration in the large) was underestimated, especially for M4, which was an effect of the lower rate of EP in the original cohorts [10, 13]. M6NP was well calibrated up to predictions of 0.20 contrary to M4. Instead predictions by M4 were clearly too low up to predictions of 0.40, also reported in a recent validation study from EPAU in the UK [14]. This implies that predictions made by M6NP unlike M4 were accurate at thresholds relevant for clinical decision making, and underestimation at higher thresholds may thus be irrelevant in this respect [23].

329 M6NP mainly misclassified IUP and to a lesser extent failed PUL in our cohort as reported for M6P [3]. However, failed PUL in our cohort generated higher risk predictions of an EP, compared to failed PUL in in the validation study from the UK and likely in the original cohort. A greater number of failed PUL was therefore misclassified as high risk at the 5% threshold leading to a higher FPR in our cohort [13, 14]. The reasons for discrepancies between risk predictions could be because of differences in sampling interval between two hCG, inclusion of inpatients and that the median hCG levels in our study were generally higher compared to prior studies. Also, the use of imputation in previous studies when the second hCG was missing completely or when the second hCG was taken more than 3 days after the first could have contributed.

339 We did not assess M6P (adding progesterone as a predictor) since progesterone is not used in our clinic. As reported in recent studies from EPAU in both the UK and Australia it is likely that M6P would perform better as compared with M6NP in our unit [14, 31].

342 In clinical practice women with a predicted failed PUL after just two hCG measurments are followed up with a home urine pregnancy test after two weeks and if an IUP is predicted examination is repeated after one week. Women with a PUL classified as high risk of EP are

re-examined within 24 hours [3, 32]. In such clinical situation high classification accuracy is crucial for a prediction model to be useful. In our study M6NP only misclassified 5% (n=12) of EP as low risk. On the other hand, 50% of non-EP were misclassified as high risk which could result in high numbers of unnecessary visits, admissions to the ward and potentially interventions if implemented. There is insufficient data to rely on when it comes to costs and benefits of misclassifying women with a PUL which makes it difficult to define an optimal threshold for high risk classification, a general challenge when implementing a prediction model in clinical practice [27]. When M4 and M6NP have been used in clinical practice it has been considered reasonable to allow 19 misclassified non-EP for every correctly classified EP which equals the 5% risk threshold [3, 12]. Our population had a high rate of EP and the 5% threshold therefore seems justifiable to optimize sensitivity. The sensitivity for EP remained high for M6NP at the 10% threshold while the false positive rate decreased from 50 to 39%. The trade-off between sensitivity and false positive rate at the 2.5% threshold was less favourable since the false positive rate increased significantly and the sensitivity was only marginally higher as compared with the 5% threshold. M6NP had clinical utility at the 5 and 10% thresholds we therefore suggest that any of these are appropriate to use in our clinical setting.

Regardless which threshold that is finally implemented one must remember that M6NP should always be used as a compliment to clinical judgement and never as a sole decision instrument. M6NP and M6P are available at www.earlypregnancycare.com/m6 where the 5% threshold is used by default.

CONCLUSION

For peer review only

The performance of M6NP as opposed to M4 was encouraging when validated on women with PUL in an emergency gynaecology unit. M6NP can be introduced in related yet untested clinical settings outside the UK and EPAU to effectively support clinical decision making. Updating of M6NP in a local context to account for geographical differences could be an option to improve calibration.

Contributors J.F. and A.S. contributed substantially to conception and design of the study. J.F collected the data. J.F performed some of the statistical analysis and J.F and A.S interpreted the data. J.F. and A.S. wrote the article. J.F drafted the article. C.B. commented on the drafts. All authors made revisions to the article and approved the final version.

Funding This work was supported by grants from the Swedish state under the agreement between the Swedish government and the county councils, the ALF-agreement [ALFGBG-720291, ALFGBG-72150] and The Healthcare Board, Region Västra Götaland.

Competing interests None declared.

Ethics approval statement approval for this study was obtained from the Regional Ethical Review Board, Gothenburg, Sweden (approval # 50115).

Data availability statement Data are available on reasonable request. Requests should be made to the lead author johan.fistouris@vgregion.se.

REFERENCES

For peer review only

1. Elson J, Salim R, Potdar N, Chetty M, Ross JA ,Kirk E. on behalf of the Royal College of Obstetricians and Gynaecologists. Diagnosis and management of ectopic pregnancy. BJOG 2016;.123:e15–e55. BJOG 2016; 123: e15-e55.
2. Crochet J BL. Does ThisWoman Have an Ectopic Pregnancy? The Rational Clinical Examination Systematic Review. JAMA 2013; Vol 309: 1722-29.
3. Bobdiwala S, Christodoulou E, Farren J, Mitchell-Jones N, Kyriacou C, Al-Memar M, et al. Triaging women with pregnancy of unknown location using two-step protocol including M6 model: clinical implementation study. Ultrasound Obstet Gynecol 2020; 55: 105-14.
4. Nadim B, Leonardi M, Infante F, Lattouf I, Reid S ,Condous G. Rationalizing the management of pregnancies of unknown location: Diagnostic accuracy of human chorionic gonadotropin ratio-based decision tree compared with the risk prediction model M4. Acta Obstet Gynecol Scand 2020; 99: 381-90.
5. Morse CB, Sammel MD, Shaunik A, Allen-Taylor L, Oberfoell NL, Takacs P, et al. Performance of human chorionic gonadotropin curves in women at risk for ectopic pregnancy: exceptions to the rules. Fertil Steril 2012; 97: 101-6 e2.
6. Fistouris J, Bergh C ,Strandell A. Classification of pregnancies of unknown location according to four different hCG-based protocols. Hum Reprod 2016; 31: 2203-11.
7. Kirk E, Bottomley C ,Bourne T. Diagnosing ectopic pregnancy and current concepts in the management of pregnancy of unknown location. Hum Reprod Update 2014; 20: 250-61.
8. van Mello NM, Mol F, Opmeer BC, Ankum WM, Barnhart K, Coomarasamy A, et al. Diagnostic value of serum hCG on the outcome of pregnancy of unknown location: a systematic review and meta-analysis. Hum Reprod Update 2012; 18: 603-17.
9. Bobdiwala S, Saso S, Verbakel JY, Al-Memar M, Van Calster B, Timmerman D, et al. Diagnostic protocols for the management of pregnancy of unknown location: a systematic review and meta-analysis. BJOG 2019; 126: 190-98.
10. Condous G, Van Calster B, Kirk E, Haider Z, Timmerman D, Van Huffel S, et al. Prediction of ectopic pregnancy in women with a pregnancy of unknown location. Ultrasound Obstet Gynecol 2007; 29: 680-7.
11. Guha S, Ayim F, Ludlow J, Sayasneh A, Condous G, Kirk E, et al. Triaging pregnancies of unknown location: the performance of protocols based on single serum progesterone or repeated serum hCG levels. Hum Reprod 2014; 29: 938-45.
12. Bobdiwala S, Guha S, Van Calster B, Ayim F, Mitchell-Jones N, Al-Memar M, et al. The clinical performance of the M4 decision support model to triage women with a pregnancy of unknown location as at low or high risk of complications. Hum Reprod 2016; 31: 1425-35.
13. Van Calster B, Bobdiwala S, Guha S, Van Hoorde K, Al-Memar M, Harvey R, et al. Managing pregnancy of unknown location based on initial serum progesterone and serial serum hCG levels: development and validation of a two-step triage protocol. Ultrasound Obstet Gynecol 2016; 48: 642-49.
14. Christodoulou E, Bobdiwala S, Kyriacou C, Farren J, Mitchell-Jones N, Ayim F, et al. External validation of models to predict the outcome of pregnancies of unknown location: a multicentre cohort study. BJOG 2021; 128: 552-62.
15. Moons KG, Kengne AP, Grobbee DE, Royston P, Vergouwe Y, Altman DG, et al. Risk prediction models: II. External validation, model updating, and impact assessment. Heart 2012; 98: 691-8.
16. Barnhart KT, Sammel MD, Appleby D, Rausch M, Molinaro T, Van Calster B, et al. Does a prediction model for pregnancy of unknown location developed in the UK validate on a US population? Hum Reprod 2010; 25: 2434-40.
17. Butts SF, Guo W, Cary MS, Chung K, Takacs P, Sammel MD, et al. Predicting the decline in human chorionic gonadotropin in a resolving pregnancy of unknown location. Obstet Gynecol 2013; 122: 337-43.

For peer review only

18. Barnhart K, van Mello NM, Bourne T, Kirk E, Van Calster B, Bottomley C, et al. Pregnancy of unknown location: a consensus statement of nomenclature, definitions, and outcome. Fertil Steril 2011; 95: 857-66.
19. Pregnancy EwgoE, Kirk E, Ankum P, Jakab A, Le Clef N, Ludwin A, et al. Terminology for describing normally sited and ectopic pregnancies on ultrasound: ESHRE recommendations for good practice. Hum Reprod Open 2020; 1-21.
20. Altman DG, Vergouwe Y, Royston P ,Moons KG. Prognosis and prognostic research: validating a prognostic model. . BMJ 2009; 338: b605.
21. Collins GS, Reitsma JB, Altman DG ,Moons KG. Transparent reporting of a multivariable prediction model for individual prognosis or diagnosis (TRIPOD): the TRIPOD statement. BMJ 2015; 350: g7594.
22. Collins GS, Ogundimu EO ,Altman DG. Sample size considerations for the external validation of a multivariable prognostic model: a resampling study. Stat Med 2016; 35: 214-26.
23. Alba AC, Agoritsas T, Walsh M, Hanna S, Iorio A, Devereaux PJ, et al. Discrimination and Calibration of Clinical Prediction Models: Users' Guides to the Medical Literature. JAMA 2017; 318: 1377-84.
24. Austin PC ,Steyerberg EW. Graphical assessment of internal and external calibration of logistic regression models by using loess smoothers. Stat Med 2014; 33: 517-35.
25. Van Calster B, Nieboer D, Vergouwe Y, De Cock B, Pencina MJ ,Steyerberg EW. A calibration hierarchy for risk models was defined: from utopia to empirical data. J Clin Epidemiol 2016; 74: 167-76.
26. Van Calster B, Abdallah Y, Guha S, Kirk E, Van Hoorde K, Condous G, et al. Rationalizing the management of pregnancies of unknown location: temporal and external validation of a risk prediction model on 1962 pregnancies. Hum Reprod 2013; 28: 609-16.
27. Wynants L, van Smeden M, McLernon DJ, Timmerman D, Steyerberg EW, Van Calster B, et al. Three myths about risk thresholds for prediction models. BMC Med 2019; 17: 192.
28. Vickers AJ ,Elkin EB. Decision curve analysis: a novel method for evaluating prediction models. Med Decis Making 2006; 26: 565-74.
29. Joshi M, Royuela A ,Zamora J. Proper analysis in clinical trials: how to report and adjust for missing outcome data. BJOG 2013; 120: 915-19.
30. Ramspek CL, Jager KJ, Dekker FW, Zoccali C ,van Diepen M. External validation of prognostic models: what, why, how, when and where? Clin Kidney J 2021; 14: 49-58.
31. Ooi S, De Vries B ,Ludlow J. How do the M4 and M6 models perform in an Australian pregnancy of unknown location population? Aust N Z J Obstet Gynaecol 2021; 61: 100-05.
32. NICE guideline [NG126]. https://www.nice.org.uk/guidance/ng126 (Accessed September 14, 2021) 2019.

For peer review only

Figure Legends

Figure 1. Flow chart showing the inclusion of patients in the validation cohort and the sensitivity analysis. PUL, pregnancy of unknown location; hCG, human chorionic gonadotropin.

Figure 2. (A) The calibration plot shows that the curve for M6NP, with pointwise 95% confidence interval (shaded blue area), are close to the line at 45 degrees up to predicted probabilities of 0.20. This indicates that risk predictions for ectopic pregnancies are accurate in this range. When the predicted risk is 40% however the observed proportion of ectopic pregnancy is ~50% which means that risk of ectopic pregnancy is underestimated. (B) M4 underestimates risk of ectopic pregnancy up to estimates of 0.40 as the curve is clearly above the line at 45 degrees in this range.

Figure 3. Decision curve analysis for M4 (dashed red line) and M6NP (blue line). The high risk threshold is above which a pregnancy of unknown location is classified as high risk (positive) of ectopic pregnancy. M6NP has a higher Net Benefit compared to M4 and default strategies of assuming all (dashed green line) or none (grey line) of PUL as high risk over the entire range of thresholds. M6NP thus has utility at any threshold as compared to M4 that has no utility ≤5%.

For peer review only

Table 1 Descriptive statistics of patients in the validation cohort and patients lost to follow-up.

Characteristics	Validation cohort *n*=1061	Lost to follow-up *n*=15
Age (years)		
Median (Q1;Q3)	31 (26;35)	33 (29;36)
Range	15–49	20–44
First hCG value (IU/L)		
Median (Q1;Q3)	703 (210;2312)	640 (272;6325)
Range	7–180000	19–2800
Second hCG value (IU/L)		
Median (Q1;Q3)	635 (150;2700)	810 (120:8100)
Range	1–180000	21–3500
hCG ratio (second hCG/first hCG)		
Median (Q1;Q3)	0.94 (0.45;1.59)	1.1 (0.79;1.53)
Range	0.06–5.65	0.23–1.79
Hours between two hCG samples, n (%)		
<24	0 (0)	0 (0)
24–39	141 (13)	2 (13)
40–56	751 (71)	7(47)
57–72	169 (16)	3 (20)
>72	0 (0)	0 (0)
Type of PUL, n (%)		
True PUL	793 (75)	11 (73)
Probable IUP	239 (22)	4 (27)
Probable EP	29 (3)	0 (0)
Vaginal bleeding, n (%)	723 (68)	8 (53)
Prior ectopic pregnancy, n (%)	76 (7)	1 (6)
IUD, n (%)	30 (3)	0 (0)

hCG, human chorionic gonadotropin; Q1, first interquartile; Q3, third interquartile; PUL, pregnancy of unknown location; IUP, intrauterine pregnancy, EP, ectopic pregnancy; IUD, intrauterine device. Because of rounding the summarised percentage sometimes are not exactly 100%.

512

513

For peer review only

Table 2. Classification accuracy of the M6NP and M4 at the 2.5, 5 and 10% threshold.

Threshold	Accuracy measure	M6NP	M4	*P*-value
2.5%	Sensitivity	97 (94–99)	91 (88–95)	<0.01
	False positive rate	61 (58–65)	43 (40–46)	<0.01
	Negative predictive value	98 (96–99)	96 (94–97)	<0.01
	Percentage classified as low risk	31 (28–34)	46 (44–50)	<0.01
5% (used in previous studies)	Sensitivity	95 (92–98)	85 (81–90)	<0.01
	False positive rate	50 (47–54)	37 (34–40)	<0.01
	Negative predictive value	97 (96–99)	94 (92–96)	<0.01
	Percentage classified as low risk	40 (37–42)	52 (50–55)	<0.01
10%	Sensitivity	91 (87–94)	80 (75–85)	<0.01
	False positive rate	39 (36–43)	30 (37–34)	<0.01
	Negative predictive value	96 (94–98)	92 (90–94)	<0.01
	Percentage classified as low risk	49 (46–52)	58 (56–61)	<0.01

Reported as percentage (95% confidence interval)

For peer review only

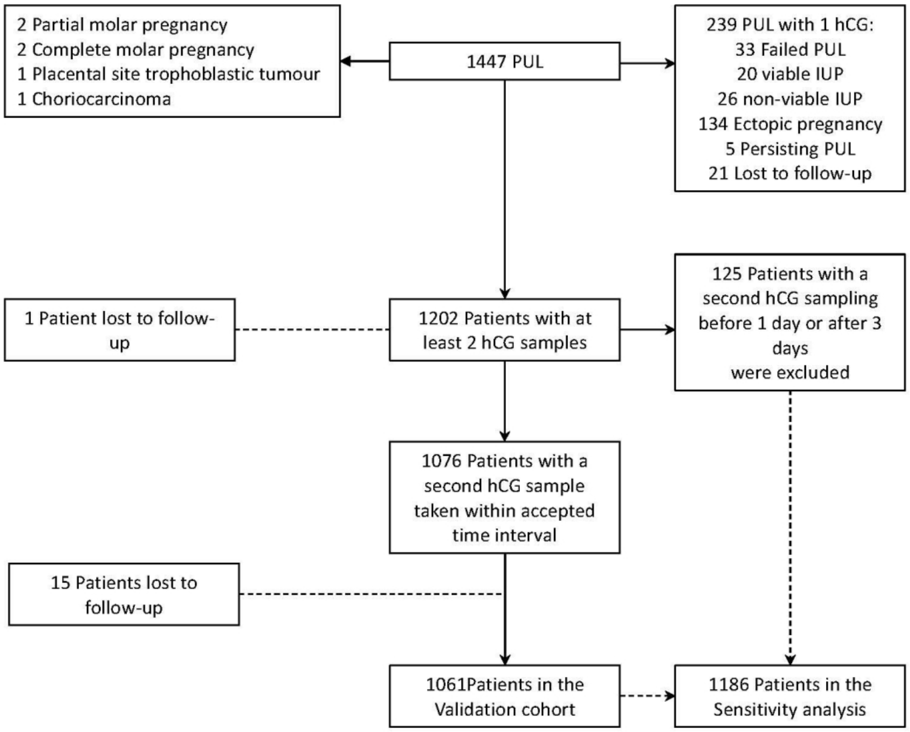

Flow chart showing the inclusion of patients in the validation cohort and the sensitivity analysis. PUL, pregnancy of unknown location; hCG, human chorionic gonadotropin.

208x163mm (200 x 200 DPI)

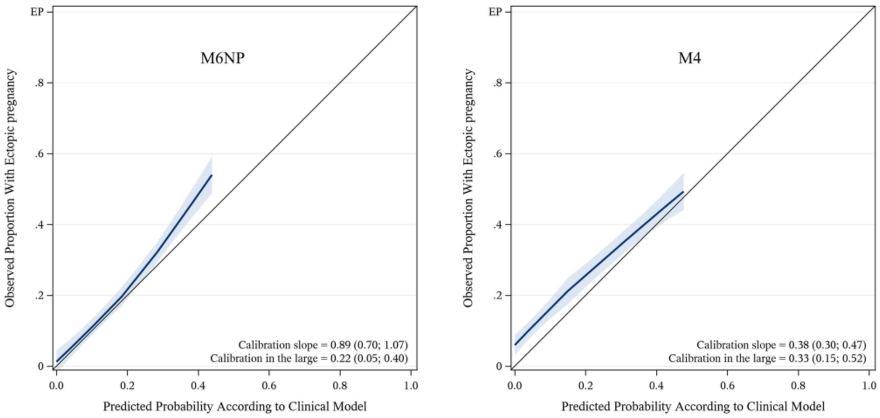

(A) The calibration plot shows that the curve for M6NP, with pointwise 95% confidence interval (shaded blue area), are close to the line at 45 degrees up to predicted probabilities of 0.20. This indicates that risk predictions for ectopic pregnancies are accurate in this range. When the predicted risk is 40% however the observed proportion of ectopic pregnancy is ~50% which means that risk of ectopic pregnancy is underestimated. (B) M4 underestimates risk of ectopic pregnancy up to estimates of 0.40 as the curve is clearly above the line at 45 degrees in this range.

253x190mm (300 x 300 DPI)

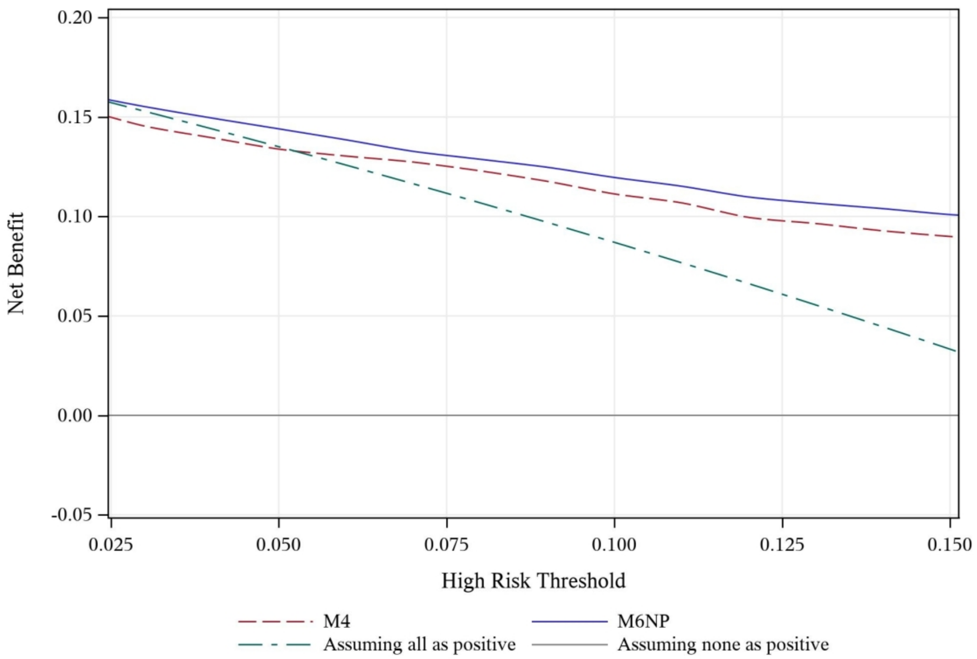

Decision curve analysis for M4 (dashed red line) and M6NP (blue line). The high risk threshold is above which a pregnancy of unknown location is classified as high risk (positive) of ectopic pregnancy. M6NP has a higher Net Benefit compared to M4 and default strategies of assuming all (dashed green line) or none (grey line) of PUL as high risk over the entire range of thresholds. M6NP thus has utility at any threshold as compared to M4 that has no utility ≤5%.

169x127mm (300 x 300 DPI)

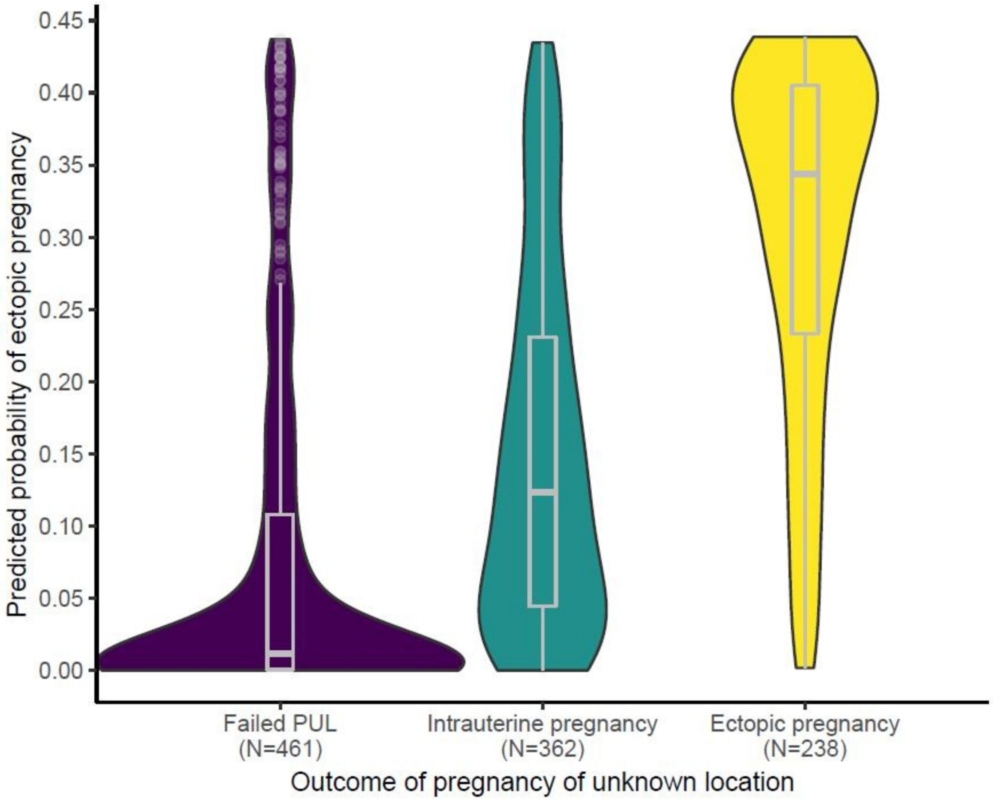

The violin plot shows the full distribution of the predicted probabilities by M6NP of an ectopic pregnancy within each outcome of a pregnancy of unknown location in the validation cohort. The boxes inside the violin plot express the median (Q1; Q3). The plot indicates that less than 50% of failed PUL rendered 5% risk predictions of an ectopic pregnancy and almost 75% of intrauterine pregnancies. This equals the proportion being misclassified as ectopic pregnancies. PUL, pregnancy of unknown location; Q1, first quartile; Q3, third quartile.

230x187mm (96 x 96 DPI)

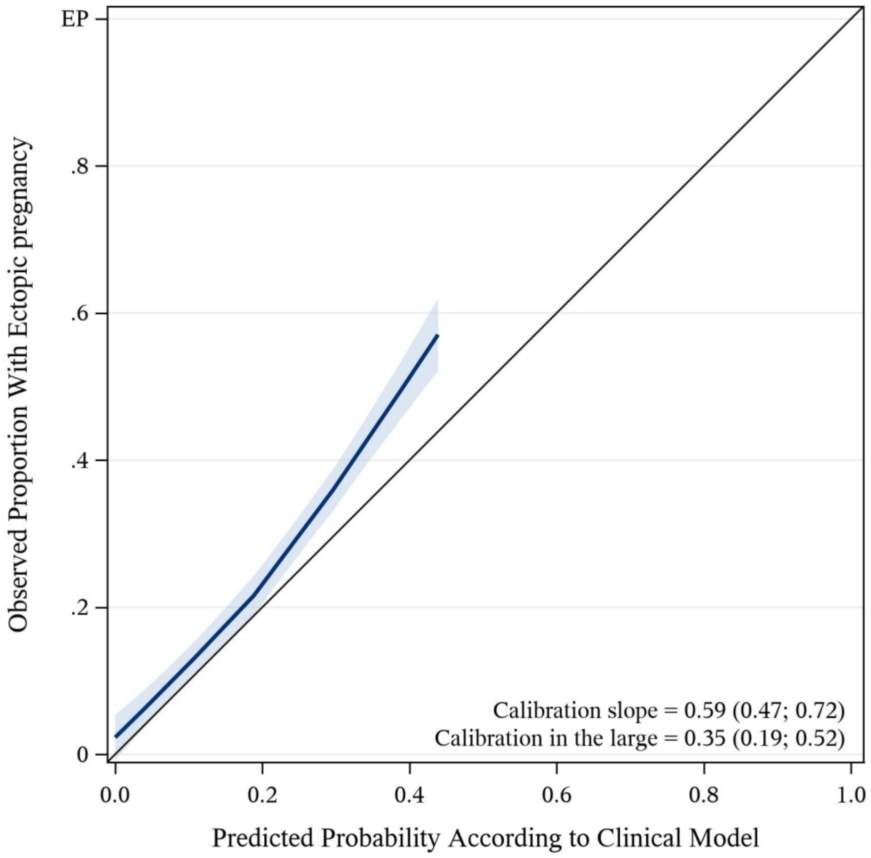

(A) The calibration plot shows the agreement between the predicted and observed probability of ectopic pregnancy by M6NP (blue line).

149x137mm (300 x 300 DPI)

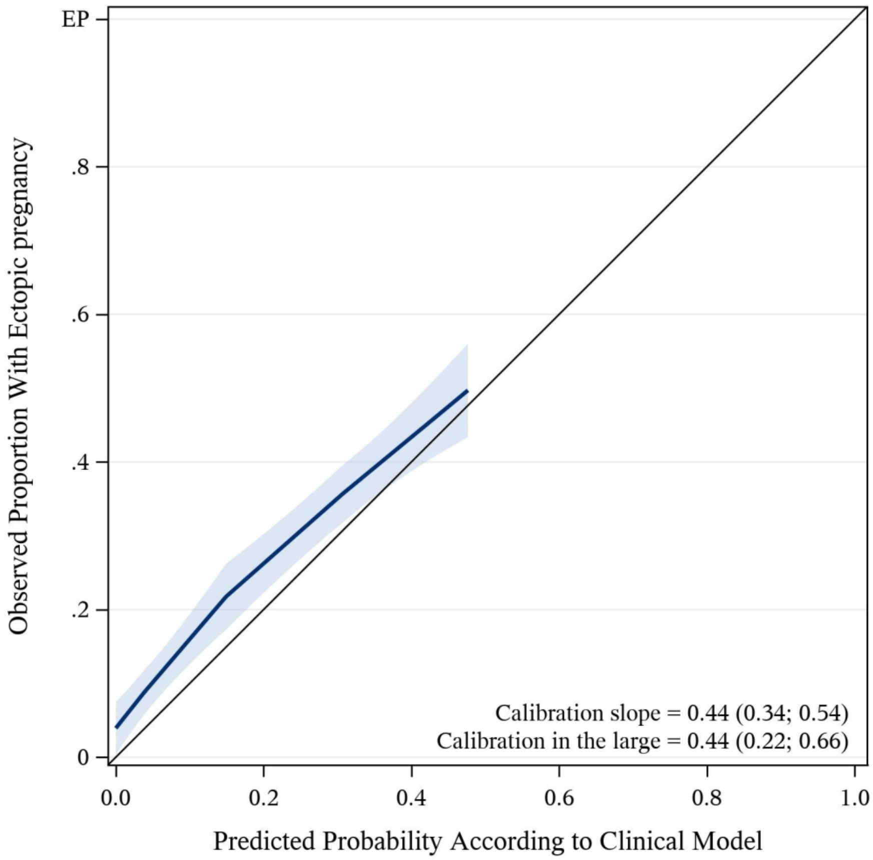

(B) The calibration plot shows the agreement between the predicted and observed probability of ectopic pregnancy by M4 (blue line).

148x137mm (300 x 300 DPI)

Supplemental

Supplemental Table 1. Covariates used in the original M4 [1] and M6NP [2] cohorts.

Model	Covariate	Description
M4	log(average hCG)	Log transformation of (first hCG + second hCG)/2
M4	centered hCG ratio	Centering transformation of (second hCG /first hCG-1.17)
M4	(centered hCG ratio)^2	The quadratic effect was added because the linearity for the logit assumption was violated for hCG ratio.
M6NP	log(initial hCG)	Log transformation of the first hCG
M6NP	log(hCG ratio)	Log transformation of (second hCG/first hCG)
M6NP	[log(hCG ratio)]^2	Square of the log transformed hCG ratio

Log refers to the natural logarithm. hCG = human chorionic gonadotropin.

[a]Average hCG ratio in the original M4 cohort. Centering reduced the correlation between hCG ratio and its square from 0.96 to 0.59.

For peer review only

Supplemental Table 2. Descriptive statistics of failed PUL, intrauterine pregnancies and ectopic pregnancies in the validation cohort and the original M4 and M6NP cohorts.

Outcome of PUL/variable	Validation cohort	Original M4 cohort	Original M6NP cohort
Failed PUL[a]	461 (43)	109 (55)	785 (54)
Age (Years)[b]	31 (27; 35)	30 (7)	32 (27; 36)
First hCG value (IU/L)[b]	334 (120; 1078)	N/A[c]	308 (104; 975)
Second hCG value (IU/L)[b]	158 (53; 460)	N/A[c]	143 (48; 397)
hCG ratio[b]	0.42 (0.27; 0.64)	0.35 (0.27)	0.40 (0.28; 0.58)
hCG average (IU/L)[b]	261 (93; 790)	89 (216)	N/A[d]
Intrauterine pregnancy[a]	362 (34)	76 (39)	501 (35)
Age (Years)[b]	29 (24; 33)	30 (6)	30 (24; 33)
First hCG value (IU/L)[b]	1521 (464; 5125)	N/A[c]	488 (253; 887)
Second hCG value (IU/L)[b]	2765 (983; 7900)	N/A[c]	1061 (515; 1863)
hCG ratio[b]	1.81 (1.39; 2.21)	2.12 (0.57)	2.15 (1.83; 2.50)
hCG average (IU/L)[b]	2226 (746; 6750)	727 (986)	N/A[d]
Ectopic pregnancy[a]	238 (22)	12 (6)	163 (11)
Age (Years)[b]	32 (27; 36)	32 (6.5)	32 (28; 35)
First hCG value (IU/L)[b]	810 (252; 2315)	N/A[c]	521 (200; 1220)
Second hCG value (IU/L)[b]	920 (265; 2735)	N/A[c]	604 (224; 1266)
hCG ratio[b]	1.05 (0.86; 1.31)	1.41 (0.47)	1.16 (0.96; 1.48)
hCG average (IU/L)[b]	865 (270; 2600)	621 (354)	N/A[d]

PUL, pregnancy of unknown location; hCG, human chorionic gonadotropin; Q1, first quartile; Q3, third quartile; IQR, interquartile range; N/A, not available.

[a]Reported as number (%)

[b]Reported as median (Q1; Q3) in the validation cohort and the original M6 cohort, and as median (IQR) in the original M4 cohort.

[c]The first and second hCG value were only presented as an average in the original M4 cohort.

[d]The average hCG value was not presented in the original M6 cohort.

For peer review only

Linear predictor functions for M4 where IUP was reference category [1]:

$$z_1 = log\left(\frac{P(FPUL)}{P(IUP)}\right)$$

$$= +5.8803$$

$$-5.5594 \times (hCG\ ratio - 1.17)$$

$$+2.0504 \times (hCG\ ratio - 1.17)^2$$

$$-1.175 \times \log(average\ hCG)$$

$$z_2 = log\left(\frac{P(EP)}{P(IUP)}\right)$$

$$= 0.3857$$

$$-0.2627 \times (hCG\ ratio - 1.17)$$

$$-3.9319 \times (hCG\ ratio - 1.17)^2$$

$$-0.0572 \times \log(average\ hCG)$$

Linear predictor functions for M6NP where EP was reference category [2]:

$$z_1 = log\left(\frac{P(FPUL)}{P(EP)}\right)$$

$$= 2.5506$$

$$-0.4242 \times log(initial\ hCG)$$

$$-2.9502 \times log(hCG\ ratio)$$

$$+2.1765 \times [log(hCG\ ratio)]^2$$

$$z_2 = log\left(\frac{P(IUP)}{P(EP)}\right)$$

$$= -3.2842$$

$$+0.4072 \times log(initial\ hCG)$$

$$+1.9238 \times log(hCG\ ratio)$$

$$+2.8952 \times [log(hCG\ ratio)]^2$$

For peer review only

By using the exponentiated linear predictor functions of M4 and M6NP respectively the probability of each outcome are computed as:

$$P(FPUL) = \frac{exp(z_1)}{1 + exp(z_1) + exp(z_2)}$$

$$P(IUPor\ EP\ ) = \frac{exp(z_2)}{1 + exp(z_1) + exp(z_2)}$$

$$P(EPor\ IUP) = \frac{1}{1 + exp(z_1) + exp(z_2)}$$

The probability of IUP is computed with the third formula when M4 is used. If M6NP is used the third formula computes the probability of EP. Log in the linear predictor functions refers to the natural logarithm. PUL, pregnancy of unknown location; FPUL, failed PUL; IUP, intrauterin pregnancy, EP, ectopic pregnancy.

Equation of Net Benefit [3]:

$\text{Net benefit} = (\text{True positive})/n \ - \ (\text{False positive})/n \cdot ((P_t/(1-P_t))$, where true positive is the number of EP classified as high risk; false positive is the number of non-EP classified as high risk and n is the total number of patients. In the equation false positives are weighed by the odds of the selected risk threshold (P_t) for high risk classification. If a 5% risk threshold is chosen, a false positive non-EP is valued at 1/19th of a true positive EP.

References:

1. Condous G, Van Calster B, Kirk E, Haider Z, Timmerman D, Van Huffel S, et al. Prediction of ectopic pregnancy in women with a pregnancy of unknown location. Ultrasound Obstet Gynecol 2007; 29: 680-7.
2. Van Calster B, Bobdiwala S, Guha S, Van Hoorde K, Al-Memar M, Harvey R, et al. Managing pregnancy of unknown location based on initial serum progesterone and serial serum hCG levels: development and validation of a two-step triage protocol. Ultrasound Obstet Gynecol 2016; 48: 642-49.
3. Steyerberg EW, Vickers AJ, Cook NR, Gerds T, Gonen M, Obuchowski N, et al. Assessing the performance of prediction models: a framework for traditional and novel measures. Epidemiology 2010; 21: 128-38.

Supplemental Table 3. Descriptive statistics of 12 ectopic pregnancies misclassified as low risk by M6NP.						
Type of PUL	First hCG (IU/L)	Second hCG (IU/L)	M6NP prediction	Site of EP	Rupture	Treatment
True PUL	470	290	FPUL	Tubal	No	Salpingotomy
Probable EP	88	42	FPUL	Tubal	Yes	Salpingectomy
True PUL	619	1948	IUP	Tubal	No	Salpingectomy
True PUL	3700	1500	FPUL	Tubal	No	Salpingectomy
True PUL	700	220	FPUL	Tubal	No	Salpingectomy
Probable EP	420	210	FPUL	Tubal	No	Salpingectomy
True PUL	10000	20000	IUP	Tubal	No	Salpingectomy
True PUL	640	240	FPUL	Tubal	No	Salpingectomy
True PUL	43	170	IUP	Tubal	No	Salpingectomy
Probable EP	56	23	FPUL	Tubal	No	Salpingectomy
True PUL	130	330	IUP	Tubal	No	salpingectomy
True PUL	230	590	IUP	Tubal	No	Salpingectomy
hCG, human chorionic gonadotropin, PUL, pregnancy of unknown location; EP, ectopic pregnancy; FPUL, failed PUL; IUP, intrauterine pregnancy.						

For peer review only

Supplemental Table 4. Descriptive statistics of patients in the sensitivity analysis (n=1186).	
Age (years)	
Median (Q1;Q3)	31 (26;35)
Range	15–49
First hCG value (IU/L)	
Median (Q1;Q3)	697 (210;2300)
Range	7–180000
Second hCG value (IU/L)	
Median (Q1;Q3)	615 (142;2655)
Range	1–190000
Hours between two hCG samples, n (%)	
<24	70 (6)
24–39	141 (12)
40–56	751 (63)
57–72	169 (14)
>72	55 (5)
Type of PUL, n (%)	
True PUL	880 (74)
Probable IUP	254 (21)
Probable EP	52 (4)
Vaginal bleeding, n (%)	803 (68)
Prior ectopic pregnancy, n (%)	86 (7)
IUD, n (%)	37 (3)

hCG, human chorionic gonadotropin; Q1, first interquartile; Q3, third interquartile; PUL, pregnancy of unknown location; IUP, intrauterine pregnancy, EP, ectopic pregnancy; IUD, intrauterine device. Because of rounding the summarised percentage sometimes are not exactly 100%.

For peer review only

Supplemental Table 5. Performance of M6NP and M4 at the 5% threshold in the sensitivity analysis.		
Performance measure	M6NP	M4
AUC	0.84 (0.81–0.86)	0.80 (0.77–0.83)
Sensitivity, %	93 (90–96)	83 (78–87)
False positive rate, %	49 (48–53)	36 (33–39)
Negative predictive value, %	96 (94–98)	92 (90–94)
AUC, area under the receiver operating characteristic curve. 95% confidence interval (in parentheses).		

For peer review only

Reporting checklist for prediction model development/validation.

Based on the TRIPOD guidelines.

Instructions to authors

Complete this checklist by entering the page numbers from your manuscript where readers will find each of the items listed below.

Your article may not currently address all the items on the checklist. Please modify your text to include the missing information. If you are certain that an item does not apply, please write "n/a" and provide a short explanation.

Upload your completed checklist as an extra file when you submit to a journal.

In your methods section, say that you used the TRIPODreporting guidelines, and cite them as:

Collins GS, Reitsma JB, Altman DG, Moons KG. Transparent reporting of a multivariable prediction model for individual prognosis or diagnosis (TRIPOD): The TRIPOD statement.

		Reporting Item	Page Number
Title			
	#1	Identify the study as developing and / or validating a multivariable prediction model, the target population, and the outcome to be predicted.	1
Abstract			
	#2	Provide a summary of objectives, study design, setting, participants, sample size, predictors, outcome, statistical analysis, results, and conclusions.	1
Introduction			
	#3a	Explain the medical context (including whether diagnostic or prognostic) and rationale for developing or validating the multivariable prediction model, including references to existing models.	3
	#3b	Specify the objectives, including whether the study describes the development or validation of the model or both.	4

For peer review only

Methods

Source of data	#4a	Describe the study design or source of data (e.g., randomized trial, cohort, or registry data), separately for the development and validation data sets, if applicable.	4
Source of data	#4b	Specify the key study dates, including start of accrual; end of accrual; and, if applicable, end of follow-up.	4
Participants	#5a	Specify key elements of the study setting (e.g., primary care, secondary care, general population) including number and location of centres.	4
Participants	#5b	Describe eligibility criteria for participants.	5
Participants	#5c	Give details of treatments received, if relevant	n/a, no treatment was given
Outcome	#6a	Clearly define the outcome that is predicted by the prediction model, including how and when assessed.	6
Outcome	#6b	Report any actions to blind assessment of the outcome to be predicted.	n/a, predictions and outcome assessment were done separately
Predictors	#7a	Clearly define all predictors used in developing or validating the multivariable prediction model, including how and when they were measured	6 and 7
Predictors	#7b	Report any actions to blind assessment of predictors for the outcome and other predictors.	n/a, predictions and outcome assessment were done separately
Sample size	#8	Explain how the study size was arrived at.	7
Missing data	#9	Describe how missing data were handled (e.g., complete-case analysis, single imputation, multiple imputation) with details of any imputation method.	9
Statistical analysis methods	#10a	If you are developing a prediction model describe how predictors were handled in the analyses.	n/a, validation study
Statistical analysis methods	#10b	If you are developing a prediction model, specify type of model, all model-building procedures (including any predictor selection), and method for internal validation.	n/a

For peer review only

Statistical analysis methods	#10c	If you are validating a prediction model, describe how the predictions were calculated.	7
Statistical analysis methods	#10d	Specify all measures used to assess model performance and, if relevant, to compare multiple models.	9
Statistical analysis methods	#10e	If you are validating a prediction model, describe any model updating (e.g., recalibration) arising from the validation, if done	n/a
Risk groups	#11	Provide details on how risk groups were created, if done.	n/a
Development vs. validation	#12	For validation, identify any differences from the development data in setting, eligibility criteria, outcome, and predictors.	5
Results			
Participants	#13a	Describe the flow of participants through the study, including the number of participants with and without the outcome and, if applicable, a summary of the follow-up time. A diagram may be helpful.	10
Participants	#13b	Describe the characteristics of the participants (basic demographics, clinical features, available predictors), including the number of participants with missing data for predictors and outcome.	10
Participants	#13c	For validation, show a comparison with the development data of the distribution of important variables (demographics, predictors and outcome).	10
Model development	#14a	If developing a model, specify the number of participants and outcome events in each analysis.	n/a
Model development	#14b	If developing a model, report the unadjusted association, if calculated between each candidate predictor and outcome.	n/a
Model specification	#15a	If developing a model, present the full prediction model to allow predictions for individuals (i.e., all regression coefficients, and model intercept or baseline survival at a given time point).	n/a
Model specification	#15b	If developing a prediction model, explain how to the use it.	n/a

For peer review only

Model performance	#16	Report performance measures (with CIs) for the prediction model.	11 and 12
Model-updating	#17	If validating a model, report the results from any model updating, if done (i.e., model specification, model performance).	n/a, updating not performed
Discussion			
Limitations	#18	Discuss any limitations of the study (such as nonrepresentative sample, few events per predictor, missing data).	13
Interpretation	#19a	For validation, discuss the results with reference to performance in the development data, and any other validation data	12
Interpretation	#19b	Give an overall interpretation of the results, considering objectives, limitations, results from similar studies, and other relevant evidence.	13
Implications	#20	Discuss the potential clinical use of the model and implications for future research	13 and 14
Other information			
Supplementary information	#21	Provide information about the availability of supplementary resources, such as study protocol, Web calculator, and data sets.	14 and 15
Funding	#22	Give the source of funding and the role of the funders for the present study.	15

None The TRIPOD checklist is distributed under the terms of the Creative Commons Attribution License CC-BY. This checklist can be completed online using https://www.goodreports.org/, a tool made by the EQUATOR Network in collaboration with Penelope.ai

For peer review only

Pregnancy of unknown location: external validation of the hCG based M6NP and M4 prediction models in an emergency gynaecology unit.

Journal:	*BMJ Open*
Manuscript ID	bmjopen-2021-058454.R2
Article Type:	Original research
Date Submitted by the Author:	25-Jun-2022
Complete List of Authors:	Fistouris, Johan; University of Gothenburg Institute of Clinical Sciences, Obstetrics and Gynaecology<br>Bergh, Christina; University of Gothenburg Institute of Clinical Sciences, Obstetrics and Gynaecology<br>Strandell, Annika; University of Gothenburg Institute of Clinical Sciences
**Primary Subject Heading**:	Obstetrics and gynaecology
Secondary Subject Heading:	Obstetrics and gynaecology
Keywords:	ULTRASONOGRAPHY, GYNAECOLOGY, Ultrasound < RADIOLOGY & IMAGING

SCHOLARONE™
Manuscripts

I, the Submitting Author has the right to grant and does grant on behalf of all authors of the Work (as defined in the below author licence), an exclusive licence and/or a non-exclusive licence for contributions from authors who are: i) UK Crown employees; ii) where BMJ has agreed a CC-BY licence shall apply, and/or iii) in accordance with the terms applicable for US Federal Government officers or employees acting as part of their official duties; on a worldwide, perpetual, irrevocable, royalty-free basis to BMJ Publishing Group Ltd ("BMJ") its licensees and where the relevant Journal is co-owned by BMJ to the co-owners of the Journal, to publish the Work in this journal and any other BMJ products and to exploit all rights, as set out in our licence.

The Submitting Author accepts and understands that any supply made under these terms is made by BMJ to the Submitting Author unless you are acting as an employee on behalf of your employer or a postgraduate student of an affiliated institution which is paying any applicable article publishing charge ("APC") for Open Access articles. Where the Submitting Author wishes to make the Work available on an Open Access basis (and intends to pay the relevant APC), the terms of reuse of such Open Access shall be governed by a Creative Commons licence – details of these licences and which Creative Commons licence will apply to this Work are set out in our licence referred to above.

Other than as permitted in any relevant BMJ Author's Self Archiving Policies, I confirm this Work has not been accepted for publication elsewhere, is not being considered for publication elsewhere and does not duplicate material already published. I confirm all authors consent to publication of this Work and authorise the granting of this licence.

For peer review only

Title: Pregnancy of unknown location: external validation of the hCG based M6NP and M4 prediction models in an emergency gynaecology unit.

Author names and affiliations: Johan Fistouris[a], Christina Bergh[a] and Annika Strandell[a]

[a]University of Gothenburg Institute of Clinical Sciences, Department of Obstetrics and Gynaecology, Sahlgrenska University Hospital, Gothenburg, Sweden

Correspondence to: Dr Johan Fistouris, Department of Gynaecology and Reproductive Medicine, Sahlgrenska University Hospital, SE-413 45 Gothenburg, Sweden. (E-mail: johan.fistouris@vgregion.se). Telephone: 0046-31-707552678

Keywords: Chorionic Gonadotropin, Ectopic pregnancy, Pregnancy of Unknown Location, Statistical Models

Word count: 3970

For peer review only

ABSTRACT

Objective: To investigate if M6NP predicting ectopic pregnancy (EP) among women with pregnancy of unknown location (PUL) is valid in an emergency gynaecology setting and comparing it with its predecessor M4.

Design: Retrospective cohort study.

Setting: University hospital

Participants: Women with PUL.

Methods: All consecutive women with a PUL during a study period of 3 years were screened for inclusion. Risk prediction of an EP was based on two serum human chorionic (hCG) levels taken at least 24 hours and no longer than 72 hours apart.

Main Outcome Measures: The area under the ROC curve (AUC) and calibration assessed the predictive performance. The proportion of ectopic and non-ectopic pregnancies classified as high risk assessed the model's sensitivity and false positive rate (FPR). The proportion of non-ectopic pregnancies among women classified as low risk was the models negative predictive value (NPV). The clinical utility of a model was evaluated with decision curve analysis.

Results: 1061 women were included in the study, of which 238 (22%) had a final diagnosis of EP. The AUC for EP was 0.85 for M6NP and 0.81 for M4. M6NP made accurate risk predictions of EP up to predictions of 20% but thereafter risk were underestimated. M4 was poorly calibrated up to risk predictions of 40%. With a 5% threshold for high risk classification the sensitivity for EP was 95% for M6NP, the FPR 50% and NPV 97%. M6NP had

For peer review only

higher sensitivity and NPV than M4 but also a higher FPR. M6NP had utility at all thresholds as opposed to M4 that had no utility at thresholds ≤5%.

Conclusions: M6NP had better predictive performance than M4 and is valid in women with PUL attending an emergency gynaecology unit. Our results can encourage implementation of M6NP in related yet untested clinical settings to effectively support clinical decision making.

Strengths and limitations of this study

- This is the first study to test the transportability (generalisability) of the M6NP prediction model to an emergency gynaecology setting, originally developed for early pregnancy assessment units in the UK.
- The rate of ectopic pregnancy was twice as high in comparison with the original M6NP cohort and we also included inpatients contributing to a different case mix.
- A limitation was the retrospective study design, although common for validation studies.
- Another limitation was the complete case analysis; however a sensitivity analysis adding all women regardless of time interval between two hCG showed consistent performance for M6NP.

For peer review only

INTRODUCTION

To reduce maternal complications from ectopic pregnancy (EP), an early diagnosis using transvaginal ultrasound (TVUS) is essential [1]. Between 30 and 40 % of EP remain undiagnosed after the first examination of women presenting with pain or bleeding in early pregnancy and will be managed as a pregnancy of unknown location (PUL) until a final diagnosis is established [2]. The reported rupture rate of EP among women with a PUL range from 0.03 to 2.4% and sometimes necessitates blood transfusion [3-6]. A key concept during follow-up is timely diagnosis and treatment of an EP while avoiding unnecessary visits for the remaining majority of women with PUL that eventually will be diagnosed with a intrauterin pregnancy (IUP) or a spontaneously resolving PUL (failed PUL) [7]. The change in serum human chorionic gonadotropin (hCG) levels between two samples measured 48 hours apart indicates if a woman is at high or low risk of having an EP and decisive of planning follow- up visits [8]. In a systematic review and meta-analysis the widely reported logistic regression model "M4" that is based on two hCG had the best performance predicting the presence of an EP among women with PUL [9-12].

M4 was developed in an early pregnancy assessment unit (EPAU) in London and has mostly been evaluated in similar clinical settings within that geographical region [10]. The M4 model was updated in a larger cohort of women with PUL in 2016 with an option to include serum progesterone as a predictor (M6P) or not (M6NP) [13]. Recent research suggests that both M6P and M6NP are superior to M4 when compared in EPAUs in the UK [14]. The performance of a prediction model is influenced by the characteristics of the underlying population and may not be generalizable to a different clinical context in another country with the risk of being harmful if implemented [15]. For instance when M4 was tested in a US

For peer review only

emergency department the sensitivity for identifying an EP was only 55% [16]. M6NP and M6P have not been validated in an emergency gynaecology unit.

The primary aim of the present study was to externally validate M6NP in an emergency gynaecology unit and compare the performance to its predecessor M4.

MATERIALS AND METHODS

Design and Setting

External validation was performed on women with PUL attending the emergency gynaecology unit at the Sahlgrenska University Hospital from January 1st, 2011 to December 31st, 2013. The emergency gynaecology unit serves patients with various gynaecological problems 24 hours a day, 7 days a week either self-referred or referred from other clinics in the greater Gothenburg area.

The initial examination of women with early pregnancy complaints was performed by physicians in early stage of specialist training and a consultant gynaecologist assisted upon request. Women were monitored with serial measurement of hCG levels whenever the physician was uncertain about the pregnancy location. The second blood sample for hCG measurement was routinely scheduled between two and three days after the first hCG. Interpretation of hCG development was not based on any specific percentage change but approximations of the doubling time in hCG for a viable IUP and the expected hCG decline for a miscarriage [17]. Our follow-up routine for outpatients' after two hCG measurements has been described in a prior study from our unit [6]. Outpatients with deteriorating symptoms were instructed to re- visit the unit. Depending on the severity of symptoms and

For peer review only

clinical findings, inpatient evaluation and further investigation by a senior physician was considered.

The study hospital was the only referral site for treatment of EP in the area as opposed to other early pregnancy complications. Laparoscopic intervention was the first line of treatment for EP during the study period. Diagnostic laparoscopy was also performed in patients with a prolonged period of follow-up where hCG levels did not decline and there was no evidence of an IUP. In cases where laparoscopy failed to identify an EP uterine evacuation followed to establish a final diagnosis. Methotrexate was used for persistent trophoblast after initial surgery or as treatment of interstitial or caesarean scar pregnancies.

Participants

Women presenting to the emergency gynaecology unit with complaints in early pregnancy, deemed to have a PUL were screened for inclusion. All women with a PUL and at least two hCG levels were eligible for inclusion into the study. Also, women that were initially managed as outpatients but the second hCG was taken during or after a hospital stay (inpatients) were included unlike prior studies.

In the original M6NP study the second hCG level was imputed when taken more than 3 days after the first hCG [13]. However, in clinical practice the second hCG has only been used for prediction if taken 48±8 hours after the first [3]. We decided to include women with two hCG taken at least 24 hours and no longer than 72 hours apart, to assure that the validation cohort represented the actual PUL management in our clinic. We excluded women where the final outcome of PUL was unknown (lost to follow-up).

For peer review only

Data Collection

The following variables were obtained from the patients electronical medical record: Age at the first visit; symptoms related to the first visit (i.e. pain or bleeding); prior EP or use of a contraceptive intrauterine device; level of the first and second serum hCG (IU/L); date and time of hCG measurement; type of PUL and the final outcome of the pregnancy. All serum hCG levels were analysed in the same laboratory.

We defined three types of a PUL based on TVUS at the first visit or during follow-up [18]: no signs of a pregnancy neither inside nor outside of the uterus defined a true PUL; when an intrauterine sac-like structure or suspicion of retained products of conception was seen it was defined as a probable IUP; probable EP was used when an ectopic sac-like or solid structure was seen [19]. A gestational sac containing a yolk sac with or without an embryo seen with TVUS defined a definite IUP or EP and therefore not a PUL [18].

Prediction models

M4 and M6NP use polynomial logistic regression to compute probabilities for each of the three possible outcomes of a PUL: failed PUL, IUP and EP. The covariates of both models are based on two hCG levels analysed in serum (Supplemental Table 1).

We used the original equations (Supplemental) of M6NP and M4 to compute probabilities as recommended instead of refitting the model [15, 20]. M6P was not validated since serum progesterone is not used in our unit when managing PUL.

For peer review only

Reference Standard

The reference standard was based on the combination of hCG, TVUS findings, and surgical procedures. There are four categories of PUL outcome and these were set as reference standard: (1) A failed PUL was defined by spontaneously declining hCG levels below 0.6 IU/L or a negative pregnancy test during follow-up where nor an IUP or EP could be confirmed. (2) An IUP was diagnosed by either TVUS; histological findings of chorionic villi after vacuum aspiration; completion of a suspected miscarriage after medical or surgical treatment verified by TVUS or decline of hCG levels <0.6 IU/L. (3) An EP was diagnosed with TVUS, at laparoscopy or hysteroscopy with or without confirmed histology. (4) A persisting PUL (PPUL) was defined when no pregnancy was visible on TVUS and hCG levels plateaued (at least three consecutive hCG with a change <15% between measurments during a one week follow-up). If no chorionic villi were present in uterine aspiration specimen or none was analysed and hCG levels remained elevated also defined a PPUL regardless of prior hCG development as were non-visualised pregnancies treated with methotrexate

In the analysis PPUL were incorporated among EP as in previous studies [4, 11, 13]. We used the same definitions as in the original M4 and M6NP studies and a consensus statement [10, 13, 18].

The TRIPOD (Transparent Reporting of a multivariable for individual Prognosis or Diagnosis) guidelines were followed conducting this study [21].

For peer review only

Study Size

A validation cohort should contain at least 100 events and ideally 200 events to adequately evaluate prediction rules [22]. Our validation cohort contained 238 EP satisfying this requirement.

Patient and public involvement statement

Patients and the public were not involved in the development of the research question.

Statistical Analysis

The predictive performance of a model was evaluated with discrimination and calibration [23]. We focused on the dichotomous outcome of EP and non-EP (IUP and failed PUL) as being the most important to distinguish in clinical practice. Discrimination refers to how well the predicted probabilities of a model differentiates between EP and non-EP and was expressed as the area under the ROC curve (AUC).

Calibration describes the agreement between the predicted risk of an EP and the observed risk (proportion) of EP [23]. Calibration was assessed by calibration plots, calibration-in-the large and calibration slope [24]. The calibration plot shows prediction accuracy for each patient individually with a computed loess smoothing curve [25]. Calibration-in-the-large evaluates the average predicted risk of EP in the entire cohort compared with actual rate of EP while calibration slope evaluates potential under- or overfitting of the risk predictions. If the calibration-in-the large is >0 the average predicted risk of EP is lower than the actual proportion of EP in the cohort, and if the calibration-in-the large is <0 the average predicted risk is higher. A calibration slope < 1 indicates that (on average) high risk predictions are overestimated and low risk predictions are underestimated. A slope > 1 indicates that (on

For peer review only

average) high risk predictions are underestimated and low risk predictions are overestimated [26].

Classification accuracy was measured with sensitivity (proportion of EP classified as high risk), false positive rate (FPR), (proportion of non-EP classified as high risk) and the negative predictive value (proportion of non-EP among women classified as low risk) as it was presented in the original M6NP study [13]. In implementation studies from EPAU in the UK a PUL has been classified as high risk of EP if the predicted probability of EP reached a threshold (cut-off) of 5% [14, 27]. An optimal threshold can vary between clinical settings; we therefore explored other plausible thresholds (2.5, 5, and 10%) for classification as recommended when validating prediction models [28].

To determine which model that would lead to the best clinical outcomes (clinical utility) if used for clinical decision making the Net Benefit (NB) was measured at the 5% threshold and over a range of thresholds in a decision curve [29]. The NB is calculated as the difference between the number of true positives (correctly identified EP) and false positives (misclassified non-EP) where the latter is weighed by the probability threshold (Supplemental). The choice of a certain threshold for classification reflects how the clinician values a true positive in relation to a false positive. A lower threshold implies that a true positive is valued higher than if a higher threshold is used. For instance, the 5% (odds 1:19) threshold translates to an acceptance of 19 false positives per correctly identified EP. The models´ NB was compared with default strategies of assuming all as positive (high risk of EP) and none as positive in a decision curve. If a model has a NB below a default strategy at a specific threshold it has no clinical utility at that threshold.

For peer review only

Since we used a complete case analysis for the validation cohort we performed a sensitivity analysis to test if the exclusion of women with a sampling interval between two hCG shorter than 24 hours or longer than 72 hours presented a risk for introducing bias [30]. The sensitivity analysis thus included all eligible women regardless of hCG sampling interval.

Performance measures are presented with 95% confidence intervals (CI). McNemar´s test was used for testing differences of paired proportions. DeLong's test was used for testing the difference in AUC. A p-value of < 0.05 was considered statistically significant.

Statistical analyses were performed using SPSS 21.0 (SPSS, Chicago, IL, USA) and SAS version 9.4 (Cary, NC, USA).

RESULTS

A total of 1447 patients were screened for inclusion into the study. 239 patients had only one hCG sample of which 186 had their pregnancy located (134 EP and 46 IUP) before a second hCG was taken (Fig. 1). Another 33 patients did not have a second hCG since a miscarriage was suspected, although not confirmed, and a urine pregnancy test was used as follow-up. Only 21 patients were lost to follow-up. All 239 patients with a missing second hCG were managed as non-eligible (Fig. 1). Descriptive statistics of these women are presented in Supplemental Table 2.

There were 1208 eligible women. However, 125 (10%) of these had a second hCG outside the targeted time frame. Although excluded from the validation cohort they were part of the sensitivity analysis (Fig. 1). Six women were diagnosed with a gestational trophoblastic disease and were not part of the validation cohort (Fig. 1). The validation cohort consisted of 1061 women. Descriptive statistics of the validation cohort and women that were eligible

For peer review only

but lost to follow-up (n=15) are presented in Table 1. In the validation cohort 71% of women (751/1061) had an hCG sampling interval of 48±8 hours. The clinical picture warranted inpatient surveillance before a second hCG was taken for 8% of the women (89/1061). Final outcomes of PUL were 461 failed PUL, 362 IUP of which 98 were non-viable and 238 EP (22%) (including 46 PPUL) (Supplemental Table 3).

Supplemental Table 3 includes data from the original M4 and M6NP cohorts where EP rates were 6% and 11% respectively. The median of the first hCG level was numerically higher and the median hCG ratio was lower for EP in our validation cohort as compared with EP in the original M6NP cohort (Supplemental Table 3). Also, for IUP the median of the first hCG level was numerically higher but the median hCG ratio was lower in our cohort. HCG characteristics for failed PUL appear to be more similar between our cohort and the original M6NP cohort.

Discrimination and Calibration

The AUC for EP was higher for M6NP, 0.85 (95% CI 0.82–0.88) than for M4, 0.81 (95% CI 0.78–0.84) (p<0.01). As seen in the calibration plot, M6NP was well calibrated up to predictions of 0.20 with the curve close to the diagonal line, but underestimated risk thereafter (Fig. 2A). M4 made poorly calibrated risk predictions of EP up to 0.40 (Fig. 2B). Calibration- in- the-large showed that the average predicted risk of EP by both models was underestimated, especially M4 as it was further from 0 as compared with M6NP (Fig. 2). The average predicted risk by M4 among higher risk groups was overestimated and underestimated among lower risk groups as indicated by the calibration slope being < 1, the slope was below but closer to 1 for M6NP (Fig. 2).

For peer review only

Classification and Clinical Utility

At the 5% risk threshold M6NP had higher sensitivity for identifying an EP, 95% (95% CI 92–98) than M4, 85% (95% CI 81–90) (p<0.01) as presented in Table 2. The FPR was 37% for M4 and 50% for M6NP (p<0.01). The negative predictive value (NPV) for M4 was 94% and 97% for M6NP (p<0.01). M4 and M6NP classified 52% (95% CI: 50–55) and 40% (95% CI: 37–42) of PUL as low risk respectively (p<0.01). Less than 50% of failed PUL and 75% of IUP rendered risk estimates ≥ 5% of an EP by M6NP (Supplemental Fig. 1). This equals the proportion of failed PUL and IUP being misclassified as EP by M6NP. M6NP misclassified twelve EP as low risk of which seven were predicted to have a failed PUL. Among these EP, one had emergency surgery due to rupture during follow-up, but no blood transfusion was needed. Supplemental Table 4 presents details for all EP misclassified by M6NP.

At the 10% threshold M6NP had a sensitivity of 91%, FPR of 39% and NPV of 96% (Table 2). The sensitivity was lower at 80% for M4 and the FPR was improved but not as marked as for M6NP. At the 2.5% threshold as compared with the 5% threshold the sensitivity and NPV were only marginally higher for M6NP while the FPR increased from 50 to 61% and the proportion of PUL classified as low risk decreased from 40 to 31% (Table 2).

Decision curve analysis showed that the curve for M6NP was above that of M4 and the default strategy of assuming all as positive over the entire range of thresholds (Fig. 3). This means that M6NP had higher NB than M4 and utility at any threshold. M4 had no utility at risk thresholds ≤5% as the curve was under the reference curve of assuming all women as positive.

For peer review only

Sensitivity Analysis

In the sensitivity analysis all women with at least two hCG and any sampling interval were included. Descriptive statistics for all 1186 women were similar with those in the validation cohort except the hCG sampling interval (Supplemental Table 5). Both models had marginally lower AUC (Supplemental Table 3). Calibration for both models was little changed as seen in the calibrations plots (Supplemental Fig. 2). The sensitivity and the FPR at the 5% risk threshold were almost the same as in the validation cohort for both models, although slightly lower (Supplemental Table 6).

DISCUSSION

This external validation study found that M6NP perform similar in a Swedish emergency gynaecology unit as in EPAU in the UK where it was developed. M6NP made more accurate predictions of an EP and could better differentiate an EP from a non-EP than M4. A difference between the models in a clinical perspective was that M6NP classified fewer PUL as low risk than M4 leading to its higher sensitivity and false positive rate at the 5% threshold. This could result in more women being part of some sort of close monitoring if clinical decision making is based on M6NP as compared with M4, but importantly an opportunity for early diagnosis of more EP starting as PUL. At the 10% threshold M6NP maintained a high sensitivity while the FPR was significantly lower. M6NP had clinical utility at any threshold unlike M4 that had no clinical utility at thresholds ≤5%.

This is the first study to test the generalisability of M6NP in an emergency gynaecology unit which is an important step before a prediction model can be recommended for, or discouraged from wider implementation [31]. We present real world data on how M6NP

For peer review only

would perform in a setting that in several aspects are different from the original cohort with encouraging results.

One limitation of the study was the complete case analysis approach. However, a sensitivity analysis that included all women regardless of sampling interval between two hCG revealed that the performance of M6NP was sparsely affected. We have no reason to believe that the validation cohort is not representative of the target population and the sample size after exclusion was still large enough for inference about the models' predictive performance [21]. We refrained from including patients lost to follow-up in the sensitivity analysis which could be a limitation of the study. However, given the small fraction of patients lost to follow-up, a sensitivity analysis using multiple imputation would produce virtually identical results as the current analysis.

Six eligible women with a gestational trophoblastic disease were excluded. In a clinical situation a diagnosis is not known at the moment of prediction, why it might have been ideal to include these women. A post hoc analysis revealed that 5 out of 6 would have been classified as high risk, affecting specificity negatively since being non-EP. Although a limited number, this must be taken into consideration when interpreting our data. Another limitation was the retrospective design of the study although common when validating or developing prediction models as in the original M6NP study [15].

The average predicted probability of EP in the entire cohort (calibration in the large) was underestimated, especially for M4, which was an effect of the lower rate of EP in the original cohorts [10, 13]. M6NP was well calibrated up to predictions of 0.20 contrary to M4. Instead predictions by M4 were clearly too low up to predictions of 0.40, also reported when recently validated in EPAUs in the UK [14]. This implies that predictions made by M6NP

For peer review only

unlike M4 were accurate at thresholds relevant for clinical decision making and underestimation at higher thresholds may thus be irrelevant in this respect [23].

M6NP mainly misclassified IUP and to a lesser extent failed PUL as EP in our cohort, consistent with the findings in the UK validation study [14]. M6NP generated higher risk estimates of an EP for failed PUL in our cohort as compared to failed PUL in the validation study from the UK. A greater number of failed PUL was consequently misclassified as high risk at the 5% threshold leading to a higher FPR in our study than in previous studies [13, 14]. The reasons for discrepancies between risk predictions could be because of differences in hCG sampling interval, inclusion of inpatients and that the median hCG levels in our study were higher. The use of imputed hCG values when the second hCG was taken after more than 3 days or missing completely in the UK validation study could also contribute to differences [14].

We did not assess M6P (adding progesterone as a predictor) since progesterone is not used in our clinic. As previously reported in studies from the UK and Australia it is probable that M6P would perform better as compared with M6NP also in our unit [14, 32].

In clinical practice a woman with a predicted failed PUL after just two hCG measurments is followed up with a home urine pregnancy test after two weeks and if an IUP is predicted examination is repeated after one week. Women with a PUL classified as high risk of EP are re-examined within 24 hours [3, 33]. In such clinical situation high classification accuracy is crucial for a prediction model to be useful. In our study M6NP only misclassified 5% (n=12) of EP as low risk. On the other hand, 50% of non-EP were misclassified as high risk which could result in high numbers of unnecessary visits, admissions to the ward and potentially interventions if implemented. There is insufficient data to rely on when it comes to costs and

For peer review only

benefits of misclassifying women with a PUL which makes it difficult to define an optimal threshold for high risk classification, a general challenge when implementing a clinical prediction model [28]. When M4 and M6NP have been used in clinical practice it has been considered reasonable to allow 19 misclassified non-EP for every correctly classified EP which equals the 5% risk threshold [3, 12]. Our population had a high rate of EP and the 5% threshold therefore seems justifiable to optimize sensitivity. However, the sensitivity for EP remained high for M6NP at the 10% threshold while the FPR decreased from 50 to 39%. The trade-off between sensitivity and FPR at the 2.5% threshold was less favourable since the FPR increased significantly and the sensitivity was only marginally higher as compared with the 5% threshold. Since M6NP had clinical utility at the 5 and 10% thresholds we suggest that any of these are appropriate to use in our clinical setting.

Regardless which threshold that is finally implemented one must remember that M6NP should always be used as a compliment to clinical judgement and never as a sole decision instrument. M6NP and M6P are available at www.earlypregnancycare.co.uk where the 5% threshold is used by default.

CONCLUSION

The performance of M6NP as opposed to M4 was encouraging when validated on women with PUL in an emergency gynaecology unit. M6NP can be introduced in related yet untested clinical settings outside the UK and EPAU to effectively support clinical decision making. Updating of M6NP in a local context to account for geographical differences could be an option to improve calibration.

Contributors J.F. and A.S. contributed substantially to conception and design of the study. J.F collected the data. J.F performed some of the statistical analysis and J.F and A.S interpreted

For peer review only

the data. J.F. and A.S. wrote the article. J.F drafted the article. C.B. commented on the drafts. All authors made revisions to the article and approved the final version.

Funding This work was supported by grants from the Swedish state under the agreement between the Swedish government and the county councils, the ALF-agreement [ALFGBG-720291, ALFGBG-72150] and The Healthcare Board, Region Västra Götaland.

Competing interests None declared.

Ethics approval statement approval for this study was obtained from the Regional Ethical Review Board, Gothenburg, Sweden (approval # 50115).

Data availability statement Data are available on reasonable request. Requests should be made to the lead author johan.fistouris@vgregion.se.

For peer review only

REFERENCES

1. Elson J, Salim R, Potdar N, Chetty M, Ross JA ,Kirk E. on behalf of the Royal College of Obstetricians and Gynaecologists. Diagnosis and management of ectopic pregnancy. BJOG 2016;.123:e15–e55. BJOG 2016; 123: e15-e55.
2. Crochet J BL. Does ThisWoman Have an Ectopic Pregnancy? The Rational Clinical Examination Systematic Review. JAMA 2013; Vol 309: 1722-29.
3. Bobdiwala S, Christodoulou E, Farren J, Mitchell-Jones N, Kyriacou C, Al-Memar M, et al. Triaging women with pregnancy of unknown location using two-step protocol including M6 model: clinical implementation study. Ultrasound Obstet Gynecol 2020; 55: 105-14.
4. Nadim B, Leonardi M, Infante F, Lattouf I, Reid S ,Condous G. Rationalizing the management of pregnancies of unknown location: Diagnostic accuracy of human chorionic gonadotropin ratio-based decision tree compared with the risk prediction model M4. Acta Obstet Gynecol Scand 2020; 99: 381-90.
5. Morse CB, Sammel MD, Shaunik A, Allen-Taylor L, Oberfoell NL, Takacs P, et al. Performance of human chorionic gonadotropin curves in women at risk for ectopic pregnancy: exceptions to the rules. Fertil Steril 2012; 97: 101-6 e2.
6. Fistouris J, Bergh C ,Strandell A. Classification of pregnancies of unknown location according to four different hCG-based protocols. Hum Reprod 2016; 31: 2203-11.
7. Kirk E, Bottomley C ,Bourne T. Diagnosing ectopic pregnancy and current concepts in the management of pregnancy of unknown location. Hum Reprod Update 2014; 20: 250-61.
8. van Mello NM, Mol F, Opmeer BC, Ankum WM, Barnhart K, Coomarasamy A, et al. Diagnostic value of serum hCG on the outcome of pregnancy of unknown location: a systematic review and meta-analysis. Hum Reprod Update 2012; 18: 603-17.
9. Bobdiwala S, Saso S, Verbakel JY, Al-Memar M, Van Calster B, Timmerman D, et al. Diagnostic protocols for the management of pregnancy of unknown location: a systematic review and meta-analysis. BJOG 2019; 126: 190-98.
10. Condous G, Van Calster B, Kirk E, Haider Z, Timmerman D, Van Huffel S, et al. Prediction of ectopic pregnancy in women with a pregnancy of unknown location. Ultrasound Obstet Gynecol 2007; 29: 680-7.
11. Guha S, Ayim F, Ludlow J, Sayasneh A, Condous G, Kirk E, et al. Triaging pregnancies of unknown location: the performance of protocols based on single serum progesterone or repeated serum hCG levels. Hum Reprod 2014; 29: 938-45.
12. Bobdiwala S, Guha S, Van Calster B, Ayim F, Mitchell-Jones N, Al-Memar M, et al. The clinical performance of the M4 decision support model to triage women with a pregnancy of unknown location as at low or high risk of complications. Hum Reprod 2016; 31: 1425-35.
13. Van Calster B, Bobdiwala S, Guha S, Van Hoorde K, Al-Memar M, Harvey R, et al. Managing pregnancy of unknown location based on initial serum progesterone and serial serum hCG levels: development and validation of a two-step triage protocol. Ultrasound Obstet Gynecol 2016; 48: 642-49.
14. Christodoulou E, Bobdiwala S, Kyriacou C, Farren J, Mitchell-Jones N, Ayim F, et al. External validation of models to predict the outcome of pregnancies of unknown location: a multicentre cohort study. BJOG 2021; 128: 552-62.
15. Moons KG, Kengne AP, Grobbee DE, Royston P, Vergouwe Y, Altman DG, et al. Risk prediction models: II. External validation, model updating, and impact assessment. Heart 2012; 98: 691-8.
16. Barnhart KT, Sammel MD, Appleby D, Rausch M, Molinaro T, Van Calster B, et al. Does a prediction model for pregnancy of unknown location developed in the UK validate on a US population? Hum Reprod 2010; 25: 2434-40.

For peer review only

17. Butts SF, Guo W, Cary MS, Chung K, Takacs P, Sammel MD, et al. Predicting the decline in human chorionic gonadotropin in a resolving pregnancy of unknown location. Obstet Gynecol 2013; 122: 337-43.
18. Barnhart K, van Mello NM, Bourne T, Kirk E, Van Calster B, Bottomley C, et al. Pregnancy of unknown location: a consensus statement of nomenclature, definitions, and outcome. Fertil Steril 2011; 95: 857-66.
19. Pregnancy EwgoE, Kirk E, Ankum P, Jakab A, Le Clef N, Ludwin A, et al. Terminology for describing normally sited and ectopic pregnancies on ultrasound: ESHRE recommendations for good practice. Hum Reprod Open 2020; 1-21.
20. Altman DG, Vergouwe Y, Royston P ,Moons KG. Prognosis and prognostic research: validating a prognostic model. . BMJ 2009; 338: b605.
21. Collins GS, Reitsma JB, Altman DG ,Moons KG. Transparent reporting of a multivariable prediction model for individual prognosis or diagnosis (TRIPOD): the TRIPOD statement. BMJ 2015; 350: g7594.
22. Collins GS, Ogundimu EO ,Altman DG. Sample size considerations for the external validation of a multivariable prognostic model: a resampling study. Stat Med 2016; 35: 214-26.
23. Alba AC, Agoritsas T, Walsh M, Hanna S, Iorio A, Devereaux PJ, et al. Discrimination and Calibration of Clinical Prediction Models: Users' Guides to the Medical Literature. JAMA 2017; 318: 1377-84.
24. Steyerberg EW ,Vergouwe Y. Towards better clinical prediction models: seven steps for development and an ABCD for validation. Eur Heart J 2014; 35: 1925-31.
25. Austin PC ,Steyerberg EW. Graphical assessment of internal and external calibration of logistic regression models by using loess smoothers. Stat Med 2014; 33: 517-35.
26. Van Calster B, Nieboer D, Vergouwe Y, De Cock B, Pencina MJ ,Steyerberg EW. A calibration hierarchy for risk models was defined: from utopia to empirical data. J Clin Epidemiol 2016; 74: 167-76.
27. Van Calster B, Abdallah Y, Guha S, Kirk E, Van Hoorde K, Condous G, et al. Rationalizing the management of pregnancies of unknown location: temporal and external validation of a risk prediction model on 1962 pregnancies. Hum Reprod 2013; 28: 609-16.
28. Wynants L, van Smeden M, McLernon DJ, Timmerman D, Steyerberg EW, Van Calster B, et al. Three myths about risk thresholds for prediction models. BMC Med 2019; 17: 192.
29. Vickers AJ ,Elkin EB. Decision curve analysis: a novel method for evaluating prediction models. Med Decis Making 2006; 26: 565-74.
30. Joshi M, Royuela A ,Zamora J. Proper analysis in clinical trials: how to report and adjust for missing outcome data. BJOG 2013; 120: 915-19.
31. Ramspek CL, Jager KJ, Dekker FW, Zoccali C ,van Diepen M. External validation of prognostic models: what, why, how, when and where? Clin Kidney J 2021; 14: 49-58.
32. Ooi S, De Vries B ,Ludlow J. How do the M4 and M6 models perform in an Australian pregnancy of unknown location population? Aust N Z J Obstet Gynaecol 2021; 61: 100-05.
33. NICE guideline [NG126]. https://www.nice.org.uk/guidance/ng126 (Accessed September 14, 2021) 2019.

For peer review only

Figure Legends

Figure 1. Flow chart showing the inclusion of patients in the validation cohort and the sensitivity analysis. PUL, pregnancy of unknown location; hCG, human chorionic gonadotropin; IUP, intrauterine pregnancy.

Figure 2. (A) The calibration plot shows that the curve for M6NP, with pointwise 95% confidence interval (shaded blue area), are close to the line at 45 degrees up to predicted probabilities of 0.20. This indicates that risk predictions for ectopic pregnancies are accurate in this range. When the predicted risk is 40% however the observed proportion of ectopic pregnancy is ~50% which means that risk of ectopic pregnancy is underestimated. (B) M4 underestimates risk of ectopic pregnancy up to estimates of 0.40 as the curve is clearly above the line at 45 degrees in this range.

Figure 3. Decision curve analysis for M4 (dashed red line) and M6NP (blue line). The high risk threshold is above which a pregnancy of unknown location is classified as high risk (positive) of ectopic pregnancy. M6NP has a higher Net Benefit compared to M4 and default strategies of assuming all (dashed green line) or none (grey line) of PUL as high risk over the entire range of thresholds. M6NP thus has utility at any threshold as compared to M4 that has no utility ≤5%.

For peer review only

518

Table 1. Descriptive statistics of patients in the validation cohort and patients lost to follow-up.

Characteristics	Validation cohort *n*=1061	Lost to follow-up *n*=15
Age (years)		
Median (Q1;Q3)	31 (26;35)	33 (29;36)
Range	15–49	20–44
First hCG value (IU/L)		
Median (Q1;Q3)	703 (210;2312)	640 (272;6325)
Range	7–180000	19–2800
Second hCG value (IU/L)		
Median (Q1;Q3)	635 (150;2700)	810 (120:8100)
Range	1–180000	21–3500
hCG ratio (second hCG/first hCG)		
Median (Q1;Q3)	0.94 (0.45;1.59)	1.1 (0.79;1.53)
Range	0.06–5.65	0.23–1.79
Hours between two hCG samples, n (%)		
<24	0 (0)	0 (0)
24–39	141 (13)	2 (13)
40–56	751 (71)	7(47)
57–72	169 (16)	3 (20)
>72	0 (0)	0 (0)
Type of PUL, n (%)		
True PUL	793 (75)	11 (73)
Probable IUP	239 (22)	4 (27)
Probable EP	29 (3)	0 (0)
Vaginal bleeding, n (%)	723 (68)	8 (53)
Prior ectopic pregnancy, n (%)	76 (7)	1 (6)
IUD, n (%)	30 (3)	0 (0)

hCG, human chorionic gonadotropin; Q1, first interquartile; Q3, third interquartile; PUL, pregnancy of unknown location; IUP, intrauterine pregnancy, EP, ectopic pregnancy; IUD, intrauterine device. Because of rounding the summarised percentage sometimes are not exactly 100%.

519

For peer review only

Table 2. Classification accuracy of the M6NP and M4 at the 2.5, 5 and 10% threshold.

Threshold	Accuracy measure	M6NP	M4	*P*-value
2.5%	Sensitivity	97 (94–99)	91 (88–95)	<0.01
	False positive rate	61 (58–65)	43 (40–46)	<0.01
	Negative predictive value	98 (96–99)	96 (94–97)	<0.01
	Percentage classified as low risk	31 (28–34)	46 (44–50)	<0.01
5% (used in previous studies)	Sensitivity	95 (92–98)	85 (81–90)	<0.01
	False positive rate	50 (47–54)	37 (34–40)	<0.01
	Negative predictive value	97 (96–99)	94 (92–96)	<0.01
	Percentage classified as low risk	40 (37–42)	52 (50–55)	<0.01
10%	Sensitivity	91 (87–94)	80 (75–85)	<0.01
	False positive rate	39 (36–43)	30 (37–34)	<0.01
	Negative predictive value	96 (94–98)	92 (90–94)	<0.01
	Percentage classified as low risk	49 (46–52)	58 (56–61)	<0.01

Reported as percentage (95% confidence interval)

For peer review only

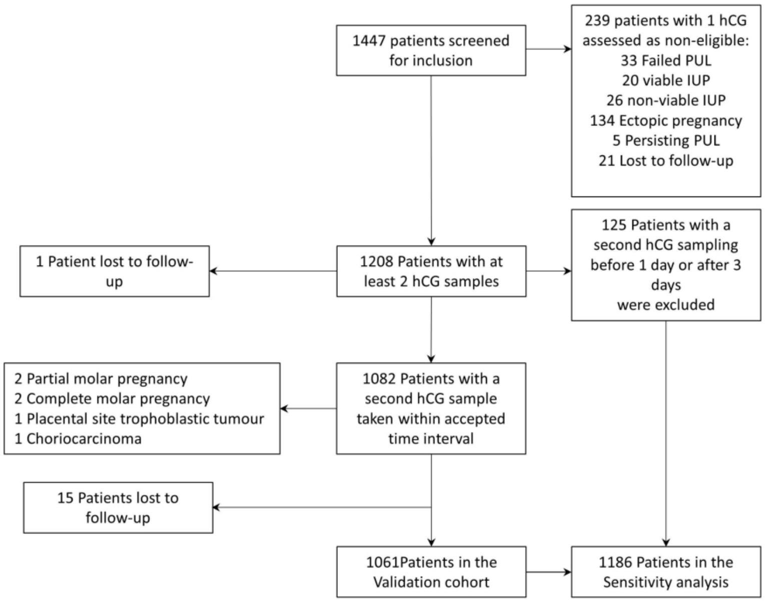

Flow chart showing the inclusion of patients in the validation cohort and the sensitivity analysis. PUL, pregnancy of unknown location; hCG, human chorionic gonadotropin; IUP, intrauterine pregnancy.

253x190mm (300 x 300 DPI)

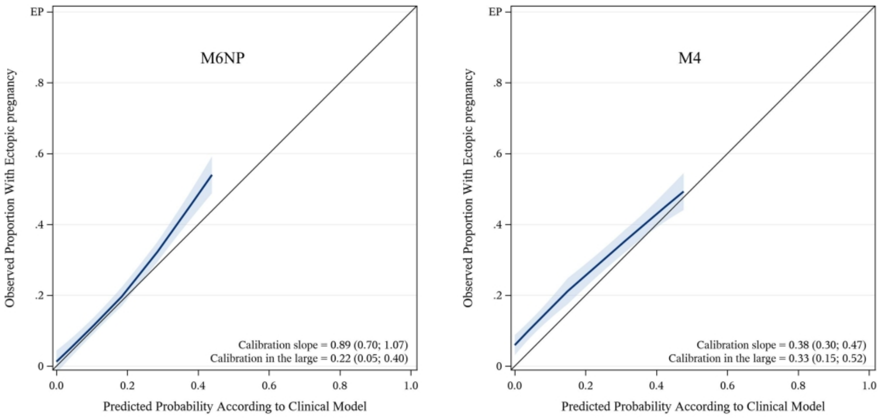

(A) The calibration plot shows that the curve for M6NP, with pointwise 95% confidence interval (shaded blue area), are close to the line at 45 degrees up to predicted probabilities of 0.20. This indicates that risk predictions for ectopic pregnancies are accurate in this range. When the predicted risk is 40% however the observed proportion of ectopic pregnancy is ~50% which means that risk of ectopic pregnancy is underestimated. (B) M4 underestimates risk of ectopic pregnancy up to estimates of 0.40 as the curve is clearly above the line at 45 degrees in this range.

254x190mm (307 x 307 DPI)

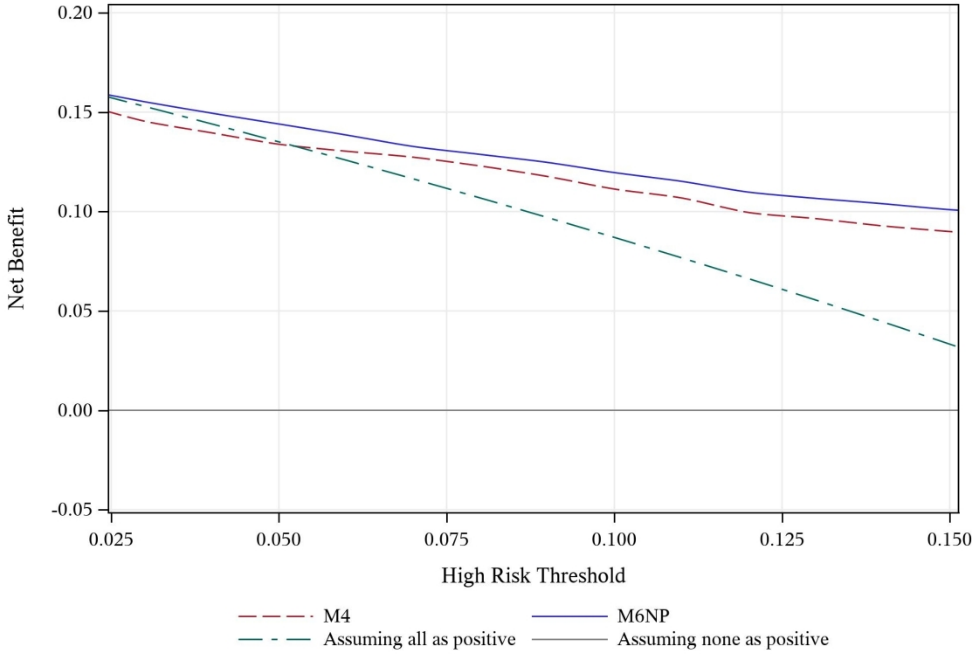

Decision curve analysis for M4 (dashed red line) and M6NP (blue line). The high risk threshold is above which a pregnancy of unknown location is classified as high risk (positive) of ectopic pregnancy. M6NP has a higher Net Benefit compared to M4 and default strategies of assuming all (dashed green line) or none (grey line) of PUL as high risk over the entire range of thresholds. M6NP thus has utility at any threshold as compared to M4 that has no utility ≤5%.

169x127mm (300 x 300 DPI)

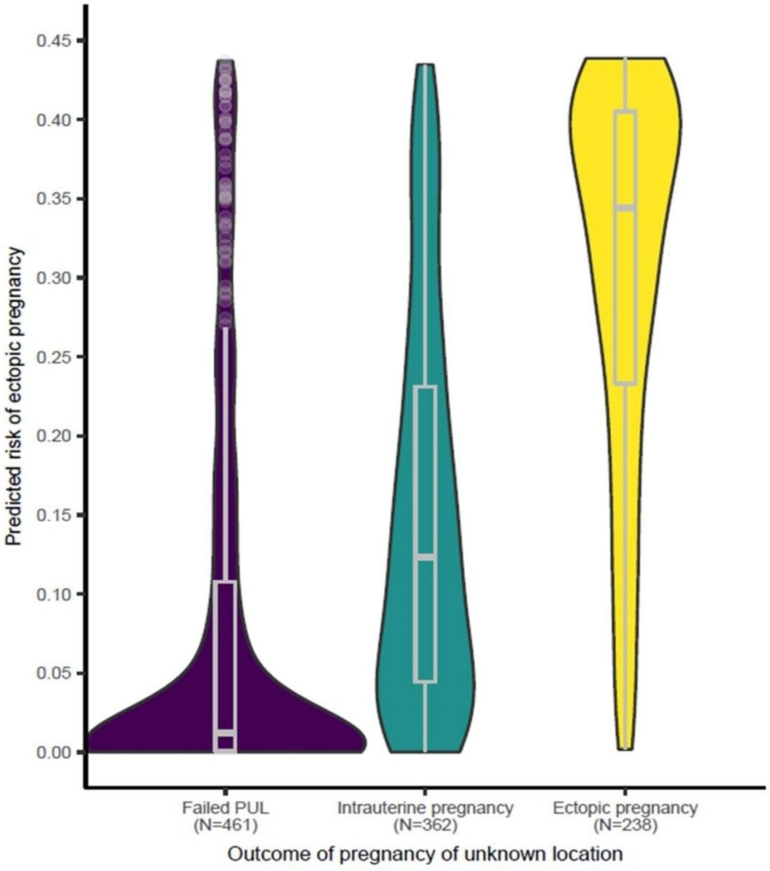

Supplemental Figure 1. The violin plot shows the full distribution of the predicted probabilities by M6NP of an ectopic pregnancy within each outcome of a pregnancy of unknown location in the validation cohort. The boxes inside the violin plot express the median (Q1; Q3). The plot indicates that less than 50% of failed PUL and 75% of IUP rendered risk estimates ≥ 5% of an EP by M6NP. This equals the proportion being misclassified as ectopic pregnancies. PUL, pregnancy of unknown location; Q1, first quartile; Q3, third quartile.

114x114mm (300 x 300 DPI)

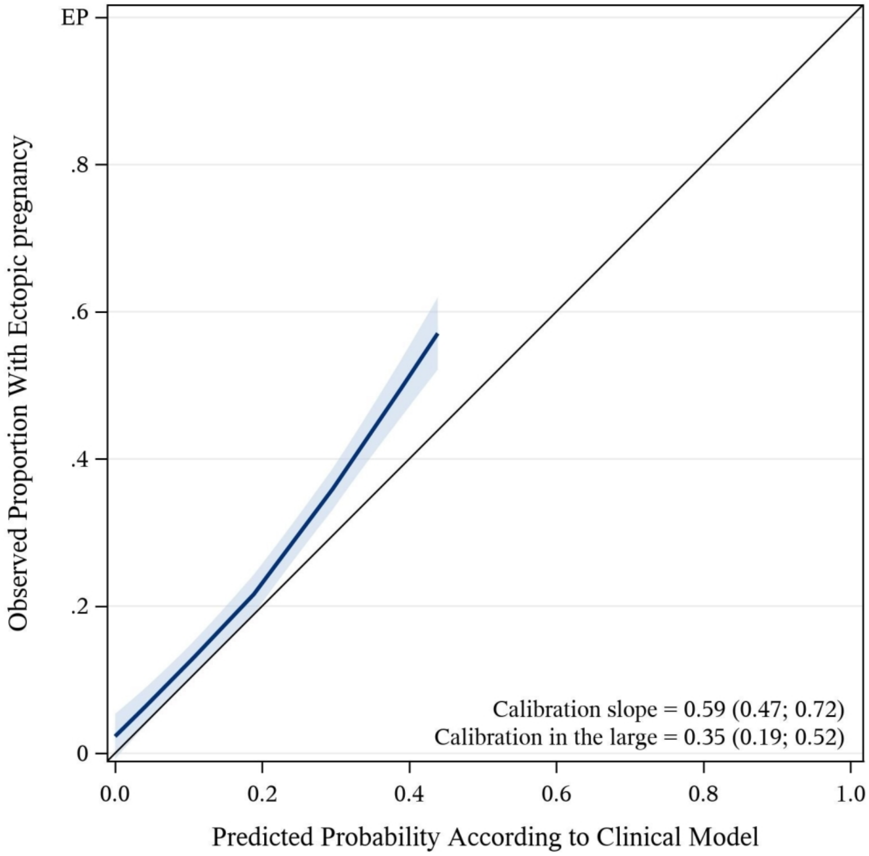

(A) The calibration plot shows the agreement between the predicted and observed probability of ectopic pregnancy by M6NP (blue line).

149x137mm (300 x 300 DPI)

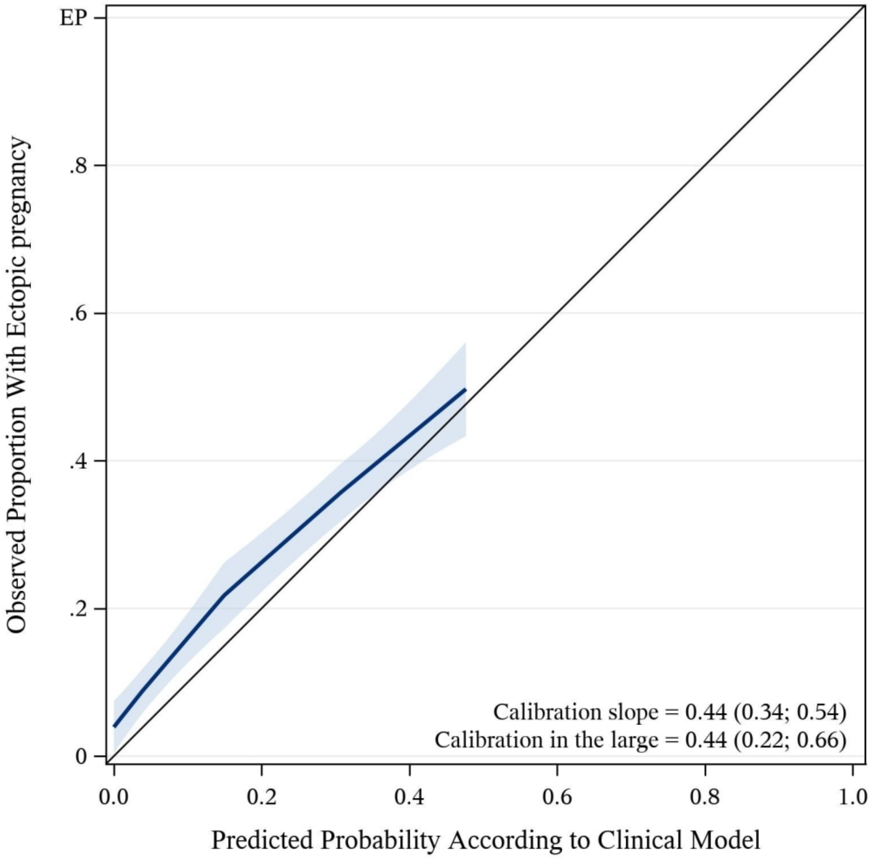

(B) The calibration plot shows the agreement between the predicted and observed probability of ectopic pregnancy by M4 (blue line).

148x137mm (300 x 300 DPI)

Supplemental

Supplemental Table 1. Covariates used in the original M4 [1] and M6NP [2] cohorts.

Model	Covariate	Description
M4	log(average hCG)	Log transformation of (first hCG + second hCG)/2
M4	centered hCG ratio	Centering transformation of (second hCG /first hCG-1.17)
M4	(centered hCG ratio)^2	The quadratic effect was added because the linearity for the logit assumption was violated for hCG ratio.
M6NP	log(initial hCG)	Log transformation of the first hCG
M6NP	log(hCG ratio)	Log transformation of (second hCG/first hCG)
M6NP	[log(hCG ratio)]^2	Square of the log transformed hCG ratio

Log refers to the natural logarithm. hCG = human chorionic gonadotropin.

[a]Average hCG ratio in the original M4 cohort. Centering reduced the correlation between hCG ratio and its square from 0.96 to 0.59.

For peer review only

Linear predictor functions for M4 where IUP was reference category [1]:

$$z_1 = log\left(\frac{P(FPUL)}{P(IUP)}\right)$$

$$= +5.8803$$

$$-5.5594 \times (hCG\ ratio - 1.17)$$

$$+2.0504 \times (hCG\ ratio - 1.17)^2$$

$$-1.175 \times \log(average\ hCG)$$

$$z_2 = log\left(\frac{P(EP)}{P(IUP)}\right)$$

$$= 0.3857$$

$$-0.2627 \times (hCG\ ratio - 1.17)$$

$$-3.9319 \times (hCG\ ratio - 1.17)^2$$

$$-0.0572 \times \log(average\ hCG)$$

Linear predictor functions for M6NP where EP was reference category [2]:

$$z_1 = log\left(\frac{P(FPUL)}{P(EP)}\right)$$

$$= 2.5506$$

$$-0.4242 \times log(initial\ hCG)$$

$$-2.9502 \times log(hCG\ ratio)$$

$$+2.1765 \times [log(hCG\ ratio)]^2$$

$$z_2 = log\left(\frac{P(IUP)}{P(EP)}\right)$$

$$= -3.2842$$

$$+0.4072 \times log(initial\ hCG)$$

$$+1.9238 \times log(hCG\ ratio)$$

$$+2.8952 \times [log(hCG\ ratio)]^2$$

For peer review only

By using the exponentiated linear predictor functions of M4 and M6NP respectively the probability of each outcome are computed as:

$$P(FPUL) = \frac{exp(z_1)}{1 + exp(z_1) + exp(z_2)}$$

$$P(IUPor\ EP\ ) = \frac{exp(z_2)}{1 + exp(z_1) + exp(z_2)}$$

$$P(EPor\ IUP) = \frac{1}{1 + exp(z_1) + exp(z_2)}$$

The probability of IUP is computed with the third formula when M4 is used. If M6NP is used the third formula computes the probability of EP. Log in the linear predictor functions refers to the natural logarithm. PUL, pregnancy of unknown location; FPUL, failed PUL; IUP, intrauterin pregnancy, EP, ectopic pregnancy.

Equation of Net Benefit [3]:

$\text{Net benefit} = (\text{True positive})/n \ - \ (\text{False positive})/n \cdot ((P_t/(1 - P_t))$, where true positive is the number of EP classified as high risk; false positive is the number of non-EP classified as high risk and n is the total number of patients. In the equation false positives are weighed by the odds of the selected risk threshold (P_t) for high risk classification. If a 5% risk threshold is chosen, a false positive non-EP is valued at 1/19th of a true positive EP.

References:

1. Condous G, Van Calster B, Kirk E, Haider Z, Timmerman D, Van Huffel S, et al. Prediction of ectopic pregnancy in women with a pregnancy of unknown location. Ultrasound Obstet Gynecol 2007; 29: 680-7.
2. Van Calster B, Bobdiwala S, Guha S, Van Hoorde K, Al-Memar M, Harvey R, et al. Managing pregnancy of unknown location based on initial serum progesterone and serial serum hCG levels: development and validation of a two-step triage protocol. Ultrasound Obstet Gynecol 2016; 48: 642-49.
3. Steyerberg EW, Vickers AJ, Cook NR, Gerds T, Gonen M, Obuchowski N, et al. Assessing the performance of prediction models: a framework for traditional and novel measures. Epidemiology 2010; 21: 128-38.

For peer review only

**Supplemental Table 2.** Descriptive statistics of 239 non-eligible patients.	
Age (years)	
Median (Q1; Q3)	32 (26; 36)
Range	15–46
First hCG value (IU/L)	
Median (Q1; Q3)	1400 (310; 5300)
Range	3–78000
Type of PUL, n (%)	
True PUL	88 (37)
Probable IUP	85 (36)
Probable EP	66 (28)
Vaginal bleeding, n (%)	155 (65)
Prior ectopic pregnancy, n (%)	20 (8)
IUD, n (%)	0 (0)

hCG, human chorionic gonadotropin; Q1, first interquartile; Q3, third interquartile; PUL, pregnancy of unknown location; IUP, intrauterine pregnancy, EP, ectopic pregnancy; IUD, intrauterine device. Because of rounding the summarised percentage are not exactly 100%.

For peer review only

Supplemental Table 3. Descriptive statistics of failed PUL, intrauterine pregnancies and ectopic pregnancies in the validation cohort and the original M4 and M6NP cohorts.

Outcome of PUL/variable	Validation cohort	Original M4 cohort	Original M6NP cohort
Failed PUL [a]	461 (43)	109 (55)	785 (54)
Age (Years) [b]	31 (27; 35)	30 (7)	32 (27; 36)
First hCG value (IU/L) [b]	334 (120; 1078)	N/A [c]	308 (104; 975)
Second hCG value (IU/L) [b]	158 (53; 460)	N/A [c]	143 (48; 397)
hCG ratio [b]	0.42 (0.27; 0.64)	0.35 (0.27)	0.40 (0.28; 0.58)
hCG average (IU/L) [b]	261 (93; 790)	89 (216)	N/A [d]
Intrauterine pregnancy [a]	362 (34)	76 (39)	501 (35)
Age (Years) [b]	29 (24; 33)	30 (6)	30 (24; 33)
First hCG value (IU/L) [b]	1521 (464; 5125)	N/A [c]	488 (253; 887)
Second hCG value (IU/L) [b]	2765 (983; 7900)	N/A [c]	1061 (515; 1863)
hCG ratio [b]	1.81 (1.39; 2.21)	2.12 (0.57)	2.15 (1.83; 2.50)
hCG average (IU/L) [b]	2226 (746; 6750)	727 (986)	N/A [d]
Ectopic pregnancy [a]	238 (22)	12 (6)	163 (11)
Age (Years) [b]	32 (27; 36)	32 (6.5)	32 (28; 35)
First hCG value (IU/L) [b]	810 (252; 2315)	N/A [c]	521 (200; 1220)
Second hCG value (IU/L) [b]	920 (265; 2735)	N/A [c]	604 (224; 1266)
hCG ratio [b]	1.05 (0.86; 1.31)	1.41 (0.47)	1.16 (0.96; 1.48)
hCG average (IU/L) [b]	865 (270; 2600)	621 (354)	N/A [d]

PUL, pregnancy of unknown location; hCG, human chorionic gonadotropin; Q1, first quartile; Q3, third quartile; IQR, interquartile range; N/A, not available.

[a]Reported as number (%)

[b]Reported as median (Q1; Q3) in the validation cohort and the original M6 cohort, and as median (IQR) in the original M4 cohort.

[c]The first and second hCG value were only presented as an average in the original M4 cohort.

[d]The average hCG value was not presented in the original M6 cohort.

For peer review only

**Supplemental Table 4.** Descriptive statistics of 12 ectopic pregnancies misclassified as low risk by M6NP.						
Type of PUL	First hCG (IU/L)	Second hCG (IU/L)	M6NP prediction	Site of EP	Rupture	Treatment
True PUL	470	290	FPUL	Tubal	No	Salpingotomy
Probable EP	88	42	FPUL	Tubal	Yes	Salpingectomy
True PUL	619	1948	IUP	Tubal	No	Salpingectomy
True PUL	3700	1500	FPUL	Tubal	No	Salpingectomy
True PUL	700	220	FPUL	Tubal	No	Salpingectomy
Probable EP	420	210	FPUL	Tubal	No	Salpingectomy
True PUL	10000	20000	IUP	Tubal	No	Salpingectomy
True PUL	640	240	FPUL	Tubal	No	Salpingectomy
True PUL	43	170	IUP	Tubal	No	Salpingectomy
Probable EP	56	23	FPUL	Tubal	No	Salpingectomy
True PUL	130	330	IUP	Tubal	No	salpingectomy
True PUL	230	590	IUP	Tubal	No	Salpingectomy

hCG, human chorionic gonadotropin, PUL, pregnancy of unknown location; EP, ectopic pregnancy; FPUL, failed PUL; IUP, intrauterine pregnancy.

For peer review only

**Supplemental Table 5.** Descriptive statistics of patients (n=1186) in the sensitivity analysis.	
Age (years)	
Median (Q1;Q3)	31 (26;35)
Range	15–49
First hCG value (IU/L)	
Median (Q1;Q3)	697 (210;2300)
Range	7–180000
Second hCG value (IU/L)	
Median (Q1;Q3)	615 (142;2655)
Range	1–190000
Hours between two hCG samples, n (%)	
<24	70 (6)
24–39	141 (12)
40–56	751 (63)
57–72	169 (14)
>72	55 (5)
Type of PUL, n (%)	
True PUL	880 (74)
Probable IUP	254 (21)
Probable EP	52 (4)
Vaginal bleeding, n (%)	803 (68)
Prior ectopic pregnancy, n (%)	86 (7)
IUD, n (%)	37 (3)

hCG, human chorionic gonadotropin; Q1, first interquartile; Q3, third interquartile; PUL, pregnancy of unknown location; IUP, intrauterine pregnancy, EP, ectopic pregnancy; IUD, intrauterine device. Because of rounding the summarised percentage sometimes are not exactly 100%.

For peer review only

Supplemental Table 6. Performance of M6NP and M4 at the 5% threshold in the sensitivity analysis.		
Performance measure	M6NP	M4
AUC	0.84 (0.81–0.86)	0.80 (0.77–0.83)
Sensitivity, %	93 (90–96)	83 (78–87)
False positive rate, %	49 (48–53)	36 (33–39)
Negative predictive value, %	96 (94–98)	92 (90–94)
AUC, area under the receiver operating characteristic curve. 95% confidence interval (in parentheses).		

For peer review only

Reporting checklist for prediction model development/validation.

Based on the TRIPOD guidelines.

Instructions to authors

Complete this checklist by entering the page numbers from your manuscript where readers will find each of the items listed below.

Your article may not currently address all the items on the checklist. Please modify your text to include the missing information. If you are certain that an item does not apply, please write "n/a" and provide a short explanation.

Upload your completed checklist as an extra file when you submit to a journal.

In your methods section, say that you used the TRIPODreporting guidelines, and cite them as:

Collins GS, Reitsma JB, Altman DG, Moons KG. Transparent reporting of a multivariable prediction model for individual prognosis or diagnosis (TRIPOD): The TRIPOD statement.

		Reporting Item	Page Number
Title			
	#1	Identify the study as developing and / or validating a multivariable prediction model, the target population, and the outcome to be predicted.	1
Abstract			
	#2	Provide a summary of objectives, study design, setting, participants, sample size, predictors, outcome, statistical analysis, results, and conclusions.	1
Introduction			
	#3a	Explain the medical context (including whether diagnostic or prognostic) and rationale for developing or validating the multivariable prediction model, including references to existing models.	3
	#3b	Specify the objectives, including whether the study describes the development or validation of the model or both.	4

For peer review only

Methods

Source of data	#4a	Describe the study design or source of data (e.g., randomized trial, cohort, or registry data), separately for the development and validation data sets, if applicable.	4
Source of data	#4b	Specify the key study dates, including start of accrual; end of accrual; and, if applicable, end of follow-up.	4
Participants	#5a	Specify key elements of the study setting (e.g., primary care, secondary care, general population) including number and location of centres.	4
Participants	#5b	Describe eligibility criteria for participants.	5
Participants	#5c	Give details of treatments received, if relevant	n/a, no treatment was given
Outcome	#6a	Clearly define the outcome that is predicted by the prediction model, including how and when assessed.	6
Outcome	#6b	Report any actions to blind assessment of the outcome to be predicted.	n/a, predictions and outcome assessment were done separately
Predictors	#7a	Clearly define all predictors used in developing or validating the multivariable prediction model, including how and when they were measured	6 and 7
Predictors	#7b	Report any actions to blind assessment of predictors for the outcome and other predictors.	n/a, predictions and outcome assessment were done separately
Sample size	#8	Explain how the study size was arrived at.	7
Missing data	#9	Describe how missing data were handled (e.g., complete-case analysis, single imputation, multiple imputation) with details of any imputation method.	9
Statistical analysis methods	#10a	If you are developing a prediction model describe how predictors were handled in the analyses.	n/a, validation study
Statistical analysis methods	#10b	If you are developing a prediction model, specify type of model, all model-building procedures (including any predictor selection), and method for internal validation.	n/a

For peer review only

Statistical analysis methods	#10c	If you are validating a prediction model, describe how the predictions were calculated.	7
Statistical analysis methods	#10d	Specify all measures used to assess model performance and, if relevant, to compare multiple models.	9
Statistical analysis methods	#10e	If you are validating a prediction model, describe any model updating (e.g., recalibration) arising from the validation, if done	n/a
Risk groups	#11	Provide details on how risk groups were created, if done.	n/a
Development vs. validation	#12	For validation, identify any differences from the development data in setting, eligibility criteria, outcome, and predictors.	5
Results			
Participants	#13a	Describe the flow of participants through the study, including the number of participants with and without the outcome and, if applicable, a summary of the follow-up time. A diagram may be helpful.	10
Participants	#13b	Describe the characteristics of the participants (basic demographics, clinical features, available predictors), including the number of participants with missing data for predictors and outcome.	10
Participants	#13c	For validation, show a comparison with the development data of the distribution of important variables (demographics, predictors and outcome).	10
Model development	#14a	If developing a model, specify the number of participants and outcome events in each analysis.	n/a
Model development	#14b	If developing a model, report the unadjusted association, if calculated between each candidate predictor and outcome.	n/a
Model specification	#15a	If developing a model, present the full prediction model to allow predictions for individuals (i.e., all regression coefficients, and model intercept or baseline survival at a given time point).	n/a
Model specification	#15b	If developing a prediction model, explain how to the use it.	n/a

For peer review only

Model performance	#16	Report performance measures (with CIs) for the prediction model.	11 and 12
Model-updating	#17	If validating a model, report the results from any model updating, if done (i.e., model specification, model performance).	n/a, updating not performed
Discussion			
Limitations	#18	Discuss any limitations of the study (such as nonrepresentative sample, few events per predictor, missing data).	13
Interpretation	#19a	For validation, discuss the results with reference to performance in the development data, and any other validation data	12
Interpretation	#19b	Give an overall interpretation of the results, considering objectives, limitations, results from similar studies, and other relevant evidence.	13
Implications	#20	Discuss the potential clinical use of the model and implications for future research	13 and 14
Other information			
Supplementary information	#21	Provide information about the availability of supplementary resources, such as study protocol, Web calculator, and data sets.	14 and 15
Funding	#22	Give the source of funding and the role of the funders for the present study.	15

None The TRIPOD checklist is distributed under the terms of the Creative Commons Attribution License CC-BY. This checklist can be completed online using https://www.goodreports.org/, a tool made by the EQUATOR Network in collaboration with Penelope.ai

For peer review only
